# Meta-genomic analysis of toilet waste from long distance flights; a step towards global surveillance of infectious diseases and antimicrobial resistance

**DOI:** 10.1038/srep11444

**Published:** 2015-07-10

**Authors:** Thomas Nordahl Petersen, Simon Rasmussen, Henrik Hasman, Christian Carøe, Jacob Bælum, Anna Charlotte Schultz, Lasse Bergmark, Christina A. Svendsen, Ole Lund, Thomas Sicheritz-Pontén, Frank M. Aarestrup

**Affiliations:** 1 Department of Systems Biology, Technical University of Denmark, Kgs. Lyngby, Denmark; 2 National Food Institute, Technical University of Denmark, Kgs. Lyngby, Denmark

## Abstract

Human populations worldwide are increasingly confronted with infectious diseases and antimicrobial resistance spreading faster and appearing more frequently. Knowledge regarding their occurrence and worldwide transmission is important to control outbreaks and prevent epidemics. Here, we performed shotgun sequencing of toilet waste from 18 international airplanes arriving in Copenhagen, Denmark, from nine cities in three world regions. An average of 18.6 Gb (14.8 to 25.7 Gb) of raw Illumina paired end sequence data was generated, cleaned, trimmed and mapped against reference sequence databases for bacteria and antimicrobial resistance genes. An average of 106,839 (0.06%) reads were assigned to resistance genes with genes encoding resistance to tetracycline, macrolide and beta-lactam resistance genes as the most abundant in all samples. We found significantly higher abundance and diversity of genes encoding antimicrobial resistance, including critical important resistance (e.g. *bla*_CTX-M_) carried on airplanes from South Asia compared to North America. Presence of *Salmonella enterica* and norovirus were also detected in higher amounts from South Asia, whereas *Clostridium difficile* was most abundant in samples from North America. Our study provides a first step towards a potential novel strategy for global surveillance enabling simultaneous detection of multiple human health threatening genetic elements, infectious agents and resistance genes.

Globally, infectious diseases are the cause of about 22% of all human deaths^1^. Due to increasing population density, increasing use of antimicrobial agents, disruption of wildlife habitats, and an increase in global travel and trade this number has been estimated to increase in coming years^2^,^3^. Rapid global surveillance systems for early detection of worldwide spread of zoonotic and human pathogens would enable outbreak control and epidemic prevention^4^,^5^. In addition to the direct severe consequences for human health, infectious diseases may cause an increased financial burden on health systems and may imply restriction on travel, trade, etc. Global spread of antimicrobial resistance in bacterial pathogens is another major threat against human health, yet we don’t know much about how these genes travel and spread worldwide^6^.

Current international disease surveillance system is mainly based on reports made by doctors after treatment of infected patients^7^. It has been attempted to establish regional and global networks, for the exchange of information for single or few pathogens^8^. This approach suffers from numerous limitations, such as limited number of sites and target pathogens, as well as serious time-delays in reporting^9^. This has prompted attempts to initiate information platforms for exchange of data, based on reporting of clinical symptoms or Internet based data mining^10^. However, this will neither give pathogen nor gene specific data, and is still based on clinical disease symptoms.

As the current routine is mainly based on individual pathogens, not necessarily capturing all disease threats, for instance antimicrobial resistance, where the bulk of resistance genes may be present in the commensal bacterial flora^11^. This is often insufficient and allows infectious agents and resistance genes to spread and infect large populations before they are even recognized^4^,^5^,^12^,^13^. Furthermore, most current surveillance typically originates from developed countries and emerging outbreaks are often not detected until they appear there^2^. All this results in a delayed and potentially biased global surveillance picture, where any taken actions often are too late^2^.

As the cost of Next Generation Sequencing (NGS) has decreased this technology has become available for routine use^14^. The technology is applicable for complete sequencing of entire microbial communities^15^ and directly on clinical samples^16^. It has also been argued that the technology might have major implications for especially developing countries where NGS as a one-fits-all tool could enable these countries to avoid the establishment of more complex diagnostic methods as those already established in developed countries^14^. NGS combined with novel hot-spot sampling points could potentially provide simultaneous diagnosis of all known pathogens and resistance genes. As international airplane flights are known to be a major route of transmission for infectious diseases^17^, airports have previously been suggested as potential control points for limiting the spread of disease outbreaks^18^. In the past airline travel has been restricted to a narrow demographic. Due to dramatic improvements in cost, availability, logistics and airline networks, this is no longer the case, and airline travel is expanding rapidly to all segments of society and to regions previously considered remote. Clearly there are still biases in the population representation in airline traffic, however it is strategic to implement antimicrobial surveillance with modes of transport since they represent transmission of antimicrobial resistance and infectious diseases, and air travel is an important avenue to address, for which surveillance protocols are currently weak or non-existent. In 2012 approximately 1.11 billion passengers travelled on an international flight; a number expected to increase beyond 1.45 billion in 2016^19^. Thus, airports offer a potentially optimal site for global surveillance.

Here we investigated toilet waste from 18 international airplanes arriving in Copenhagen, Denmark, by complete sequencing of subsequent bioinformatics analysis quantifying the occurrence of all known antimicrobial resistance genes, as well as selected pathogens (Fig. 1). The prevalence of norovirus was also determined.

## Results

### Sequencing reads and phylogenetic clustering

An average of 18.6 Gigabytes (Gb) (ranging between 14.8 and 25.7 Gb) of raw Illumina paired end sequence data was generated. The sequencing reads were cleaned, trimmed and mapped against reference sequence databases (Tables S1–4).

An average of 106,839 (0.06%) reads were assigned to resistance genes, while on average 48.7%, 9.0%, 0.17% and 0.24% were assigned to MetaHit draft genomes, complete bacterial genomes, plasmids and human, respectively. An average of 41.8% of the reads could not be assigned to any reference sequence. Rarefaction of the reads mapping to complete bacterial genomes showed a strong degree of saturation in the sequence data suggesting that sufficient amount of data has been generated (Figure S2).

Clustering of samples based on bacterial abundance profiles showed separation of the airplane samples into several clusters, but with an overall association according to geography (Fig. 2A). Tetracycline, macrolide and beta-lactam resistance genes were the most abundant in all samples (Fig. 2B) and clustering according to resistance gene class abundance was also to some degree associated with geography. At the individual gene level (Figure S3) clustering also indicated an overall association according to geography. As expected the *strA* and *strB* genes, normally being genetically linked, clustered together, while clustering of *floR* and *sul1* genes can be explained through the association with class I integrons. The grouping of *tet*(M), *tet*(O), *tet*(Q) and *tet*(W) is likely due to the general high abundance of these genes across all samples.

### Abundance of antimicrobial resistance genes

Compared to North America we found significantly higher abundance of antibiotic resistance genes among samples from South Asia, North Asia and all Asian samples combined (p-values 0.003; 0.005; 0.001) (Fig. 3A). Additionally the resistance gene richness was higher in samples from South Asia and all Asian samples combined, compared to North America (p-values 0.04 and 0.03) (Fig. 3A). In general an association between resistance gene abundance and richness was observed (Pearson Correlation Coefficient 0.69, Fig. 3B). These observations suggest a general independence of the individual genes and gene classes and an overall selection of a higher prevalence and diversity of resistance genes in mainly South Asian compared to American samples.

In-depth analysis of the abundance of individual resistance genes showed that in total 31 resistance genes were found to have significant differences (FDR < 0.05) based on geographical origin (Table S5). Comparing South Asian versus North American samples showed significantly higher abundances of *bla*_CTX-M_, *qnrS* and the 16 S methyltransferase *npmA*. A high abundance was also observed of *floR* (florphenicol/chloramphenicol resistance), *sul*2 and *sul*3 (sulphonamide resistance), *bla*_OXA_ and *bla*_TEM_ (beta-lactam resistance) and *tet*(A) and *tet*(B) (tetracycline resistance) in samples from South Asia. Additionally, the abundance of *erm*(A) and *erm*(C) (macrolide resistance) was significantly higher in North Asia compared to South Asia and of *erm*(B) significantly higher in North Asia compared to North America.

### Abundance of selected pathogens

Unique reads mapping to the human pathogens *S. enterica*, *C. difficile* and *C. jejuni* were observed in all samples (Table S6). No regional differences could be observed for *C. jejuni*, but the abundance of *S. enterica* was higher among samples from South Asia compared to both North Asia (P = 0.053) and North America (P = 0.031). In contrast, *C. difficile* was less abundant in samples from South Asia compared to North America (P = 0.0067) and North Asia (P = 0.089). Quantitative real time PCR was used to detect and quantify human noroviruses genogroup (G) I and II (Supplementary materials). Ten and 14 samples were positive for GI and GII, respectively. Significantly higher values were observed for GII norovirus in South Asian compared to North Asian and North American samples, whereas no differences were observed for GI (Table S7).

## Discussion

Antimicrobial resistance constitutes a huge global health problem^6^. It is well known that changes in diet, microbial intake and antibiotic exposure can rapidly and drastically alter the gut microbiome^20^,^21^. In addition, several recent studies have shown major differences in gut composition across geography^22^,^23^,^24^,^25^,^26^, probably related to environmental or diet differences. This suggests that the gut microbiome samples from a long-distance flight, to a large extent, reflect the microbiomes acquired at the site of boarding and not a normal “nationally” acquired flora. Strangely enough studies into the changes of the gut composition during travel are scarce, even though it has been indicated that major changes in both the gut composition and occurrence of antimicrobial resistance may occur^27^ and that foreign travel is a major risk factor for acquisition of selected resistance genes^28^. Unfortunately no relevant data for comparison to the current study exist, however, in general our observations are in agreement with recent data from WHO^6^.

Some of the most recent examples on rapid global spread of antimicrobial resistance, have been the emergence of plasmid mediated quinolone resistance, extended spectrum beta-lactamases mainly the CTX-M enzymes, and high-level aminoglycoside resistance due to 16 S methyltransferases^29^,^30^,^31^. CTX-M is considered endemic to Europe and Asia^29^, whereas reports from North America remain sporadic. In agreement with this we found a higher frequency of CTX-M and *qnrS* genes among the samples from Asia compared to North America.

The patterns observed for the selected pathogens are also supported by previous observations^32^,^33^,^34^,^35^. Thus, travellers to North America have less risk of disease caused by *Salmonella* or norovirus compared to travellers to Asia, whereas, *C. difficile* infections seems to be mainly associated with North America and Europe. However, as for resistance genes no current global surveillance exists, making comparison to other studies difficult.

There is currently only very limited information on the global occurrence and transmission of antimicrobial resistance and infectious diseases. Here we showed the feasibility of collecting and analysing long-distance airplane toilet waste for determining the occurrence of antimicrobial resistance genes and selected human pathogens. In addition to insight into the global macro-transmission of infectious agents and antimicrobial resistance, this may also open a novel avenue for simultaneous global surveillance of all infectious agents and quantification of transmission into different countries. Rather than the current surveillance approach where pathogen specific networks are established at laboratories in a limited number of countries, it might be more rapid and cost-efficient with sampling and analysis of the human waste that is transmitted around the World. Such a system is also flexible towards identifying and sampling putative hot-spots and will make it is easier to obtain samples from a new area. A number of obstacles do however, need to be overcome to make this happen. Thus, in order to provide data for action it would be necessary with large scale sequencing facilities close to the sampling site, as well as a global database for real-time exchange of data. The current study included only 18 samples and it would even with the current technologies be a challenge to sequence samples from hundreds of flights on a weekly basis. In addition, more studies are needed to determine how representative the results are of the country of origin. In general airline travel is expanding rapidly and the demographic is not as biased at present compared to years ago. A growing group of people is travelling and they can of-course act as carriers of diseases and antimicrobial resistance genes around the World.

In future efforts, the large-scale DNA sequencing performed here should be supplemented with complete sequencing of RNA viruses. However, this will still only detect the current presence of infectious agents. Detection of specific antibodies may predict previous or recent presence of infectious agents in a given population. More antibodies are generated and excreted in feces and urine that are produced in the remaining body, which recently have led to the exploration of such samples for diagnosis of diseases^36^,^37^. In the future this may also be combined with microbial sequencing to provide an even more complete picture of the prevalence and global transmission of diseases.

## Methods

### Sampling

**Figure 1:**
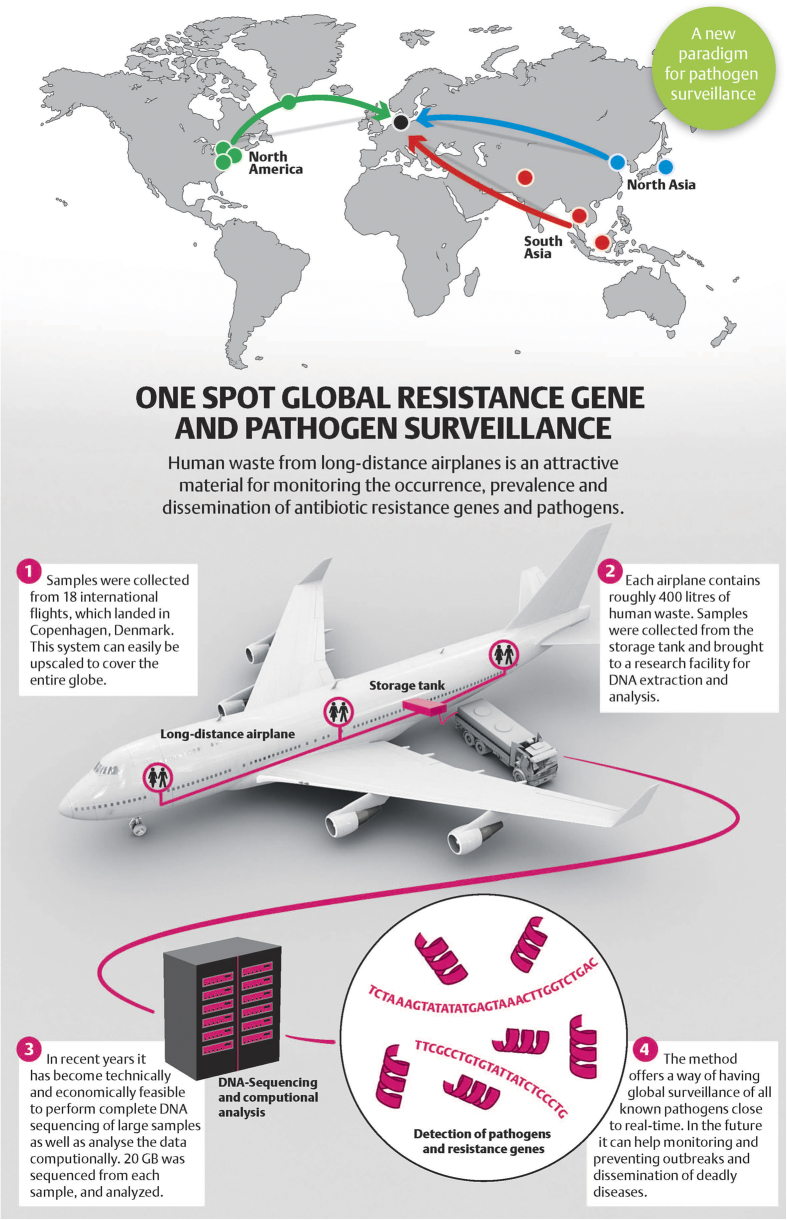
Origin of the 18 long-distance flights with destination being the international airport in Copenhagen, Denmark, as well as the analytic procedure applied. Figure created in Adobe Illustrator.

**Figure 2:**
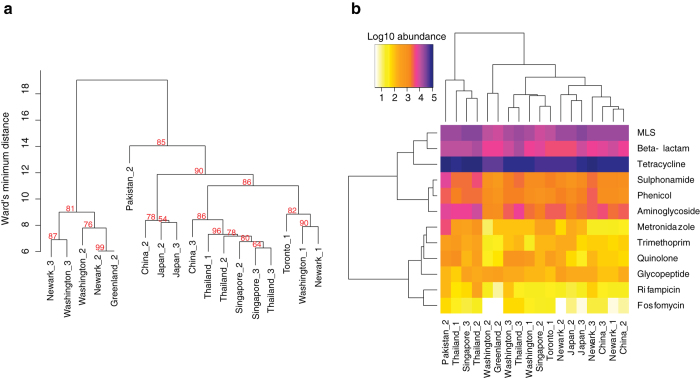
Geographical clustering of flight samples based on adjusted read abundance. a. Hierarchical clustering of flight samples based on normalized abundance of complete bacterial genomes and genomes from the human gut, bootstrap values are indicated in red and calculated using pvclust. Branch distance in the tree is Ward’s minimum distance calculated using the Lance-Williams formula. b. Heatmap showing hierarchical clustering based on the normalized abundance of resistance gene classes. The abundance is in log10 scale from white (low), yellow, orange (intermediate), magenta, blue (high). MLS: macrolide, lincosamide, streptogramin.

**Figure 3:**
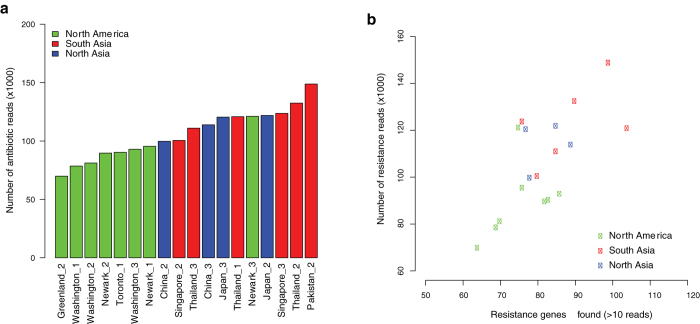
Antibiotic resistance genes detected in samples from different long-distance flights. A. Adjusted number of resistance reads identified in the flight samples. Green: North America, red: South Asia, blue: North Asia. **B.** The association between number of resistance genes found with more than 10 reads and the adjusted abundance of resistance reads per sample. Legend as in **A**.

Pooled air-toilet samples were obtained from 18 long-distance flights from Scandinavia Airline System (SAS) (www.sas.com) arriving to Copenhagen airport from nine different cities (Bangkok, Beijing, Islamabad, Newark, Kangerlussuaq, Tokyo, Toronto, and Washington DC), representing eight countries, in three different regions (North America, North and South Asia) (Fig. 1). At the sites of origin toilets were emptied, rinsed with water and disinfected prior to departure. Idu-Flight was used as disinfection agent, which is a deodorizing liquid, based on glutaraldehyde and benzalkoniumchloride. On arrival to Copenhagen the air flight waste containers were emptied by the SAS Cleaning and Service. For our purpose, one car was designated to empty the airplane waste containers, which were toughly rinsed with water prior to collecting samples. From each of the individual airplanes 6-8 waste toilets were emptied under high vacuum pressure into the special container at the service car. This procedure mixes the content thoroughly and no individual fecal clumps or toilet paper can be visually identified. Three individual ½ L samples where then collected using sterile tubes from the cars waste container and placed in a refrigerator. Subsequently, a container was rinsed with water before collecting toilet waste from another airplane. Between one and seven flights were collected during a maximum of 12 hours and immediately transported in closed containers for hazardous goods to the Technical University of Denmark and processed immediately.

### Sample handling and DNA purification

On arrival the samples were handled as outlined in Figure S1. Each individual ½ litre tube was mixed as much as possible in the tube. Four tubes, one of them supplemented with RNAlater, of 10 mL each were collected from each ½ litre tube and frozen at −80 °C. Twenty-five mL were collected from each of the three ½ L tubes obtained from a single airplane and mixed. Again four tubes of 10 mL were collected and stored as described above. The remaining approximately 35 mL combined waste was transferred to a 50 mL centrifuge tube and centrifuged at 1500 rpm for 2 minutes to remove large debris and large cells. A total of 1.2 mL was removed for conventional counting of bacteria and examination for antimicrobial resistance. A total of 30 mL supernatant was transferred to a new 50 mL tube. The supernatant was centrifuged at 10000 G for 10 min. Ten mL of the supernatant was collected and frozen at -80^o^C; the remaining was discarded. The pellet with the bacterial cells was dissolved in 500 μL phosphate buffered saline and transferred to Eppendorf Safelock™ tubes and DNA purified using a protocol including both lysozyme and lysostaphin to increase cell lysis^38^. Each of the DNA purifications were dissolved in 300 μl TE buffer and extracted with 2 rounds of phenol/chlorophorm, followed by precipitation to obtain highest possible purity. The purified DNA was frozen at −80 °C for later sequencing.

### Whole community sequencing

Samples were diluted up to a final volume of 100 μL and fragmented using a Diagenode Bioruptor, with a 30'/30' on/off program of 20 cycles. 500 ng of fragmented DNA from each sample was used to convert the extracts into Illumina-compatible DNA libraries using NEBNext library building kit for second-generation sequencing (New England Biolabs, Ipswich, MA; Cat#. E6070L). Libraries were prepared according to manufacturer’s directions with slight modifications as follows: Following end repair with incubation of 20 minutes at 12 °C followed by 15 minutes at 37 °C the samples were purified on a Qiagen Minelute silica spin-column following manufacturer’s instructions. The DNA was ligated to Illumina blunt-end adapters as described by Meyer *et al.*^39^, for 20 minutes at 20 °C and again purified on a Qiagen qiaquick silica spin-column following manufacturers instructions. Subsequently, the fill-in procedure was performed for 20 minutes at 65 °C followed by a heat inactivation step for 20 minutes at 80 °C. Libraries were then used directly for post-library indexing PCR with unique indexing for each sample^31^. Indexing PCR was done in 100 μL reactions using Phusion High-Fidelity PCR Master Mix (Thermo Fisher Scientific, Waltham, MA) and 10 μL template DNA library under the following conditions: 1) 30 seconds at 98 °C, 2) 20 seconds at 98 °C, 3) at 30 seconds at 60 °C, 4) 30 seconds at 72 °C, 5) 5 minutes at 72 °C, 6) hold at 4 °C. Step 2-4 was repeated for 4 cycles. Subsequently, the PCR products were cleaned with Qiagen qiaquick spin columns. Concentrations were measured on a Qubit fluorometer 2.0 (Life Technologies) using the dsDNA HS assay and subsequently analyzed on a Agilent 2100 Bioanalyzer (Agilent technologies). All 18 samples were pooled into one pool in equimolar concentrations for a final concentration of 9 nM. Sequencing was performed by the National High-throughput DNA Sequencing Centre at the University of Copenhagen, Denmark, on an Illumina Hiseq 2000 instrument by 100 cycles paired end for a total of 8 lanes.

### Concentration Read processing and alignment

Paired end read data was processed by mapping airtoilet fastq samples against several reference sequence databases, which can be downloaded as described in Table S2. Initially, trimming and removal of adaptor sequences was done using *cutadapt*^40^ with settings for minimum read length being 30 bp and a minimum Phred quality score of 30, to trim low-quality reads before adaptor removal (cutadapt parameter - quality-cutoff). Subsequently reads were processed through a mapping approach build on bwa mem^41^ and samtools^42^ software. Bwa mem (ver. 0.7.7-r441) was used with default settings to map reads against reference sequence databases. Throughout the mapping approach, only the most reliable hits were accepted i.e. properly paired reads were accepted provided that each read maps with an alignment length being at least 80% of the read length. As part of the initial cleaning process, potential PhiX i.e. PhiX174 control reads were removed using the procedure described above and all remaining reads are likely to have a biological origin. The read statistics for the pre-processing steps are shown in Table S1 for each of the 18 samples available. Next, reads were mapped to a number of reference sequence databases following one of two possible routes, being either chainmode or fullmode mapping. A flowchart of the two mapping procedures is shown in Figure S4. All samples were mapped against the databases that can be created from public available reference sequences. In Fullmode a resistance gene database (ResFinder) was used. In Chainmode the follow ordered list of databases were used: MetaHitAssembly, Bacteria, Plasmid, Human, Invertebrates, Protozoa and Virus. Reads that mapped to the Bacteria database were subsequently used to extract hits of pathogen specific origin. Three pathogens with the highest read counts were considered as shown in Table S6 (*Salmonella enterica* subsp. *enterica* serovar Typhimurium DT104, *Clostridium difficile* R20291, *Campylobacter jejuni* subsp. *jejuni* IA3902). In this table we only show the most confidently mapped reads and referee to them as unique meaning those where the *bwa* flags ‘AS’ and ‘XS’ differ. The flag ‘AS’ is the alignment score and ‘XS’ is the score for the second best alignment.

### Normalized abundance estimation, clustering and significance testing

The raw read counts were normalized by the total number of reads after PhiX filtering, summed to species level. All species with an abundance of more than 1,000 reads were transformed by log10 and clustered in *R* using the package pvclust using the ward method euclidean distance measure. The raw read counts of resistance genes were normalized by the total bacterial count i.e. sum of hits that could be assigned either of the to bacterial databases ‘Bacteria’ downloaded from NCBI complete genomes and ‘MetaHitassembly’ which is a collection of assembled draft genomes identified from an analysis of fecal samples from 124 European individuals^15^. Conversion tables from ResFinder identifiers i.e anti-microbial resistance genes, to gene and class level are available in Table S8. Significance testing was performed using geographical region as levels and tested individually using a Wilcoxon Rank Sum test. False Discovery Rate (FDR) was determined by using the average of three Monte Carlo simulations. The significant testing of sample-wide abundance and richness were performed using Wilcoxon Rank Sum test.

### Extraction of viral RNA and real-time RT-PCR detection of noroviruses

Five ml portions of sampled abattoir material (toilet content including glutaraldehyde and benzalkoniumchloride, pH 9.5) were mixed by vortexing with 35 ml PBS and left for shaking for 30 min at room temperature to obtain a homogeneous suspension as well as allowing viruses to detach from sample material. The mixture was centrifuged at 10,000 × G for 30 min to precipitate as much non-viral material, such as toilet paper, tissue and bacteria, as possible and the recovered supernatant were adjusted to pH 7.5 using HCL. Viruses were then precipitated from the supernatant by incubation with polyethylene glycol 8000 (80 g/L) and NaCl (17.5 g/L NaCl) during agitation (350 rpm) overnight at 4 °C. Pellet was resuspended in 0.75 ml PBS and the suspension was clarified by chloroform/butanol extraction (1:1 vol/vol), during which the mixture was vortexed for 30 sec followed by 5 min storage at room temperature before centrifugation at 10,000 × G for 15 min at 4 °C. Nucleic acids were extracted from the remaining water phase using BioMerieux reagents and miniMag apparatus (BioMerieux, Herlev, Denmark) according to the protocol of the manufacturer except for eluting the nucleic acids in 100 μl elution buffer. One-step real-time RT-PCR for the detection of NoV genogroup I and II in 2.5 μl extracted RNA was carried out as described elsewhere^43^ using a StepOnePlus™ System real-time PCR machine (Life Technologies Europe BV, Naerum, Denmark). Quantification of NoV GI and GII genome copies was done by interpolation to standard curves derived from NoV GI.3b and GII.1 RNA transcripts. Inhibition of amplification during RT-PCR detection of NoV GI and GII was evaluated by adding GI.3b and GII.1 transcripts, respectively, as internal amplification controls to each sample RNA extract and to nucleic acid-free water. The amplification efficiency was calculated by dividing the RNA transcripts recovered from the sample RNA by the RNA transcripts recovered from the nucleic acid-free water and was accepted for values above 50%. The extraction efficiency of viral nucleic acid was evaluated in all samples using Mengovirus, strain vMC_0_ (ATCC VR-1597) as internal process control. Prior to extraction, approximately 1 × 10^4^ plaque forming units of MC_0_ were spiked into the samples and nucleic acid-free water. After extraction, the levels of MC_0_ were determined by realtime RT-PCR^43^,^44^. The extraction efficiency was calculated by dividing the MC_0_ RNA recovered from the abbatoir matrix by the MC_0_ RNA recovered from the nucleic acid-free water and was accepted for values above 1%. The extraction efficiency was only used to evaluate the method performance and not incorporated into the calculation of virus concentration in the air flight samples. Significant testing was performed using Wilcoxon Rank Sum test.

### Ethics

This study was conducted in accordance with the Danish Act on scientific ethical treatment of health research, as administrated and confirmed by the Research Ethics Committees of the Capital Region of Denmark (www.regionh.dk), Journal nr.: H-14013582.

Sequence data for the flight samples are available for download through ENA.

## Additional Information

**How to cite this article**: Nordahl Petersen, T. *et al.* Meta-genomic analysis of toilet waste from long distance flights; a step towards global surveillance of infectious diseases and antimicrobial resistance. *Sci. Rep.* **5**, 11444; doi: 10.1038/srep11444 (2015).

## Supplementary Material

Supplementary Information
