# Supplementary material for: Meta-genomic analysis of toilet waste from long distance flights; a step towards global surveillance of infectious diseases and antimicrobial resistance
Source: Sci Rep. 2015 Jul 10;5:11444. doi: 10.1038/srep11444 (PMC4498435; doi:10.1038/srep11444)
Supplement: Supplementary Information [file srep11444-s1.pdf]

Meta-genomic analysis of toilet waste from long distance flights; a step towards global surveillance of infectious diseases and antimicrobial resistance

Thomas Nordahl Petersen, Simon Rasmussen, Henrik Hasman, Christian Carøe, Jacob Bælum, Anna Charlotte Schultz, Lasse Bergmark, Christina A. Svendsen, Ole Lund, Thomas Sicheritz-Pontén, Frank M. Aarestrup

Supplementary tables and figures

Sample	Number of bases (x10^9)	Number of raw reads	Cutadapt	Reads in Common	notPhiX
China 2	17.4	176183176	166353451	160021618	159902260
China 3	16.6	167573470	158336651	145039758	144894876
Japan 2	15.0	151194708	143094819	127687760	127602782
Japan 3	17.8	179736146	170510914	164240716	164129358
Thailand 1	16.1	162510852	153198042	147386846	147264464
Thailand 2	22.7	228801518	216277032	204363784	204165396
Thailand 3	19.1	192754118	182230002	175241558	175078598
Singapore 2	14.8	149414266	141035981	135471138	135358600
Singapore 3	24.0	242276662	229620624	221236902	221064678
Greenland 2	16.7	168841036	159198587	145741288	145659526
Pakistan 2	25.7	259510838	243839457	226438106	226096876
Newark 1	19.0	191612966	179508252	172032622	171893198
Newark 2	18.5	187186964	176830027	166282122	166088144
Newark 3	20.1	203297524	192958099	185951916	185826762
Washington 1	20.6	207817972	194759326	186776622	186560962
Washington 2	16.1	162990704	153105589	147024384	146865532
Washington 3	19.6	198276096	187229170	179925000	179782256
Toronto 1	15.9	161044874	152006390	146162322	145976034

Supplementary Table 1. Raw reads are the initial read count before processing. Column ‘Cutadapt’ are the read count after trimming and adaptor removal. ‘Reads in Common’ are number paired reads that remain after the cutadapt trimming process. Last column ‘notPhiX’ are the number of reads that remain after removing reads that map to PhiX174 reference sequences.

Database name	Download information
ResFinder	http://cge.cbs.dtu.dk/services/data.php
MetaHitAssembly	http://www.ncbi.nlm.nih.gov/nuccore/?term=PRJEB674 (PRJEB674 - PRJEB1046)
Bacteria	ftp://ftp.ncbi.nih.gov/genomes/Bacteria [1]
Plamid	ftp://ftp.ncbi.nih.gov/genomes/Bacteria [2]
Human	Human genome ver. GRCh37.p13
Invertebrates	ftp://ftp.ncbi.nlm.nih.gov/genbank/genomes/Eukaryotes/invertebrates/
Protozoa	ftp://ftp.ncbi.nlm.nih.gov/genbank/genomes/Eukaryotes/protozoa
Virus	ftp://ftp.ncbi.nih.gov/genomes/virus

Supplementary Table 2. Download of reference sequence data. Reference sequence information can be obtained from the url's shown in 'Download information'. [1] Only entries representing bacteria. [2] Only entries representing plasmids.

	Percentage of reads mapped to reference sequence databases					
Sample	MetaHit-Assembly	Bacteria	Plasmid	Human	Unmapped	ResFinder
China 2	50.2	9.7	0.13	0.08	39.8	0.06
China 3	50.0	9.7	0.12	0.06	40.2	0.07
Japan 2	43.6	8.7	0.17	0.27	47.2	0.07
Japan 3	50.0	7.8	0.16	0.18	42.0	0.07
Thailand 1	53.6	8.2	0.25	0.11	37.8	0.08
Thailand 2	46.3	8.5	0.20	0.12	44.9	0.07
Thailand 3	50.7	8.9	0.18	0.11	40.1	0.07
Singapore 2	47.8	8.9	0.19	0.18	42.9	0.06
Singapore 3	46.3	8.6	0.18	0.07	44.8	0.07
Greenland 2	49.7	9.8	0.15	0.12	40.2	0.04
Pakistan 2	38.4	8.5	0.11	0.06	52.9	0.07
Newark 1	49.6	9.0	0.21	1.44	39.7	0.06
Newark 2	46.4	9.3	0.11	0.26	43.9	0.05
Newark 3	46.8	9.0	0.17	0.23	43.9	0.07
Washington 1	53.8	9.6	0.21	0.28	36.1	0.05
Washington 2	49.2	8.8	0.13	0.44	41.5	0.05
Washington 3	51.1	9.8	0.18	0.13	38.7	0.06
Toronto 1	53.5	9.7	0.18	0.21	36.3	0.06
Average	48.7	9.0	0.17	0.24	41.8	0.06

Supplementary Table 3. Mapping statistics for a subset of the databases is shown in table S3. A limited amount of reads mapped to the Invertebrates, Protozoa and Virus databases and therefore percentages are only shown for the databases MetaHitAssembly, Bacteria, Plasmid and Human. Columns show the samples names, followed by reference sequence database names to which the majority of reads could be assigned. The ResFinder database contains all the resistance gene sequences and here mapping was performed in Fullmode whereas Chainmode mapping was utilized for the MetaHitAssembly, Bacteria, Plasmid and Human databases. The column ‘Unmapped’ shows the percentage of reads that could not be assigned to any database where mapping was performed in Chainmode. The average percentage of reads in shown in the last row. For each of the samples a percentage of 100 correspond to the number of reads shown in column notPhiX in table S1.

	Absolute number of reads mapped to reference sequence databases					
Sample	MetaHit-Assembly	Bacteria	Plasmid	Human	Unmapped	ResFinder
China 2	80323500	15515220	215430	135820	63694722	98706
China 3	72390218	14036912	177150	91874	58183832	101608
Japan 2	55633018	11155040	213518	344970	60241062	84290
Japan 3	81960564	12826418	269454	294936	68760230	117862
Thailand 1	78957422	12074186	367200	159932	55673498	113584
Thailand 2	94506024	17290900	410892	250448	91666392	152968
Thailand 3	88750766	15612290	316184	191068	70189794	119574
Singapore 2	64756198	12046278	252648	248766	58038434	79668
Singapore 3	102443028	19063912	396624	144494	98989104	155192
Greenland 2	72416918	14296084	225100	169492	58536814	62564
Pakistan 2	86911454	19262526	256710	136856	119488044	163114
Newark 1	85279992	15430798	364226	2473606	68305746	99294
Newark 2	77076030	15484890	177812	439452	72884144	85662
Newark 3	86873034	16684544	314088	422006	81512582	129516
Washington 1	100336712	17923640	395424	517998	67364284	96598
Washington 2	72222900	12864702	190294	651308	60918552	71606
Washington 3	91944290	17688794	330644	227124	69572606	105200
Toronto 1	78108170	14223790	270036	303910	53049342	86104
Average	81716124	15193385	285746	400226	70948288	106839

Supplementary Table 4. Columns show the samples names, followed by reference sequence database names to which the majority of reads could be assigned.

Supplementary Table 5: Normalised mean abundance and p-values and foldchange for comparison between different origins
NAM: North America; SEA: South Asia; EA: North-East Asia; AA: Asia (Sort and North-East Asia combined)

	Normalized mean abundance				p-value for comparison				Foldchange for comparison			
**Gene**	**cNAM**	**cSEA**	**cEA**	**cAA**	**pAA_NAM**	**pSEA_EA**	**pEA_NAM**	**pSEA_NAM**	**fcAA_NAM**	**fcSEA_EA**	**fcEA_NAM**	**fcSEA_NAM**
aac(3)-I	2,5	1,4	1,6	1,5	0,217	0,909	0,437	0,262	0,6	0,9	0,6	0,6
aac(3)-Ib-aac	2,8	8,7	1,3	5,8	0,964	0,240	0,549	0,755	2,0	6,8	0,5	3,1
aac(3)-II	8,2	38,2	6,0	25,3	0,184	0,199	0,587	0,139	3,1	6,4	0,7	4,7
aac(3)-IV	0,3	1,4	0,0	0,8	0,732	0,287	0,596	0,419	2,9	Inf	0,0	4,8
aac(3)-VI	0,2	0,9	0,5	0,7	0,632	1,000	0,602	0,832	3,3	1,7	2,3	3,9
aac(6')-aph(2	200,0	385,9	1140,7	687,8	0,027	0,114	0,008	0,228	3,4	0,3	5,7	1,9
aac(6')-I	18,4	24,2	21,9	23,3	0,829	0,914	0,683	1,000	1,3	1,1	1,2	1,3
aac(6')-II	28,1	67,6	17,5	47,6	0,897	0,257	0,214	0,491	1,7	3,9	0,6	2,4
aac(6')I	7,9	26,5	1,1	16,3	0,503	0,042	0,342	0,081	2,1	24,7	0,1	3,4
aadA	1069,2	1814,8	1011,8	1493,6	0,360	0,038	0,933	0,142	1,4	1,8	0,9	1,7
aadB	9,1	17,9	24,4	20,5	0,173	0,352	0,048	0,662	2,3	0,7	2,7	2,0
aadD	9,4	13,4	85,7	42,3	0,396	0,069	0,014	0,557	4,5	0,2	9,1	1,4
ant(3'')-Ih-aa	14,4	40,2	9,5	27,9	0,237	0,010	0,570	0,020	1,9	4,2	0,7	2,8
ant(6)-I	157,5	316,9	119,2	237,8	0,515	0,010	0,109	0,020	1,5	2,7	0,8	2,0
ant(9)-I	0,2	2,4	0,0	1,5	1,000	0,540	0,596	0,832	6,6	Inf	0,0	11,0
aph(2'')-I	28,1	21,0	23,5	22,0	0,315	0,762	0,683	0,282	0,8	0,9	0,8	0,7
aph(3')-I	20,2	54,4	26,3	43,2	0,012	0,114	0,109	0,020	2,1	2,1	1,3	2,7
aph(3')-II	2,4	9,9	1,8	6,7	0,094	0,087	0,428	0,075	2,7	5,5	0,7	4,1
aph(3')-III	400,7	1258,3	340,8	891,3	0,360	0,038	0,461	0,043	2,2	3,7	0,9	3,1
aph(3')-VI	2,1	0,3	0,0	0,2	0,871	0,540	0,596	1,000	0,1	Inf	0,0	0,1
aph(3')-VII	0,2	0,4	1,5	0,8	0,329	0,359	0,147	0,832	3,5	0,2	6,5	1,5
aph(3')-XV	4,5	0,6	0,6	0,6	0,015	1,000	0,094	0,037	0,1	1,0	0,1	0,1
aph(4)-I	1,1	2,8	2,8	2,8	0,779	0,896	0,911	0,808	2,5	1,0	2,5	2,5
aph(6)-I	2,9	1,5	1,0	1,3	0,733	0,719	1,000	0,617	0,5	1,5	0,3	0,5
apmA	0,2	0,0	0,0	0,0	0,314	NA	0,596	0,470	0,0	NA	0,0	0,0
ARR	39,1	83,2	28,3	61,2	0,315	0,019	0,808	0,081	1,6	2,9	0,7	2,1
blaACC	0,3	0,0	0,0	0,0	0,314	NA	0,596	0,470	0,0	NA	0,0	0,0
blaACI	36,1	172,2	41,0	119,7	0,173	0,171	0,808	0,020	3,3	4,2	1,1	4,8
blaACT	9,8	10,7	1,8	7,1	0,689	0,165	0,200	0,755	0,7	5,8	0,2	1,1
blaADC	0,5	0,0	0,0	0,0	0,122	NA	0,361	0,244	0,0	NA	0,0	0,0
blaBIL	0,0	0,6	0,0	0,4	0,222	0,287	NA	0,112	Inf	Inf	NA	Inf
blaCARB	370,6	477,7	416,6	453,3	0,408	0,914	0,570	0,491	1,2	1,1	1,1	1,3
blaCFE	0,3	0,7	0,7	0,7	0,393	1,000	0,602	0,419	2,6	1,0	2,5	2,6
blaCKO	0,2	0,3	0,0	0,2	0,871	0,540	0,596	1,000	0,8	Inf	0,0	1,3
blaCMG	0,5	0,6	0,0	0,4	0,837	0,287	0,596	0,530	0,7	Inf	0,0	1,2
blaCMY	6,9	76,6	6,1	48,4	0,146	0,257	0,570	0,108	7,1	12,5	0,9	11,2
blaCTX-M	2,2	57,1	5,0	36,3	0,020	0,171	0,218	0,013	16,5	11,3	2,3	26,0
blaDES	0,0	0,4	0,0	0,2	0,434	0,540	NA	0,312	Inf	Inf	NA	Inf
blaDHA	11,6	25,0	4,0	16,6	0,197	0,914	0,214	0,401	1,4	6,3	0,3	2,2
blaGES	0,0	0,3	0,0	0,2	0,434	0,540	NA	0,312	Inf	Inf	NA	Inf
blaIMP	0,2	0,0	0,0	0,0	0,314	NA	0,596	0,470	0,0	NA	0,0	0,0
blaL	0,5	1,1	0,0	0,6	1,000	0,540	0,596	0,832	1,2	Inf	0,0	2,0
blaLAT	0,0	0,3	0,0	0,2	0,434	0,540	NA	0,312	Inf	Inf	NA	Inf
blaLEN	9,2	1,6	1,1	1,4	0,486	0,741	0,526	0,638	0,1	1,5	0,1	0,2
blaMAL	0,0	0,3	0,0	0,2	0,434	0,540	NA	0,312	Inf	Inf	NA	Inf
blaMIR	0,5	2,7	0,0	1,6	0,315	0,072	0,361	0,054	3,5	Inf	0,0	5,8
blaMOR	0,2	2,9	0,7	2,0	0,329	0,694	0,602	0,323	9,3	4,0	3,3	13,3
blaMOX	0,0	0,7	0,0	0,4	0,434	0,540	NA	0,312	Inf	Inf	NA	Inf
blaMUS	0,3	0,0	0,0	0,0	0,314	NA	0,596	0,470	0,0	NA	0,0	0,0
blaNPS	0,3	0,4	0,0	0,2	0,871	0,540	0,596	1,000	0,8	Inf	0,0	1,3
blaOCH	0,0	0,3	0,0	0,2	0,434	0,540	NA	0,312	Inf	Inf	NA	Inf
blaOKP-A	0,0	0,7	0,0	0,4	0,222	0,287	NA	0,112	Inf	Inf	NA	Inf
blaOKP-B	0,7	3,2	1,1	2,3	0,141	0,450	0,613	0,085	3,3	3,0	1,5	4,5
blaOXA	595,6	1253,7	847,6	1091,2	0,012	0,171	0,214	0,008	1,8	1,5	1,4	2,1
blaOXY	0,3	3,3	0,5	2,2	0,138	0,211	0,794	0,060	7,7	6,5	1,8	11,7
blaPAO	0,0	0,7	0,0	0,4	0,434	0,540	NA	0,312	Inf	Inf	NA	Inf
blaPME	0,0	0,9	0,0	0,5	0,222	0,287	NA	0,112	Inf	Inf	NA	Inf
blaSHV	15,9	80,4	7,5	51,2	0,197	0,114	0,932	0,081	3,2	10,8	0,5	5,0
blaTEM	116,1	530,0	21,3	326,5	0,274	0,010	0,683	0,043	2,8	24,8	0,2	4,6
blaVEB	0,3	0,0	0,6	0,2	1,000	0,307	0,602	0,470	0,9	0,0	2,1	0,0
blaZ	1,5	0,3	0,6	0,4	0,153	0,761	0,507	0,153	0,3	0,6	0,4	0,2
blaZEG	0,0	0,0	0,5	0,2	0,434	0,307	0,216	NA	Inf	0,0	Inf	NA
car(A)	1,3	0,9	1,1	1,0	0,916	0,512	0,636	0,518	0,8	0,9	0,8	0,7
cat	568,4	422,7	610,7	497,9	0,696	0,610	0,570	0,950	0,9	0,7	1,1	0,7
catA	8,1	55,3	9,4	36,9	0,213	0,038	0,496	0,013	4,6	5,9	1,2	6,9
catB	307,3	412,1	260,1	351,3	0,633	0,171	0,683	0,282	1,1	1,6	0,8	1,3
catP	53,4	100,8	67,6	87,5	0,146	0,352	0,683	0,081	1,6	1,5	1,3	1,9
catQ	0,7	1,7	0,5	1,2	0,561	0,471	1,000	0,408	1,7	3,4	0,7	2,3
catS	141,9	64,6	170,1	106,8	0,101	1,000	0,683	0,043	0,8	0,4	1,2	0,5
cepA	162,3	222,9	135,7	188,0	0,173	0,067	0,570	0,142	1,2	1,6	0,8	1,4
cfiA	10,8	45,3	25,3	37,3	0,023	0,610	0,106	0,045	3,5	1,8	2,4	4,2
cfr	0,7	0,9	0,0	0,5	0,855	0,287	0,361	0,808	0,7	Inf	0,0	1,2
cfxA	4613,5	8315,6	5708,9	7272,9	0,027	0,114	0,461	0,008	1,6	1,5	1,2	1,8
cml	11,1	12,0	14,3	12,9	0,897	0,610	0,570	0,852	1,2	0,8	1,3	1,1
cmlA	145,6	274,4	270,3	272,8	0,009	0,762	0,028	0,043	1,9	1,0	1,9	1,9
cmx	14,5	19,4	24,9	21,6	0,515	0,610	0,283	0,950	1,5	0,8	1,7	1,3
cphA	0,2	0,0	0,0	0,0	0,314	NA	0,596	0,470	0,0	NA	0,0	0,0
dfrA	41,4	174,5	35,9	119,1	0,055	0,019	1,000	0,003	2,9	4,9	0,9	4,2
dfrAb	1,0	0,8	1,7	1,2	0,832	1,000	1,000	0,821	1,2	0,5	1,7	0,9
dfrB	0,0	0,6	0,7	0,6	0,116	1,000	0,216	0,112	Inf	0,8	Inf	Inf
dfrC	0,0	0,7	0,0	0,4	0,222	0,287	NA	0,112	Inf	Inf	NA	Inf
dfrD	10,5	4,0	6,7	5,1	1,000	0,521	1,000	1,000	0,5	0,6	0,6	0,4

dfrG	26,8	67,5	71,4	69,1	0,068	0,610	0,073	0,228	2,6	0,9	2,7	2,5
dfrK	0,7	0,0	0,0	0,0	0,122	NA	0,361	0,244	0,0	NA	0,0	0,0
ere(A)	11,9	33,5	35,3	34,2	0,004	0,914	0,004	0,059	2,9	0,9	3,0	2,8
ere(B)	2,7	5,5	1,7	4,0	0,511	0,733	0,394	0,808	1,5	3,2	0,6	2,0
erm(33)	25,0	11,5	50,7	27,2	0,762	0,019	0,214	0,142	1,1	0,2	2,0	0,5
erm(35)	10,5	21,0	14,1	18,2	0,068	0,171	0,461	0,043	1,7	1,5	1,3	2,0
erm(36)	0,0	0,6	0,0	0,4	0,434	0,540	NA	0,312	Inf	Inf	NA	Inf
erm(39)	0,3	0,0	0,0	0,0	0,314	NA	0,596	0,470	0,0	NA	0,0	0,0
erm(42)	8,5	27,2	6,6	19,0	0,168	0,019	0,799	0,024	2,2	4,1	0,8	3,2
erm(A)	578,6	214,7	1030,2	540,9	0,573	0,010	0,154	0,043	0,9	0,2	1,8	0,4
erm(B)	2692,5	3262,8	6120,8	4406,0	0,101	0,114	0,004	0,755	1,6	0,5	2,3	1,2
erm(C)	102,1	47,2	423,2	197,6	1,000	0,010	0,073	0,181	1,9	0,1	4,1	0,5
erm(F)	2913,5	4620,3	4571,4	4600,7	0,016	1,000	0,109	0,029	1,6	1,0	1,6	1,6
erm(G)	620,5	742,2	1312,6	970,3	0,274	0,257	0,073	0,852	1,6	0,6	2,1	1,2
erm(Q)	2,2	3,6	12,2	7,0	0,228	0,069	0,008	0,845	3,2	0,3	5,5	1,6
erm(T)	2966,5	2760,4	4517,3	3463,1	0,203	0,114	0,214	0,414	1,2	0,6	1,5	0,9
erm(X)	9,8	227,5	47,4	155,4	0,001	0,352	0,004	0,008	15,9	4,8	4,8	23,2
erm(Y)	67,5	118,3	105,9	113,4	0,068	0,762	0,214	0,108	1,7	1,1	1,6	1,8
fexA	0,5	4,5	0,6	2,9	0,108	0,124	0,911	0,033	6,0	8,0	1,2	9,2
floR	175,8	582,2	217,1	436,2	0,006	0,010	0,368	0,001	2,5	2,7	1,2	3,3
fosA	17,4	35,0	9,4	24,7	0,237	0,038	0,933	0,108	1,4	3,7	0,5	2,0
hugA	0,5	1,8	0,0	1,1	0,538	0,540	0,361	0,928	2,1	Inf	0,0	3,5
lmr(A)	0,0	0,0	0,6	0,2	0,434	0,307	0,216	NA	Inf	0,0	Inf	NA
lnu(A)	1,2	1,0	4,7	2,5	0,289	0,570	0,363	0,408	2,1	0,2	4,0	0,8
lnu(B)	6,1	28,2	8,6	20,3	0,016	0,019	0,570	0,001	3,3	3,3	1,4	4,6
lnu(C)	237,4	283,4	349,0	309,6	0,122	0,610	0,214	0,228	1,3	0,8	1,5	1,2
lnu(D)	1,2	3,1	4,7	3,8	0,019	0,352	0,028	0,081	3,0	0,7	3,8	2,5
lnu(F)	0,4	4,9	0,0	3,0	0,427	0,072	0,361	0,100	6,7	Inf	0,0	11,1
lsa(A)	0,4	1,7	2,6	2,0	0,196	0,719	0,220	0,323	4,6	0,7	5,8	3,8
lsa(B)	0,3	0,3	0,7	0,5	0,732	0,761	0,602	1,000	1,7	0,4	2,5	1,1
lsa(C)	38,7	50,1	40,0	46,1	0,460	0,476	1,000	0,282	1,2	1,3	1,0	1,3
mecA	0,5	0,0	2,0	0,8	0,538	0,307	0,911	0,244	1,6	0,0	3,9	0,0
mef(A)	1164,2	1648,4	1517,7	1596,1	0,203	1,000	0,214	0,414	1,4	1,1	1,3	1,4
mef(B)	1,8	25,0	0,5	15,2	0,429	0,048	0,394	0,075	8,5	49,4	0,3	14,0
mph(A)	34,4	228,0	29,8	148,7	0,021	0,010	0,808	0,001	4,3	7,6	0,9	6,6
mph(C)	0,7	0,0	0,6	0,2	0,272	0,307	1,000	0,127	0,3	0,0	0,8	0,0
mph(E)	867,0	1132,8	892,6	1036,7	0,360	0,257	0,933	0,228	1,2	1,3	1,0	1,3
msr(A)	0,4	0,4	0,7	0,5	1,000	0,761	0,911	0,928	1,1	0,5	1,6	0,8
msr(C)	2,4	14,8	5,2	11,0	0,067	1,000	0,142	0,133	4,5	2,8	2,2	6,1
msr(D)	1141,1	1612,8	1429,8	1539,6	0,146	0,914	0,073	0,491	1,3	1,1	1,3	1,4
msr(E)	1605,5	2272,4	1658,8	2027,0	0,237	0,257	0,808	0,142	1,3	1,4	1,0	1,4
nimA	6,2	6,8	3,6	5,5	1,000	1,000	1,000	1,000	0,9	1,9	0,6	1,1
nimB	3,6	0,3	1,6	0,8	0,097	0,238	0,538	0,063	0,2	0,2	0,4	0,1
nimC	0,7	2,1	0,0	1,3	0,538	0,540	0,361	0,928	1,8	Inf	0,0	3,0
nimD	23,6	106,5	55,1	85,9	0,505	0,521	0,808	0,228	3,6	1,9	2,3	4,5
nimE	45,3	618,4	35,7	385,3	0,624	0,109	0,441	0,181	8,5	17,3	0,8	13,7
nimF	1,1	1,0	13,7	6,1	0,337	0,139	0,107	0,936	5,5	0,1	12,5	0,9
nimH	0,2	0,6	0,0	0,3	1,000	0,540	0,596	0,832	1,6	Inf	0,0	2,6
nimJ	0,8	47,9	0,0	28,7	0,329	0,149	0,596	0,106	36,5	Inf	0,0	60,9
norA	0,5	0,0	0,0	0,0	0,122	NA	0,361	0,244	0,0	NA	0,0	0,0
npmA	0,0	2,9	7,4	4,7	0,005	0,331	0,010	0,012	Inf	0,4	Inf	Inf
ole(B)	1,2	1,5	0,7	1,2	0,960	0,631	0,686	0,886	1,0	2,0	0,6	1,2
ole(C)	0,3	0,3	0,7	0,5	0,732	0,761	0,602	1,000	1,7	0,4	2,6	1,1
oqxA	58,0	70,6	10,7	46,6	0,897	0,010	0,461	0,414	0,8	6,6	0,2	1,2
oqxB	122,1	192,0	47,8	134,3	0,460	0,010	0,933	0,228	1,1	4,0	0,4	1,6
otr(A)	1,0	0,9	0,0	0,6	0,597	0,149	0,220	1,000	0,5	Inf	0,0	0,9
otr(C)	0,0	0,3	0,0	0,2	0,434	0,540	NA	0,312	Inf	Inf	NA	Inf
penA	0,2	0,3	0,0	0,2	0,871	0,540	0,596	1,000	0,7	Inf	0,0	1,2
qepA	0,3	5,2	0,5	3,3	0,464	0,896	0,794	0,419	12,6	10,2	1,9	19,7
QnrA	0,2	0,0	0,0	0,0	0,314	NA	0,596	0,470	0,0	NA	0,0	0,0
QnrB	2,1	19,8	1,2	12,4	0,156	0,067	1,000	0,041	5,9	16,0	0,6	9,4
QnrD	42,0	120,7	8,4	75,8	0,034	1,000	0,073	0,108	1,8	14,4	0,2	2,9
QnrS	6,6	73,8	2,2	45,2	0,156	0,013	0,507	0,011	6,9	32,9	0,3	11,2
rmtD	0,8	0,0	0,0	0,0	0,047	NA	0,220	0,127	0,0	NA	0,0	0,0
spc	1,0	2,2	28,4	12,7	0,329	0,139	0,103	0,940	12,4	0,1	27,7	2,2
sph	1,0	3,9	0,0	2,3	0,538	0,540	0,361	0,928	2,3	Inf	0,0	3,8
srm(B)	1,8	3,2	1,1	2,4	0,669	0,741	0,925	0,448	1,3	3,0	0,6	1,8
str	81,3	194,7	76,1	147,2	0,203	0,171	1,000	0,059	1,8	2,6	0,9	2,4
strA	224,5	402,7	212,9	326,7	0,068	0,114	0,368	0,059	1,5	1,9	0,9	1,8
strB	174,3	441,6	258,0	368,1	0,009	0,257	0,154	0,008	2,1	1,7	1,5	2,5
sul1	822,6	1410,2	666,6	1112,8	0,696	0,067	0,368	0,181	1,4	2,1	0,8	1,7
sul2	548,7	1413,3	523,8	1057,5	0,122	0,019	0,683	0,005	1,9	2,7	1,0	2,6
sul3	71,4	148,5	61,5	113,7	0,146	0,019	0,808	0,013	1,6	2,4	0,9	2,1
tet(31)	24,8	11,6	34,2	20,6	0,696	0,038	0,109	0,059	0,8	0,3	1,4	0,5
tet(32)	2125,0	2966,2	3041,1	2996,2	0,001	0,762	0,008	0,013	1,4	1,0	1,4	1,4
tet(33)	40,8	94,3	36,1	71,1	0,573	0,762	0,683	0,662	1,7	2,6	0,9	2,3
tet(35)	0,0	0,0	0,5	0,2	0,434	0,307	0,216	NA	Inf	0,0	Inf	NA
tet(36)	52,8	116,0	35,2	83,7	0,315	0,038	0,283	0,013	1,6	3,3	0,7	2,2
tet(37)	0,5	3,4	0,0	2,1	0,315	0,072	0,361	0,054	4,3	Inf	0,0	7,1
tet(38)	1,4	0,6	0,7	0,6	0,143	1,000	0,468	0,148	0,5	0,8	0,5	0,4
tet(39)	0,5	2,0	0,0	1,2	0,955	0,149	0,361	0,498	2,3	Inf	0,0	3,8
tet(40)	2154,1	3869,8	2284,6	3235,7	0,083	0,476	0,368	0,081	1,5	1,7	1,1	1,8
tet(41)	1,2	0,0	0,0	0,0	0,314	NA	0,596	0,470	0,0	NA	0,0	0,0
tet(44)	250,2	182,6	170,2	177,6	0,055	0,257	0,283	0,059	0,7	1,1	0,7	0,7
tet(A)	82,4	292,0	78,0	206,4	0,043	0,010	1,000	0,001	2,5	3,7	0,9	3,5
tet(B)	24,1	136,1	15,0	87,7	0,274	0,019	0,368	0,013	3,6	9,1	0,6	5,7

tet(C)	100,7	107,2	172,7	133,4	0,173	0,114	0,109	0,491	1,3	0,6	1,7	1,1
tet(D)	3,9	42,4	5,2	27,5	0,094	0,235	0,636	0,044	7,1	8,2	1,3	11,0
tet(E)	0,5	1,0	0,0	0,6	0,732	0,287	0,596	0,419	1,2	Inf	0,0	1,9
tet(G)	440,6	1007,1	460,1	788,3	0,122	0,171	0,683	0,059	1,8	2,2	1,0	2,3
tet(H)	99,1	12,4	60,1	31,5	0,893	0,106	0,349	0,644	0,3	0,2	0,6	0,1
tet(J)	2,8	1,8	0,0	1,1	0,452	0,540	0,361	0,788	0,4	Inf	0,0	0,6
tet(K)	1,6	0,4	0,5	0,5	0,153	1,000	0,298	0,259	0,3	0,8	0,3	0,3
tet(L)	105,8	217,4	107,4	173,4	0,274	0,171	1,000	0,108	1,6	2,0	1,0	2,1
tet(M)	6144,4	11530,0	10299,7	11037,9	0,122	1,000	0,214	0,228	1,8	1,1	1,7	1,9
tet(O)	12664,2	13688,1	13406,5	13575,5	0,515	0,914	0,808	0,491	1,1	1,0	1,1	1,1
tet(Q)	22292,8	31013,4	29197,6	30287,1	0,004	1,000	0,109	0,005	1,4	1,1	1,3	1,4
tet(S)	35,7	43,9	53,5	47,8	1,000	0,352	0,683	0,755	1,3	0,8	1,5	1,2
tet(T)	1778,1	1084,7	3386,3	2005,3	0,633	0,038	0,109	0,662	1,1	0,3	1,9	0,6
tet(U)	0,0	0,0	4,1	1,7	0,222	0,094	0,050	NA	Inf	0,0	Inf	NA
tet(W)	12109,7	12059,9	10607,4	11478,9	0,360	1,000	0,368	0,573	0,9	1,1	0,9	1,0
tet(X)	2153,2	1669,8	2150,3	1862,0	0,633	0,610	0,933	0,414	0,9	0,8	1,0	0,8
tet(Y)	85,1	74,1	69,5	72,3	0,965	1,000	1,000	0,950	0,8	1,1	0,8	0,9
tet(Z)	2,4	10,6	14,5	12,2	0,166	0,336	0,050	0,648	5,1	0,7	6,1	4,4
tetA(P)	8,9	22,1	6,0	15,7	0,248	0,171	0,683	0,196	1,8	3,7	0,7	2,5
tetB(P)	16,9	49,6	11,8	34,5	0,274	0,352	0,683	0,228	2,0	4,2	0,7	2,9
tlr(C)	1,0	1,0	1,6	1,2	0,626	0,570	0,636	0,775	1,2	0,6	1,5	0,9
VanA	0,3	0,0	0,0	0,0	0,314	NA	0,596	0,470	0,0	NA	0,0	0,0
VanAo2	0,5	0,0	0,0	0,0	0,122	NA	0,361	0,244	0,0	NA	0,0	0,0
VanB	6,9	11,6	3,4	8,3	0,762	0,038	0,283	0,181	1,2	3,4	0,5	1,7
VanC	5,0	9,6	2,2	6,7	0,623	0,165	0,261	0,948	1,3	4,3	0,5	1,9
VanD	26,4	49,3	80,1	61,6	0,006	0,762	0,048	0,020	2,3	0,6	3,0	1,9
VanE	0,2	0,0	0,0	0,0	0,314	NA	0,596	0,470	0,0	NA	0,0	0,0
VanF	0,5	0,0	0,0	0,0	0,122	NA	0,361	0,244	0,0	NA	0,0	0,0
VanG	161,0	79,3	102,3	88,5	0,083	0,476	0,461	0,059	0,6	0,8	0,6	0,5
VanM	0,2	0,0	0,5	0,2	1,000	0,307	0,602	0,470	0,8	0,0	2,1	0,0
VanN	0,2	0,0	0,0	0,0	0,314	NA	0,596	0,470	0,0	NA	0,0	0,0
VanPt	1,6	0,6	0,0	0,3	0,038	0,287	0,072	0,148	0,2	Inf	0,0	0,4
vat(B)	0,0	0,6	0,0	0,4	0,222	0,287	NA	0,112	Inf	Inf	NA	Inf
vat(E)	0,0	2,0	0,7	1,5	0,116	0,694	0,216	0,112	Inf	2,7	Inf	Inf
vga(A)	0,3	0,0	0,0	0,0	0,314	NA	0,596	0,470	0,0	NA	0,0	0,0
vga(B)	0,3	0,3	0,7	0,5	0,732	0,761	0,602	1,000	1,7	0,4	2,5	1,1
vga(C)	0,3	0,0	0,0	0,0	0,314	NA	0,596	0,470	0,0	NA	0,0	0,0
vga(E)	1,7	4,2	10,0	6,6	0,108	0,476	0,146	0,240	3,9	0,4	5,9	2,5

Supplementary Table 7: Overview of norovirus (NoV) genome copies present in 5 ml tested airplane toilet samples[a]

			Flight No	Sampling date	GI	GII	GI+GII
F015-5	South Asia	Thailand	TG 950	23-08-2013	4,76E+03	5,99E+04	6,46E+04
F016-16	South Asia	Thailand/Bankok	TG 950	27-06-2013	1,86E+03	7,49E+03	9,34E+03
F016-12	South Asia	Thailand	TG 950	29-08-2013	4,38E+06	1,23E+03	4,39E+06
F016-11	South Asia	Singapore	Singapore Airline	29-08-2013	<tLOD	1,42E+03	1,42E+03
F015-4	South Asia	Singapore	SQ 352	23-08-2013	1,17E+02	6,95E+01	1,86E+02
F015-8	South Asia	Pakistan/Islamabad	PK 771	24-08-2013	1,36E+02	2,61E+06	2,61E+06
F016-09	North Asia	China/Beijing	SK996	28-08-2013	3,26E+04	6,74E+01	3,27E+04
F015-3	North Asia	Japan	SK084	22-08-2013	3,76E+01	5,53E+01	9,29E+01
F016-10	North Asia	Japan/Tokyo	SK984	28-08-2013	2,21E+04	1,85E+01	2,22E+04
F017-1	North Asia	China	SK996	22-08-2013	<tLOD	8,08E+00	8,08E+00
F016-15	North America	Canada/toronto	AC 882	27-06-2013	<tLOD	9,80E+02	9,80E+02
F015-6	North America	USA Washington	SK 926	23-08-2013	<tLOD	1,21E+02	1,21E+02
F017-14	North America	USA Washington	SK 926	29-08-2013	1,53E+05	1,85E+02	1,53E+05
F017-13	North America	USA Newark NY	SK 910	29-08-2013	2,46E+04	4,96E+01	2,46E+04
F017-17	North America	USA Washington	SK 926	27-06-2013	<tLOD	<tLOD	<tLOD
F017-18	North America	USA Newark NY	SK 910	27-06-2013	<tLOD	<tLOD	<tLOD
F017-7	North America	USA Newark NY	SK 910	23-08-2013	<tLOD	<tLOD	<tLOD
F015-2	North America	Greenland	GL 782	22-08-2013	<tLOD	<tLOD	<tLOD
No of positive detections					10	14	14
Minimum concentration detected					3,76E+01	8,08E+00	8,08E+00
Maximun concentration detected					4,38E+06	2,61E+06	4,39E+06
Geometric Mean of positive detections					6,02E+03	5,13E+02	6,79E+03

P-values for comparison between NoV levels of different origins[d]	Asia vs North America	0,125	0,044	0,067
	South Asia vs North Asia	1	0,0095	0,257
	North Asia v North America	0,29	0,795	0,436
	South Asia v North America	0,148	0,007	0,043

[a] Estimated amounts of detected NoV genogroup (G)I and GII genome copies (CG) by real-time RT-PCR in 5 ml tested airplane toilet samples (expressed in numbers and log scale).
[b] <tLOD, not detected or below theoretical limit of detection calculated to 8 GC/ml or Log(0.90) GC/ml airplane toilet sample.
[c] tLOD, Log(0.90) GC/ml airplane toilet sample, was used for comparison of P-values for inclusion of negative detections in the T-Test for log-transformed values
[d] Statistical analysis using Wilcoxon Rank Sum Test

Performance of method applied to detect norovirus in airplane toilet samples

Extraction efficiency[a] (% of MC_0 Rec ± SD)		Amplification efficiency[b] (% ± SD))			
		norovirus GI		norovirus GII	
Undiluted RNA	1/10[c] RNA	Undiluted RNA	1/10[c] RNA	Undiluted RNA	1/10[c] RNA
19.9 ± 23.8	25.6 ± 23.9	81.4 ± 19.4	75.6 ± 23.0	86.5 ± 10.9	94.7 ± 7.7

[a] Average extraction efficiency of internal process control, mengovirus (MC_0), recovered during nucleic acid extraction after inoculation in 5 ml air-toilet samples; ((Mean RT-PCR Units recovered)/(Mean RT-PCR U inoculatede) × 100).
[b] Average amplification efficiency during detection of norovirus GI or GII genomes recovered after inoculation in dublicates of 2.5µl RNA extracts; ((Mean genome copies recovered)/(Mean genome copies inoculated) × 100).
[c] 1:10 indicates dilution of the extracted RNA prior to analysis.

Supplementary table S7: Identification of specific pathogens in airline waste

										Flight									
	China-2	China-3	Japan-2	Japan-3	Singapore-2	Singapore-3	Pakistan	Thailand-1	Thailand-2	Thailand-3	Newark-1	Newark-2	Newark-3	Washington-1	Washington-2	Washington-3	Toronto	Greenland
Number of reads mappng to MetaHitAssembly	80323500	72390218	55633018	81960564	64756198	102443028	86911454	78957422	94506024	88750766	85279992	77076030	86873034	100336712	72222900	91944290	78108170	72416918
Number of reads mapping to Bacteria	15515220	14036912	11155040	12826418	12046278	19063912	19262526	12074186	17290900	15612290	15430798	15484890	16684544	17923640	12864702	17688794	14223790	14296084
**Sum**	**95838720**	**86427130**	**66788058**	**94786982**	**76802476**	**121506940**	**106173980**	**91031608**	**111796924**	**104363056**	**100710790**	**92560920**	**103557578**	**118260352**	**85087602**	**109633084**	**92331960**	**86713002**

Pathogen unique read counts (unique reads mapping to genomes below)										Flight									
	China-2	China-3	Japan-2	Japan-3	Singapore-2	Singapore-3	Pakistan	Thailand-1	Thailand-2	Thailand-3	Newark-1	Newark-2	Newark-3	Washington-1	Washington-2	Washington-3	Toronto	Greenland
Salmonella enterica subsp. enterica serovar Typhimurium DT104, HF937208.1	952	596	388	988	836	1478	2504	716	1764	900	786	914	1198	706	550	840	654	252
Clostridium difficile R20291 complete genome, FN545816.1	32702	29126	22550	21526	22358	33444	23996	18328	30648	30794	28810	32690	40350	38278	24006	34540	26468	28488
Campylobacter jejuni subsp. jejuni IA3902, complete genome, CP001876.1	2266	2630	1186	2646	1560	3054	5024	1776	2072	3430	1844	1442	3010	4162	2888	3238	2558	1064

Normalised read count*1 mill / (MetaHit+Bacteria)

Pathogen normalized counts										Flight									
	China-2	China-3	Japan-2	Japan-3	Singapore-2	Singapore-3	Pakistan	Thailand-1	Thailand-2	Thailand-3	Newark-1	Newark-2	Newark-3	Washington-1	Washington-2	Washington-3	Toronto	Greenland
Salmonella enterica subsp. enterica serovar Typhimurium DT104, HF937208.1	9,9333547	6,89598278	5,8094218	10,423372	10,8850657	12,1639143	23,5839327	7,86539989	15,7786094	8,62374134	7,80452621	9,87457774	11,5684436	5,969879068	6,463926437	7,661920739	7,0831378	2,9061386
Clostridium difficile R20291 complete genome, FN545816.1	341,219081	337,000662	337,63521	227,0987	291,110406	275,243538	226,006409	201,336661	274,139922	295,066101	286,066667	353,172808	389,638313	323,6756813	282,1327601	315,0508837	286,6613	328,53205
Campylobacter jejuni subsp. jejuni IA3902, complete genome, CP001876.1	23,6438884	30,4302596	17,757666	27,915226	20,3118452	25,1343668	47,3185615	19,509707	18,5336047	32,8660364	18,3098554	15,578929	29,0659559	35,19353638	33,94149009	29,53488018	27,704383	12,270363

Statistical analysis using T-test based on the Normalised read counts

A_NA	SA_NA	SA_CJ	NA_CJ
0,03225247	0,03124541	0,0534578	0,2890167
0,03510631	0,00669997	0,0889201	0,3817684
0,39404908	0,35668237	0,3363526	0,475277

A: Asia; NA: North America; SA: South Asia; CJ: China and Japan.

Supplementary Table 7: Overview of norovirus (NoV) genome copies present in 5 ml tested airplane toilet samples[a]

			Flight No	Sampling date	GI	GII	GI+GII
F015-5	South Asia	Thailand	TG 950	23-08-2013	4,76E+03	5,99E+04	6,46E+04
F016-16	South Asia	Thailand/Bankok	TG 950	27-06-2013	1,86E+03	7,49E+03	9,34E+03
F016-12	South Asia	Thailand	TG 950	29-08-2013	4,38E+06	1,23E+03	4,39E+06
F016-11	South Asia	Singapore	Singapore Airline	29-08-2013	<tLOD	1,42E+03	1,42E+03
F015-4	South Asia	Singapore	SQ 352	23-08-2013	1,17E+02	6,95E+01	1,86E+02
F015-8	South Asia	Pakistan/Islamabad	PK 771	24-08-2013	1,36E+02	2,61E+06	2,61E+06
F016-09	North Asia	China/Beijing	SK996	28-08-2013	3,26E+04	6,74E+01	3,27E+04
F015-3	North Asia	Japan	SK084	22-08-2013	3,76E+01	5,53E+01	9,29E+01
F016-10	North Asia	Japan/Tokyo	SK984	28-08-2013	2,21E+04	1,85E+01	2,22E+04
F017-1	North Asia	China	SK996	22-08-2013	<tLOD	8,08E+00	8,08E+00
F016-15	North America	Canada/toronto	AC 882	27-06-2013	<tLOD	9,80E+02	9,80E+02
F015-6	North America	USA Washington	SK 926	23-08-2013	<tLOD	1,21E+02	1,21E+02
F017-14	North America	USA Washington	SK 926	29-08-2013	1,53E+05	1,85E+02	1,53E+05
F017-13	North America	USA Newark NY	SK 910	29-08-2013	2,46E+04	4,96E+01	2,46E+04
F017-17	North America	USA Washington	SK 926	27-06-2013	<tLOD	<tLOD	<tLOD
F017-18	North America	USA Newark NY	SK 910	27-06-2013	<tLOD	<tLOD	<tLOD
F017-7	North America	USA Newark NY	SK 910	23-08-2013	<tLOD	<tLOD	<tLOD
F015-2	North America	Greenland	GL 782	22-08-2013	<tLOD	<tLOD	<tLOD
No of positive detections					10	14	14
Minimum concentration detected					3,76E+01	8,08E+00	8,08E+00
Maximun concentration detected					4,38E+06	2,61E+06	4,39E+06
Geometric Mean of positive detections					6,02E+03	5,13E+02	6,79E+03
P-values for comparison between NoV levels of different origins[d]	Asia vs North America				0,125	0,044	0,067
	South Asia vs North Asia				1	0,0095	0,257
	North Asia v North America				0,29	0,795	0,436
	South Asia v North America				0,148	0,007	0,043

[a] Estimated amounts of detected NoV genogroup (G)I and GII genome copies (CG) by real-time RT-PCR in 5 ml tested airplane toilet samples (expressed in numbers and log scale).
[b] <tLOD, not detected or below theoretical limit of detection calculated to 8 GC/ml or Log(0.90) GC/ml airplane toilet sample.
[c] tLOD, Log(0.90) GC/ml airplane toilet sample, was used for comparison of P-values for inclusion of negative detections in the T-Test for log-transformed values
[d] Statistical analysis using Wilcoxon Rank Sum Test

Performance of method applied to detect norovirus in airplane toilet samples

Extraction efficiency[a] (% of MC_0 Rec ± SD)		Amplification efficiency[b] (% ± SD))			
		norovirus GI		norovirus GII	
Undiluted RNA	1/10[c] RNA	Undiluted RNA	1/10[c] RNA	Undiluted RNA	1/10[c] RNA
19.9 ± 23.8	25.6 ± 23.9	81.4 ± 19.4	75.6 ± 23.0	86.5 ± 10.9	94.7 ± 7.7

[a] Average extraction efficiency of internal process control, mengovirus (MC_0), recovered during nucleic acid extraction after inoculation in 5 ml air-toilet samples; ((Mean RT-PCR Units recovered)/(Mean RT-PCR U inoculatede) × 100).
[b] Average amplification efficiency during detection of norovirus GI or GII genomes recovered after inoculation in dublicates of 2.5µl RNA extracts; ((Mean genome copies recovered)/(Mean genome copies inoculated) × 100).
[c] 1:10 indicates dilution of the extracted RNA prior to analysis.

Supplementary table S8. Conversion table of ResFinder ide

Resfinder id	Gene name
aadA12_1_AY665771	aadA
aadA22_1_AM261837	aadA
aadA11_1_AY144590	aadA
aac(3)-Id_1_AB114632	aac(3)-I
aac(3)-IIIa_1_X55652	aac(3)-III
aadA21_1_AY171244	aadA
aadA2_1_X68227	aadA
aac(3)-If_1_AY884051	aac(3)-I
aadA23_1_AJ809407	aadA
aac(2')-Ic_1_U72714	aac(2')-I
aadA5_1_AF137361	aadA
aac(2')-Id_1_U72743	aac(2')-I
aac(2')-Ie_1_NC_011896	aac(2')-I
aadA1_5_JX185132	aadA
aac(3)-Ic_1_AJ511268	aac(3)-I
aph(2'')-Ib_1_AF337947	aph(2'')-I
aph(2'')-Ic_1_U51479	aph(2'')-I
aac(3)-Ib_1_L06157	aac(3)-I
aadA14_1_AJ884726	aadA
aadA15_1_DQ393783	aadA
aph(3')-IIc_1_AM743169	aph(3')-II
aadA13_1_AY713504	aadA
aac(2')-Ib_1_U41471	aac(2')-I
aac(2')-Ia_1_L06156	aac(2')-I
aph(3')-Ic_1_X62115	aph(3')-I
aph(3')-IV_1_X03364	aph(3')-IV
aph(3')-Ia_1_V00359	aph(3')-I
aph(3')-Ib_1_M20305	aph(3')-I
aadA24_1_AM711129	aadA
aph(3')-Id_1_Z48231	aph(3')-I
aph(3')-VIII_1_AF182845	aph(3')-VIII
aph(3')-VIIa_1_M29953	aph(3')-VII
aph(3')-Vc_1_S81599	aph(3')-V
aph(3')-XV_1_Y18050	aph(3')-XV
aph(4)-Ia_1_V01499	aph(4)-I
aadA4_1_Z50802	aadA
aph(6)-Ia_1_AY971801	aph(6)-I
aph(6)-Ib_1_X05648	aph(6)-I
aph(6)-Ic_1_X01702	aph(6)-I
aph(4)-Ib_1_X03615	aph(4)-I
aac(3)-Ig_1_CP000282	aac(3)-I
aac(3)-Ih_1_CP000490	aac(3)-I

aph(3')-VIb_1_AJ627643	aph(3')-VI
aph(9)-Ia_1_U94857	aph(9)-I
aadA3_1_AF047479	aadA
aac(3)-VIIa_1_M22999	aac(3)-VII
aac(3)-VIa_1_M88012	aac(3)-VI
aac(3)-Xa_1_AB028210	aac(3)-X
aac(6')-31_1_AJ640197	aac(6')-31
aac(6')-32_1_EF614235	aac(6')-32
aac(6')-33_1_GQ337064	aac(6')-33
aac(6')-I30_1_AY289608	aac(6')-I30
aac(6')-IIa_1_M29695	aac(6')-II
aac(6')-IIb_1_L06163	aac(6')-II
aac(6')-IIc_1_AF162771	aac(6')-II
aac(6')-Ia_1_M18967	aac(6')-I
aadA2_2_JQ364967	aadA
aac(6')-Iad_1_AB119105	aac(6')-I
aac(6')-Iae_1_AB104852	aac(6')-I
aac(6')-Iaf_1_AB462903	aac(6')-I
aac(6')-Iai_1_EU886977	aac(6')-I
aac(6')-Ib_1_M21682	aac(6')-I
aac(6')-Ic_1_M94066	aac(6')-I
aac(6')-Id_1_X12618	aac(6')-I
aac(6')-If_1_X55353	aac(6')-I
aac(6')-Ig_1_L09246	aac(6')-I
aac(6')-Ih_1_L29044	aac(6')-I
aac(6')-Ii_1_L12710	aac(6')-I
aac(6')-Ij_1_L29045	aac(6')-I
aac(6')-Iaa_1_NC_003197	aac(6')-I
aac(6')-Il_1_Z54241	aac(6')-I
aac(6')-Im_1_AF337947	aac(6')-I
aac(6')-Iq_1_AF047556	aac(6')-I
aac(6')-Ir_1_AF031326	aac(6')-I
aac(6')-Is_1_AF031327	aac(6')-I
aac(6')-It_1_AF031328	aac(6')-I
aac(6')-Iu_1_AF031329	aac(6')-I
aac(6')-Iv_1_AF031330	aac(6')-I
aac(6')-Iw_1_AF031331	aac(6')-I
aac(6')-Iy_1_AF144880	aac(6')-I
aph(9)-Ib_1_U70376	aph(9)-I
apmA_1_FN806789	apmA
aac(6')-Ik_1_L29510	aac(6')-I
aac(3)-Ii_1_CP000356	aac(3)-I
aac(3)-VIIIa_1_M55426	aac(3)-VIII
aac(6')_1_AY553333	aac(6')

aac(6')_2_DQ302723	aac(6')
aac(6')_3_EU912537	aac(6')
aacA29_1_AY139599	aacA
aacA43_1_HQ247816	aacA
aac_1_AJ628983	aac
aadA10_1_U37105	aadA
strB_4_FJ474091	strB
strB_4_NC_003384	strB
aph(2'')-Id_1_AF016483	aph(2'')-I
aph(2'')-Ie_1_AY743255	aph(2'')-I
aph(3'')-Ia_1_M16482	aph(3'')-I
aadA16_1_EU675686	aadA
aadA17_1_FJ460181	aadA
aadA1_1_X02340	aadA
aadA1_2_FJ591054	aadA
aadA1_2_JN815078	aadA
aadA1_3_JQ414041	aadA
aadA1_4_M95287	aadA
aadA1_5_JQ480156	aadA
aadA8b_1_AM040708	aadA
aadA9_1_AJ420072	aadA
aac(3)-IIIb_1_L06160	aac(3)-III
aac(3)-IIIc_1_L06161	aac(3)-III
aph(3')-VIa_1_X07753	aph(3')-VI
aac(3)-IIb_1_M97172	aac(3)-II
aph(3')-Va_1_K00432	aph(3')-V
aph(3')-Vb_1_M22126	aph(3')-V
aac(3)-IIe_1_EU022315	aac(3)-II
aac(3)-IVa_1_X01385	aac(3)-IV
aadA6_1_AF140629	aadA
aadA7_1_AF224733	aadA
aadA8_1_AF326210	aadA
ant(9)-Ib_1_M69221	ant(9)-I
aac(3)-Ib-aac(6')-Ib_1_AF355189	aac(3)-Ib-aac(6')-I
aadB_1_JN119852	aadB
aadD_1_AF181950	aadD
armA_1_AY220558	armA
strA_1_M96392	strA
strA_2_M28829	strA
ant(3'')-Ih-aac(6')-IId_1_AF453998	ant(3'')-Ih-aac(6')-IId
ant(4')-IIa_1_M98270	ant(4')-II
ant(4')-IIb_1_AY114142	ant(4')-II
ant(4')-Ib_1_AJ506108	ant(4')-I
ant(6)-Ia_1_AF330699	ant(6)-I

ant(6)-Ib_1_FN594949	ant(6)-I
aac(3)-Ia_1_X15852	aac(3)-I
strB_3_AF024602	strB
spc_1_X02588	spc
str_1_X92946	str
str_2_FN435330	str
str_3_AM932524	str
aph(3'')-Ic_1_DQ336355	aph(3'')-I
aph(3')-III_1_M26832	aph(3')-III
aph(3')-IIa_1_X57709	aph(3')-II
aph(3')-IIb_1_X90856	aph(3')-II
npmA_1_AB261016	npmA
aac(3)-IXa_1_M55427	aac(3)-IX
aac(3)-I_1_AJ877225	aac(3)-I
strB_2_M28829	strB
rmtD2_1_HQ401565	rmtD
rmtD_1_DQ914960	rmtD
spc_2_U50980	spc
sph_1_HQ424461	sph
aac(6')-aph(2'')_1_M13771	aac(6')-aph(2'')
aac(3)-IIa_1_X51534	aac(3)-II
strA_5_AF321550	strA
aac(3)-IIc_1_X54723	aac(3)-II
aac(3)-IId_1_EU022314	aac(3)-II
rmtA_1_AB120321	rmtA
strA_4_NC_003384	strA
strB_1_M96392	strB
rmtC_1_AB194779	rmtC
aac(6')-Iz_1_AF140221	aac(6')-I
rmtE_1_GU201947	rmtE
strA_3_AF024602	strA
strA_4_AF321551	strA
rmtB_1_AB103506	rmtB
POM-1_1_GU002295	POM-1
blaGOB-14_1_AY647252	blaGOB
blaGOB-15_1_AY775547	blaGOB
blaGOB-16_1_GU188443	blaGOB
blaGOB-16_2_AY899331	blaGOB
blaGOB-17_1_AY899332	blaGOB
blaGOB-18_1_DQ004496	blaGOB
blaGOB-1_1_EF394442	blaGOB
blaGOB-1_2_AF090141	blaGOB
blaGOB-2_1_AF189296	blaGOB
blaGOB-3_1_AF189291	blaGOB

blaGOB-4_1_AF189293	blaGOB
blaGOB-5_1_AF189290	blaGOB
blaGOB-6_1_AF189292	blaGOB
blaGOB-7_1_AF189297	blaGOB
blaGOB-8_1_AY348327	blaGOB
blaGOB-9_1_AY647246	blaGOB
blaHERA-1_1_AF311385	blaHERA
blaHERA-2_1_AF398334	blaHERA
blaHERA-3_1_AF398335	blaHERA
blaHERA-4_1_AJ536088	blaHERA
blaHERA-5_1_AJ536089	blaHERA
ACC-1_2_AM939420	blaACC
blaACC-2_1_AF180952	blaACC
blaACC-3_1_AF180958	blaACC
blaACC-4_1_GU256641	blaACC
blaACI-1_1_AJ007350	blaACI
blaACT-10_1_JN848330	blaACT
blaACT-12_1_JX440355	blaACT
blaACT-14_1_JX440354	blaACT
blaACT-15_1_JX440356	blaACT
blaACT-16_1_AB737978	blaACT
blaACT-1_1_U58495	blaACT
blaACT-2_1_AM076977	blaACT
blaACT-3_1_EF125013	blaACT
blaACT-4_2_AJ311172	blaACT
blaACT-5_1_FJ237369	blaACT
blaACT-6_1_FJ237366	blaACT
blaACT-7_1_FJ237368	blaACT
blaACT-9_1_HQ693810	blaACT
blaADC-25_1_EF016355	blaADC
blaAER-1_1_U14748	blaAER
blaAST-1_1_AF279904	blaAST
blaA_1_DQ424965	blaA
blaA_2_AY954728	blaA
blaB-10_1_AY348325	blaB
blaB-11_1_AY348326	blaB
blaB-12_1_EF595958	blaB
blaB-13_1_EF595959	blaB
blaB-14_1_JN635697	blaB
blaB-2_1_AF189300	blaB
blaB-3_1_AF189301	blaB
blaB-3_2_AF189299	blaB
blaB-5_1_AF189303	blaB
blaB-6_1_AF189302	blaB

blaB-7_1_AF189304	blaB
blaB-8_1_AF189305	blaB
blaB-9_1_AY348324	blaB
blaBEL-1_1_DQ089809	blaBEL
blaBEL-2_1_FJ666063	blaBEL
blaBEL-3_1_GQ202694	blaBEL
blaBES-1_1_AF234999	blaBES
blaBIC-1_1_GQ260093	blaBIC
blaBIL-1_1_X74512	blaBIL
blaCARB-10_1_EU850412	blaCARB
blaCARB-11_1_AY008290	blaCARB
blaCARB-12_1_D13210	blaCARB
blaCARB-1_1_AF313471	blaCARB
blaCARB-2_1_M69058	blaCARB
blaCARB-3_1_S46063	blaCARB
blaCARB-4_1_U14749	blaCARB
blaCARB-5_1_AF135373	blaCARB
blaCARB-6_1_AF030945	blaCARB
blaCARB-7_1_AF409092	blaCARB
blaCARB-8_1_AY178993	blaCARB
blaCARB-9_1_AY248038	blaCARB
blaCEPH-A3_1_AY112998	blaCEPH
blaCFE-1_1_AB107899	blaCFE
blaCGB-1_1_EF672680	blaCGB
blaCKO-1_1_AF477396	blaCKO
blaCME-1_1_AJ006275	blaCME
blaCMG_1_AY265892	blaCMG
blaCMY-104_1_KF150216	blaCMY
blaCMY-10_1_AF373218	blaCMY
blaCMY-110_1_AB872957	blaCMY
blaCMY-11_1_AF381626	blaCMY
blaCMY-12_1_Y16785	blaCMY
blaCMY-13_1_AY339625	blaCMY
blaCMY-14_1_EU600193	blaCMY
blaCMY-14_2_AJ555825	blaCMY
blaCMY-15_1_AJ555823	blaCMY
blaCMY-16_1_FM995219	blaCMY
blaCMY-16_2_FJ855437	blaCMY
blaCMY-17_1_AY513266	blaCMY
blaCMY-18_1_AY743434	blaCMY
blaCMY-19_1_AB194410	blaCMY
blaCMY-1_1_X92508	blaCMY
blaCMY-20_1_AY960293	blaCMY
blaCMY-21_1_DQ139328	blaCMY

blaCMY-22_1_DQ256079	blaCMY
blaCMY-23_1_DQ438952	blaCMY
blaCMY-24_1_EF415650	blaCMY
blaCMY-25_1_EU515249	blaCMY
blaCMY-26_1_AB300358	blaCMY
blaCMY-27_1_EU515250	blaCMY
blaCMY-28_1_EF561644	blaCMY
blaCMY-29_1_EF685371	blaCMY
blaCMY-2_1_X91840	blaCMY
blaCMY-30_1_EF685372	blaCMY
blaCMY-31_1_EU331425	blaCMY
blaCMY-32_1_EU496815	blaCMY
blaCMY-33_1_EU496816	blaCMY
blaCMY-34_1_EF394370	blaCMY
blaCMY-35_1_EF394371	blaCMY
blaCMY-36_1_EU331426	blaCMY
blaCMY-37_1_AB280919	blaCMY
blaCMY-38_1_AM931008	blaCMY
blaCMY-39_1_HM565135	blaCMY
blaCMY-3_1_Y16783	blaCMY
blaCMY-40_1_EU515251	blaCMY
blaCMY-41_1_AB429270	blaCMY
blaCMY-42_1_HM146927	blaCMY
blaCMY-43_1_HQ267530	blaCMY
blaCMY-44_1_FJ437066	blaCMY
blaCMY-45_1_FN546177	blaCMY
blaCMY-46_1_FN556186	blaCMY
blaCMY-47_1_HM046998	blaCMY
blaCMY-48_1_HM569226	blaCMY
blaCMY-49_1_GQ402541	blaCMY
blaCMY-4_1_AF420597	blaCMY
blaCMY-51_1_JQ733571	blaCMY
blaCMY-53_1_HQ336940	blaCMY
blaCMY-54_1_HM544039	blaCMY
blaCMY-55_1_HM544040	blaCMY
blaCMY-56_1_HQ322613	blaCMY
blaCMY-57_1_HQ285243	blaCMY
blaCMY-58_1_HQ185697	blaCMY
blaCMY-59_2_AB587082	blaCMY
blaCMY-5_1_Y17716	blaCMY
blaCMY-60_1_HQ267531	blaCMY
blaCMY-61_1_JF460795	blaCMY
blaCMY-62_1_JF460796	blaCMY
blaCMY-63_1_HQ650104	blaCMY

blaCMY-64_1_HQ832678	blaCMY
blaCMY-65_1_JF780936	blaCMY
blaCMY-66_1_JN714478	blaCMY
blaCMY-67_1_JQ711185	blaCMY
blaCMY-68_1_JN714480	blaCMY
blaCMY-69_1_JX049132	blaCMY
blaCMY-6_1_AJ011293	blaCMY
blaCMY-70_1_JX440350	blaCMY
blaCMY-71_1_JQ711184	blaCMY
blaCMY-72_1_JX440352	blaCMY
blaCMY-73_1_GQ351345	blaCMY
blaCMY-74_1_JX440349	blaCMY
blaCMY-75_1_JQ733572	blaCMY
blaCMY-76_1_JQ733573	blaCMY
blaCMY-77_1_JX440353	blaCMY
blaCMY-78_1_JQ733575	blaCMY
blaCMY-79_1_JQ733576	blaCMY
blaCMY-7_1_AY324388	blaCMY
blaCMY-80_1_JQ733577	blaCMY
blaCMY-81_1_JQ733578	blaCMY
blaCMY-83_1_JX440351	blaCMY
blaCMY-84_1_JQ733579	blaCMY
blaCMY-87_1_AB699171	blaCMY
blaCMY-8_1_EF382672	blaCMY
blaCMY-8_2_DQ094251	blaCMY
blaCMY-94_1_JX514368	blaCMY
blaCMY-95_1_JX514369	blaCMY
blaCMY-98_1_KC603538	blaCMY
blaCMY-99_1_KF305673	blaCMY
blaCMY-9_1_AB061794	blaCMY
blaCTX-M-100_1_FR682582	blaCTX-M
blaCTX-M-101_1_HQ398214	blaCTX-M
blaCTX-M-102_1_HQ398215	blaCTX-M
blaCTX-M-103_1_HG423149	blaCTX-M
blaCTX-M-104_1_HQ833652	blaCTX-M
blaCTX-M-105_1_HQ833651	blaCTX-M
blaCTX-M-106_1_HQ913565	blaCTX-M
blaCTX-M-10_1_AY598759	blaCTX-M
blaCTX-M-110_1_JF274242	blaCTX-M
blaCTX-M-111_1_JF274243	blaCTX-M
blaCTX-M-112_1_JF274246	blaCTX-M
blaCTX-M-113_1_JF274247	blaCTX-M
blaCTX-M-114_1_GQ351346	blaCTX-M
blaCTX-M-116_1_JF966749	blaCTX-M

blaCTX-M-117_1_JN227085	blaCTX-M
blaCTX-M-11_1_AJ310929	blaCTX-M
blaCTX-M-121_1_JN790862	blaCTX-M
blaCTX-M-122_1_JN790863	blaCTX-M
blaCTX-M-123_1_JN790864	blaCTX-M
blaCTX-M-124_1_JQ429324	blaCTX-M
blaCTX-M-125_1_JQ724542	blaCTX-M
blaCTX-M-126_1_AB703103	blaCTX-M
blaCTX-M-129_1_JX017364	blaCTX-M
blaCTX-M-12_1_DQ821704	blaCTX-M
blaCTX-M-130_1_JX017365	blaCTX-M
blaCTX-M-131_1_JN969893	blaCTX-M
blaCTX-M-132_1_JX313020	blaCTX-M
blaCTX-M-134_1_JX896165	blaCTX-M
blaCTX-M-136_1_KC351754	blaCTX-M
blaCTX-M-139_1_KC107824	blaCTX-M
blaCTX-M-13_2_AF252623	blaCTX-M
blaCTX-M-142_1_KF240809	blaCTX-M
blaCTX-M-147_1_KF513180	blaCTX-M
blaCTX-M-14_4_EU274579	blaCTX-M
blaCTX-M-14_5_AJ416341	blaCTX-M
blaCTX-M-15_23_DQ302097	blaCTX-M
blaCTX-M-15_64_FJ815288	blaCTX-M
blaCTX-M-15_70_FJ815277	blaCTX-M
blaCTX-M-16_1_AY029068	blaCTX-M
blaCTX-M-17_1_AY033516	blaCTX-M
blaCTX-M-17_2_AF454633	blaCTX-M
blaCTX-M-18_1_AF325133	blaCTX-M
blaCTX-M-19_1_AF325134	blaCTX-M
blaCTX-M-1_4_AJ416342	blaCTX-M
blaCTX-M-1_6_DQ915955	blaCTX-M
blaCTX-M-20_1_AJ416344	blaCTX-M
blaCTX-M-21_1_AJ416346	blaCTX-M
blaCTX-M-22_1_HM470254	blaCTX-M
blaCTX-M-22_3_EU376964	blaCTX-M
blaCTX-M-23_1_AF488377	blaCTX-M
blaCTX-M-24_1_EU921824	blaCTX-M
blaCTX-M-24_2_EF570050	blaCTX-M
blaCTX-M-24_8_EF374096	blaCTX-M
blaCTX-M-25_1_AF518567	blaCTX-M
blaCTX-M-26_1_AY455830	blaCTX-M
blaCTX-M-27_1_EU916273	blaCTX-M
blaCTX-M-28_6_AJ549244	blaCTX-M
blaCTX-M-29_1_AY267213	blaCTX-M

blaCTX-M-2_1_EU622041	blaCTX-M
blaCTX-M-30_1_AY292654	blaCTX-M
blaCTX-M-31_1_AJ567481	blaCTX-M
blaCTX-M-32_2_AM420303	blaCTX-M
blaCTX-M-33_1_AY238472	blaCTX-M
blaCTX-M-34_1_AY515297	blaCTX-M
blaCTX-M-35_1_AB176534	blaCTX-M
blaCTX-M-36_1_AB177384	blaCTX-M
blaCTX-M-37_2_FN813246	blaCTX-M
blaCTX-M-38_1_AY753197	blaCTX-M
blaCTX-M-39_1_AY954516	blaCTX-M
blaCTX-M-3_2_EF437434	blaCTX-M
blaCTX-M-3_8_EF382672	blaCTX-M
blaCTX-M-40_1_AY750914	blaCTX-M
blaCTX-M-41_1_DQ023162	blaCTX-M
blaCTX-M-42_1_DQ061159	blaCTX-M
blaCTX-M-43_1_DQ102702	blaCTX-M
blaCTX-M-44_1_D37830	blaCTX-M
blaCTX-M-45_1_D89862	blaCTX-M
blaCTX-M-46_1_AY847147	blaCTX-M
blaCTX-M-47_1_AY847143	blaCTX-M
blaCTX-M-48_1_AY847144	blaCTX-M
blaCTX-M-49_1_AY847145	blaCTX-M
blaCTX-M-4_1_Y14156	blaCTX-M
blaCTX-M-50_1_AY847146	blaCTX-M
blaCTX-M-51_1_DQ211987	blaCTX-M
blaCTX-M-52_1_GU125667	blaCTX-M
blaCTX-M-53_1_DQ268764	blaCTX-M
blaCTX-M-54_1_DQ303459	blaCTX-M
blaCTX-M-55_2_GQ456159	blaCTX-M
blaCTX-M-56_1_EF374097	blaCTX-M
blaCTX-M-58_1_EF210159	blaCTX-M
blaCTX-M-59_1_EU622856	blaCTX-M
blaCTX-M-5_6_AF286192	blaCTX-M
blaCTX-M-5_8_AJ005045	blaCTX-M
blaCTX-M-60_1_AM411407	blaCTX-M
blaCTX-M-61_1_EF219142	blaCTX-M
blaCTX-M-62_1_EF219134	blaCTX-M
blaCTX-M-63_1_EU660216	blaCTX-M
blaCTX-M-64_1_GQ456156	blaCTX-M
blaCTX-M-65_11_FJ907380	blaCTX-M
blaCTX-M-65_2_GQ456158	blaCTX-M
blaCTX-M-66_1_EF576988	blaCTX-M
blaCTX-M-67_1_EF581888	blaCTX-M

blaCTX-M-68_1_EU177100	blaCTX-M
blaCTX-M-69_1_EU402393	blaCTX-M
blaCTX-M-6_1_AJ005044	blaCTX-M
blaCTX-M-71_1_FJ815436	blaCTX-M
blaCTX-M-72_1_AY847148	blaCTX-M
blaCTX-M-74_1_GQ149243	blaCTX-M
blaCTX-M-75_1_GQ149244	blaCTX-M
blaCTX-M-76_1_AM982520	blaCTX-M
blaCTX-M-77_1_AM982521	blaCTX-M
blaCTX-M-78_1_AM982522	blaCTX-M
blaCTX-M-79_1_GU125666	blaCTX-M
blaCTX-M-80_1_EU202673	blaCTX-M
blaCTX-M-81_1_EU136031	blaCTX-M
blaCTX-M-82_1_DQ256091	blaCTX-M
blaCTX-M-83_1_FJ214366	blaCTX-M
blaCTX-M-84_1_FJ214367	blaCTX-M
blaCTX-M-85_1_FJ214368	blaCTX-M
blaCTX-M-86_1_FJ214369	blaCTX-M
blaCTX-M-87_1_EU545409	blaCTX-M
blaCTX-M-88_1_FJ873739	blaCTX-M
blaCTX-M-89_1_FJ966096	blaCTX-M
blaCTX-M-89_2_FJ971899	blaCTX-M
blaCTX-M-8_1_AF189721	blaCTX-M
blaCTX-M-90_1_FJ907381	blaCTX-M
blaCTX-M-91_1_GQ870432	blaCTX-M
blaCTX-M-92_1_GU127598	blaCTX-M
blaCTX-M-93_1_HQ166709	blaCTX-M
blaCTX-M-94_1_HM167760	blaCTX-M
blaCTX-M-95_1_FN813245	blaCTX-M
blaCTX-M-96_1_AJ704396	blaCTX-M
blaCTX-M-97_1_HM776707	blaCTX-M
blaCTX-M-98_1_HM755448	blaCTX-M
blaCTX-M-99_1_HM803271	blaCTX-M
blaCTX-M-9_1_AF174129	blaCTX-M
blaCTX-M-9_7_AJ416345	blaCTX-M
blaDES-1_1_AF426161	blaDES
blaDHA-1_1_Y16410	blaDHA
blaDHA-2_1_AF259520	blaDHA
blaDHA-3_1_AY494945	blaDHA
blaDHA-5_1_JF273491	blaDHA
blaDHA-6_1_HQ322612	blaDHA
blaDHA-7_1_HQ456945	blaDHA
blaDIM-1_1_GU323019	blaDIM
blaEBR-1_1_AF416700	blaEBR

blaERP-1_1_AY077733	blaERP
blaFAR-1_1_AF024601	blaFAR
blaFONA-1_1_AJ251239	blaFONA
blaFONA-2_1_AJ251240	blaFONA
blaFONA-3_1_AJ251241	blaFONA
blaFONA-4_1_AJ251242	blaFONA
blaFONA-5_1_AJ251243	blaFONA
blaFONA-6_1_AJ251244	blaFONA
blaFOX-10_1_JX049131	blaFOX
blaFOX-1_1_X77455	blaFOX
blaFOX-2_1_Y10282	blaFOX
blaFOX-3_1_Y11068	blaFOX
blaFOX-4_1_AJ277535	blaFOX
blaFOX-5_1_AY007369	blaFOX
blaFOX-6_1_AY034848	blaFOX
blaFOX-7_1_AJ703796	blaFOX
blaFOX-8_1_HM565917	blaFOX
blaFOX-9_1_JF896803	blaFOX
blaGES-10_1_FJ820124	blaGES
blaGES-11_1_FJ854362	blaGES
blaGES-12_1_FN554543	blaGES
blaGES-13_1_GU169702	blaGES
blaGES-14_1_GU207844	blaGES
blaGES-15_1_GU208678	blaGES
blaGES-16_1_HM173356	blaGES
ampH_1_AJ276031	ampH
ampH_2_HQ586946	ampH
ampS_1_X80276	ampS
blaGES-1_1_HQ170511	blaGES
blaGES-20_1_JN596280	blaGES
blaGES-21_1_JQ772478	blaGES
blaGES-22_1_JX023441	blaGES
blaGES-23_1_KF179354	blaGES
blaGES-2_1_AF326355	blaGES
blaGES-3_1_AB113580	blaGES
blaGES-4_1_AB116723	blaGES
blaGES-5_1_DQ236171	blaGES
blaGES-6_1_AY494718	blaGES
blaGES-7_1_HM453325	blaGES
blaGES-8_1_AF329699	blaGES
blaGES-9_1_AY920928	blaGES
blaGIM-1_1_JF414726	blaGIM
blaGOB-10_1_AY647247	blaGOB
blaGOB-11_1_AY647248	blaGOB

blaGOB-12_1_AY647249	blaGOB
blaGOB-13_1_AY647251	blaGOB
blaOXA-326_1_KF203100	blaOXA
blaOXA-327_1_KF203101	blaOXA
blaOXA-328_1_KF203102	blaOXA
blaOXA-329_1_KF203103	blaOXA
blaOXA-32_1_AF315351	blaOXA
blaOXA-330_1_KF203104	blaOXA
blaOXA-331_1_KF203105	blaOXA
blaOXA-332_1_KF203106	blaOXA
blaOXA-333_1_KF203107	blaOXA
blaOXA-334_1_KF203108	blaOXA
blaOXA-335_1_KF203109	blaOXA
blaOXA-33_1_AY008291	blaOXA
blaOXA-347_1_JN086160	blaOXA
blaOXA-348_1_KF297577	blaOXA
blaOXA-349_1_KF297578	blaOXA
blaOXA-34_1_AF350424	blaOXA
blaOXA-350_1_KF297579	blaOXA
blaOXA-351_1_KF297580	blaOXA
blaOXA-352_1_KF297581	blaOXA
blaOXA-353_1_KF297582	blaOXA
blaOXA-354_1_KF297583	blaOXA
blaHERA-6_1_AJ536090	blaHERA
blaHERA-8_1_AJ536092	blaHERA
blaIMI-1_1_U50278	blaIMI
blaIMI-2_1_DQ173429	blaIMI
blaIMI-3_1_GU015024	blaIMI
blaIMP-10_1_AB195637	blaIMP
blaIMP-11_1_AB074436	blaIMP
blaIMP-12_3_AJ420864	blaIMP
blaIMP-13_1_AJ512502	blaIMP
blaIMP-14_1_AY553332	blaIMP
blaIMP-15_1_EF184216	blaIMP
blaIMP-16_1_AJ584652	blaIMP
blaIMP-18_1_EF184215	blaIMP
blaIMP-19_1_EF118171	blaIMP
blaIMP-1_1_DQ522237	blaIMP
blaIMP-20_1_AB196988	blaIMP
blaIMP-21_1_AB204557	blaIMP
blaIMP-22_2_DQ361087	blaIMP
blaIMP-24_1_EF192154	blaIMP
blaIMP-25_1_HM175876	blaIMP
blaIMP-26_1_GU045307	blaIMP

blaIMP-27_1_JF894248	blaIMP
blaIMP-28_1_JQ407409	blaIMP
blaIMP-29_1_HQ438058	blaIMP
blaIMP-2_1_AJ243491	blaIMP
blaIMP-30_1_DQ522237	blaIMP
blaIMP-31_1_KF148593	blaIMP
blaIMP-32_1_JQ002629	blaIMP
blaIMP-33_1_JN848782	blaIMP
blaIMP-34_1_AB715422	blaIMP
blaIMP-35_1_JF816544	blaIMP
blaIMP-37_1_JX131372	blaIMP
blaIMP-38_1_HQ875573	blaIMP
blaIMP-3_1_AB010417	blaIMP
blaIMP-40_1_AB753457	blaIMP
blaIMP-41_1_AB753458	blaIMP
blaIMP-42_1_AB753456	blaIMP
blaIMP-43_1_AB777500	blaIMP
blaIMP-44_1_AB777501	blaIMP
blaIMP-4_1_DQ307573	blaIMP
blaIMP-5_1_JF810083	blaIMP
blaIMP-6_1_AB188812	blaIMP
blaIMP-7_1_AF318077	blaIMP
blaIMP-8_1_EU042136	blaIMP
blaIMP-9_1_AY033653	blaIMP
blaIND-10_1_GU206353	blaIND
blaIND-11_1_HM245379	blaIND
blaIND-12_1_HM245380	blaIND
blaIND-14_1_HM367709	blaIND
blaIND-15_1_AB563173	blaIND
blaIND-1_1_AF099139	blaIND
blaIND-2_2_AF219127	blaIND
blaIND-2a_4_AF219130	blaIND
blaIND-2b_3_EF672681	blaIND
blaIND-2c_1_GU186042	blaIND
blaIND-3_1_AF219131	blaIND
blaIND-4_1_AF219135	blaIND
blaIND-5_1_AY504627	blaIND
blaIND-6_1_AM087455	blaIND
blaIND-7_1_AB529520	blaIND
blaIND-8_1_GU186044	blaIND
blaIND-9_1_GU186045	blaIND
blaJOHN-1_1_AY028464	blaJOHN
blaKHM-1_1_AB364006	blaKHM
blaKPC-10_1_GQ140348	blaKPC

blaKPC-11_1_HM066995	blaKPC
blaKPC-12_1_HQ342889	blaKPC
blaKPC-13_1_HQ342890	blaKPC
blaKPC-14_1_JX524191	blaKPC
blaKPC-15_1_KC433553	blaKPC
blaKPC-16_1_KC465199	blaKPC
blaKPC-1_1_AF297554	blaKPC
blaKPC-2_1_AY034847	blaKPC
blaKPC-3_1_HM769262	blaKPC
blaKPC-4_1_AY700571	blaKPC
blaKPC-4_1_FJ473382	blaKPC
blaKPC-5_1_EU400222	blaKPC
blaKPC-6_1_EU555534	blaKPC
blaKPC-7_1_EU729727	blaKPC
blaKPC-8_1_FJ234412	blaKPC
blaKPC-9_1_FJ624872	blaKPC
blaL1_3_EF126059	blaL
blaLAT-1_1_X78117	blaLAT
blaLCR-1_1_X56809	blaLCR
blaLEN10_1_AJ635419	blaLEN
blaLEN11_1_AJ635417	blaLEN
blaLEN12_1_AJ635406	blaLEN
blaLEN13_1_AJ635403	blaLEN
blaLEN15_1_AF452105	blaLEN
blaLEN16_1_AY743416	blaLEN
blaLEN17_1_EF205593	blaLEN
blaLEN18_1_AM850908	blaLEN
blaLEN19_1_AM850909	blaLEN
blaLEN1_1_X04515	blaLEN
blaLEN20_1_AM850910	blaLEN
blaLEN21_1_AM850911	blaLEN
blaLEN22_1_AM850912	blaLEN
blaLEN23_1_AM850913	blaLEN
blaLEN24_1_AM850914	blaLEN
blaLEN25_1_HQ709169	blaLEN
blaLEN26_1_JQ067123	blaLEN
blaLEN2_1_AY037780	blaLEN
blaLEN3_1_AY130286	blaLEN
blaLEN4_1_AY130287	blaLEN
blaLEN5_1_AY633109	blaLEN
blaLEN7_1_AJ635425	blaLEN
blaLEN8_1_AJ635424	blaLEN
blaLEN9_1_AJ635405	blaLEN
blaLUT-1_1_AY695112	blaLUT

blaMAL-1_1_AJ277209	blaMAL
blaMAL-1_2_AJ609506	blaMAL
blaMIR-1_1_M37839	blaMIR
blaMIR-2_1_AY227752	blaMIR
blaMIR-3_1_AY743435	blaMIR
blaMIR-4_1_EF417572	blaMIR
blaMIR-5_1_FJ237367	blaMIR
blaMIR-6_1_JQ664733	blaMIR
blaMOR-2_1_AY235804	blaMOR
blaMOR_1_Y10283	blaMOR
blaMOX-1_1_D13304	blaMOX
blaMOX-2_1_AJ276453	blaMOX
blaMOX-3_1_EU515248	blaMOX
blaMOX-4_1_FJ262599	blaMOX
blaMOX-5_1_GQ152600	blaMOX
blaMOX-6_1_GQ152601	blaMOX
blaMOX-7_1_GQ152602	blaMOX
blaMUS-1_1_AF441286	blaMUS
blaNDM-1_1_FN396876	blaNDM
blaNDM-2_1_JF703135	blaNDM
blaNDM-3_1_JQ734687	blaNDM
blaNDM-4_1_JQ348841	blaNDM
blaNDM-5_1_JN104597	blaNDM
blaNDM-6_1_JN967644	blaNDM
blaNDM-7_1_JX262694	blaNDM
blaNDM-8_1_AB744718	blaNDM
blaNDM-9_1_KC999080	blaNDM
blaNMC-A_1_AJ536087	blaNMC-A
blaNPS_1_AY027589	blaNPS
blaOCH-2_1_AJ295340	blaOCH
blaOCH-3_1_AJ295341	blaOCH
blaOCH-4_1_AJ295342	blaOCH
blaOCH-5_1_AJ295343	blaOCH
blaOCH-6_1_AJ295344	blaOCH
blaOCH-7_1_AJ295345	blaOCH
blaOCH-8_1_DQ489307	blaOCH
blaOKP-A-10_1_AM051149	blaOKP-A
blaOKP-A-11_1_AM850915	blaOKP-A
blaOKP-A-12_1_AM850915	blaOKP-A
blaOKP-A-13_1_FJ534513	blaOKP-A
blaOKP-A-14_1_FJ534512	blaOKP-A
blaOKP-A-15_1_FJ755841	blaOKP-A
blaOKP-A-16_1_FJ755840	blaOKP-A
blaOKP-A-1_1_AM051138	blaOKP-A

blaOKP-A-2_1_AM051139	blaOKP-A
blaOKP-A-3_1_AM051140	blaOKP-A
blaOKP-A-4_1_AM051142	blaOKP-A
blaOKP-A-5_1_AM051143	blaOKP-A
blaOKP-A-6_1_AM051144	blaOKP-A
blaOKP-A-7_1_AM051145	blaOKP-A
blaOKP-A-8_1_AM051147	blaOKP-A
blaOKP-A-9_1_AM051148	blaOKP-A
blaOKP-B-10_1_AM051160	blaOKP-B
blaOKP-B-11_1_AM051161	blaOKP-B
blaOKP-B-12_1_AJ635420	blaOKP-B
blaOKP-B-13_1_AY825330	blaOKP-B
blaOKP-B-14_1_DQ995288	blaOKP-B
blaOKP-B-15_1_AM850917	blaOKP-B
blaOKP-B-16_1_AM850918	blaOKP-B
blaOKP-B-17_1_AM850919	blaOKP-B
blaOKP-B-18_1_AM850920	blaOKP-B
blaOKP-B-19_1_AM850921	blaOKP-B
blaOKP-B-1_1_AM051150	blaOKP-B
blaOKP-B-20_1_AM850922	blaOKP-B
blaOKP-B-2_1_AM051151	blaOKP-B
blaOKP-B-3_1_AM051152	blaOKP-B
blaOKP-B-4_1_AM051153	blaOKP-B
blaOKP-B-5_1_AY512506	blaOKP-B
blaOKP-B-6_1_AY850171	blaOKP-B
blaOKP-B-7_1_AM051156	blaOKP-B
blaOKP-B-8_1_AM051157	blaOKP-B
blaOKP-B-9_1_AM051159	blaOKP-B
blaOXA-100_1_AM231720	blaOXA
blaOXA-101_1_AM412777	blaOXA
blaOXA-104_1_EF581285	blaOXA
blaOXA-106_1_EF650032	blaOXA
blaOXA-107_1_EF650033	blaOXA
blaOXA-108_1_EF650034	blaOXA
blaOXA-109_1_EF650035	blaOXA
blaOXA-10_1_HM175875	blaOXA
blaOXA-10_2_EU886981	blaOXA
blaOXA-110_1_EF650036	blaOXA
blaOXA-111_1_EF650037	blaOXA
blaOXA-112_1_EF650038	blaOXA
blaOXA-113_1_EF653400	blaOXA
blaOXA-114_1_HM368376	blaOXA
blaOXA-114_2_HM368375	blaOXA
blaOXA-114_3_HM104634	blaOXA

blaOXA-114_4_HM104633	blaOXA
blaOXA-114_5_HM056041	blaOXA
blaOXA-114_6_HM056040	blaOXA
blaOXA-115_1_EU029998	blaOXA
blaOXA-116_1_EU220744	blaOXA
blaOXA-117_1_GQ423625	blaOXA
blaOXA-117_2_EU220745	blaOXA
blaOXA-118_1_AF371964	blaOXA
blaOXA-119_1_AY139598	blaOXA
blaOXA-11_1_Z22590	blaOXA
blaOXA-120_1_HE963768	blaOXA
blaOXA-128_1_EU375515	blaOXA
blaOXA-129_1_AM932669	blaOXA
blaOXA-12_1_U10251	blaOXA
blaOXA-130_1_EU547445	blaOXA
blaOXA-131_1_EU547446	blaOXA
blaOXA-132_1_EU547447	blaOXA
blaOXA-133_1_EU571228	blaOXA
blaOXA-134_1_FJ195387	blaOXA
blaOXA-134_2_HQ122933	blaOXA
blaOXA-136_1_EU086833	blaOXA
blaOXA-136_2_EU086832	blaOXA
blaOXA-137_1_EU086836	blaOXA
blaOXA-138_1_EU670845	blaOXA
blaOXA-139_1_AM991978	blaOXA
blaOXA-13_1_AF043558	blaOXA
blaOXA-13_1_U59183	blaOXA
blaOXA-141_1_EF552405	blaOXA
blaOXA-142_1_EU358785	blaOXA
blaOXA-143_1_GQ861437	blaOXA
blaOXA-144_1_FJ617207	blaOXA
blaOXA-144_2_FJ872530	blaOXA
blaOXA-145_1_FJ790516	blaOXA
blaOXA-146_1_FJ194460	blaOXA
blaOXA-147_1_FJ848783	blaOXA
blaOXA-148_1_GQ853679	blaOXA
blaOXA-149_1_GQ853680	blaOXA
blaOXA-14_1_L38523	blaOXA
blaOXA-150_1_GQ853681	blaOXA
blaOXA-15_1_U63835	blaOXA
blaOXA-160_1_GU199038	blaOXA
blaOXA-161_1_GQ202693	blaOXA
blaOXA-162_1_GU197550	blaOXA
blaOXA-163_1_HQ700343	blaOXA

blaOXA-164_1_GU831575	blaOXA
blaOXA-165_1_HM488986	blaOXA
blaOXA-166_1_HM488987	blaOXA
blaOXA-167_1_HM488988	blaOXA
blaOXA-168_1_HM488989	blaOXA
blaOXA-169_1_HM488990	blaOXA
blaOXA-16_1_AF043100	blaOXA
blaOXA-170_1_HM488991	blaOXA
blaOXA-171_1_HM488992	blaOXA
blaOXA-172_1_HM113558	blaOXA
blaOXA-173_1_HM113559	blaOXA
blaOXA-174_1_HM113560	blaOXA
blaOXA-175_1_HM113561	blaOXA
blaOXA-176_1_HM113562	blaOXA
blaOXA-177_1_HM113563	blaOXA
blaOXA-178_1_HM113564	blaOXA
blaOXA-179_1_HM570035	blaOXA
blaOXA-17_1_AF060206	blaOXA
blaOXA-17_2_DQ902344	blaOXA
blaOXA-180_1_HM570036	blaOXA
blaOXA-181_1_HM992946	blaOXA
blaOXA-182_1_HM640278	blaOXA
blaOXA-183_1_HQ111474	blaOXA
blaOXA-18_1_EU503121	blaOXA
blaOXA-192_1_JF273470	blaOXA
blaOXA-194_1_HQ425492	blaOXA
blaOXA-195_1_HQ425493	blaOXA
blaOXA-196_1_HQ425494	blaOXA
blaOXA-197_1_HQ425495	blaOXA
blaOXA-198_1_HQ634775	blaOXA
blaOXA-199_1_HQ637466	blaOXA
blaOXA-19_1_AF043381	blaOXA
blaOXA-1_1_J02967	blaOXA
blaOXA-200_1_HQ734811	blaOXA
blaOXA-201_1_HQ734812	blaOXA
blaOXA-202_1_HQ734813	blaOXA
blaOXA-203_1_HQ998857	blaOXA
blaOXA-204_1_JQ809466	blaOXA
blaOXA-205_1_JF800667	blaOXA
blaOXA-206_1_AB634250	blaOXA
blaOXA-207_1_JQ838185	blaOXA
blaOXA-208_1_FR853176	blaOXA
blaOXA-209_1_JF268688	blaOXA
blaOXA-20_1_AF024602	blaOXA

blaOXA-210_1_JF795487	blaOXA
blaOXA-211_1_JN861779	blaOXA
blaOXA-212_1_JN861780	blaOXA
blaOXA-213_1_JN861781	blaOXA
blaOXA-214_1_JN861782	blaOXA
blaOXA-215_1_JN861783	blaOXA
blaOXA-216_1_FR865168	blaOXA
blaOXA-217_1_JN603240	blaOXA
blaOXA-219_1_JN215211	blaOXA
blaOXA-21_2_DQ993182	blaOXA
blaOXA-223_1_JN248564	blaOXA
blaOXA-224_1_JN596991	blaOXA
blaOXA-225_1_JN638887	blaOXA
blaOXA-228_1_JQ422053	blaOXA
blaOXA-229_1_JQ422052	blaOXA
blaOXA-22_1_AF064820	blaOXA
blaOXA-230_1_JQ422054	blaOXA
blaOXA-231_1_JQ326200	blaOXA
blaOXA-232_1_JX423831	blaOXA
blaOXA-235_1_JQ820240	blaOXA
blaOXA-236_1_JQ820242	blaOXA
blaOXA-237_1_JQ820241	blaOXA
blaOXA-239_1_JQ837239	blaOXA
blaOXA-23_1_HQ700358	blaOXA
blaOXA-240_1_JX089628	blaOXA
blaOXA-241_1_JX025021	blaOXA
blaOXA-242_1_JX025022	blaOXA
blaOXA-243_1_JX206446	blaOXA
blaOXA-244_1_JX438000	blaOXA
blaOXA-245_1_JX438001	blaOXA
blaOXA-247_1_JX893517	blaOXA
blaOXA-248_1_HE963769	blaOXA
blaOXA-249_1_HE963770	blaOXA
blaOXA-24_1_AJ239129	blaOXA
blaOXA-250_1_HE963771	blaOXA
blaOXA-251_1_JN118546	blaOXA
blaOXA-253_1_KC479324	blaOXA
blaGES-17_1_HQ874631	blaGES
blaGES-18_1_JQ028729	blaGES
blaGES-19_1_JN596280	blaGES
blaOXA-257_1_KC567681	blaOXA
blaOXA-258_1_HE614014	blaOXA
blaOXA-25_1_AF201826	blaOXA
blaOXA-26_1_AF201827	blaOXA

blaOXA-278_1_KC771279	blaOXA
blaOXA-27_1_AF201828	blaOXA
blaOXA-28_1_FJ374756	blaOXA
blaOXA-29_1_AJ400619	blaOXA
blaOXA-2_1_DQ310703	blaOXA
blaOXA-2_1_GQ466184	blaOXA
blaOXA-2_2_AY444814	blaOXA
blaOXA-309_1_HF947514	blaOXA
blaOXA-31_1_AF294653	blaOXA
blaOXA-320_1_KF151169	blaOXA
blaOXA-322_1_KF203096	blaOXA
blaOXA-323_1_KF203097	blaOXA
blaOXA-324_1_KF203098	blaOXA
blaOXA-325_1_KF203099	blaOXA
blaTEM-121_1_AY307374	blaTEM
blaTEM-122_1_AY307100	blaTEM
blaTEM-123_1_AY327539	blaTEM
blaTEM-124_1_AY327540	blaTEM
blaTEM-125_1_AY628176	blaTEM
blaTEM-126_1_AY628199	blaTEM
blaTEM-127_1_AY368236	blaTEM
blaTEM-128_1_AY359287	blaTEM
blaTEM-129_1_AY452662	blaTEM
blaTEM-12_1_M88143	blaTEM
blaTEM-130_1_AJ866988	blaTEM
blaTEM-131_1_AY436361	blaTEM
blaTEM-132_1_AY491682	blaTEM
blaTEM-133_1_AY528425	blaTEM
blaTEM-134_1_AY574271	blaTEM
blaTEM-135_1_GQ896333	blaTEM
blaTEM-136_1_AY826417	blaTEM
blaTEM-137_1_AM286274	blaTEM
blaTEM-138_1_AY853593	blaTEM
blaTEM-139_1_DQ072853	blaTEM
blaTEM-141_1_AY956335	blaTEM
blaOXA-355_1_KF297584	blaOXA
blaOXA-356_1_KF297585	blaOXA
blaOXA-357_1_KF421160	blaOXA
blaOXA-358_1_KF421161	blaOXA
blaOXA-359_1_KF421162	blaOXA
blaOXA-35_1_AF315786	blaOXA
blaOXA-360_1_KF421163	blaOXA
blaOXA-36_1_AF300985	blaOXA
blaOXA-371_1_AB871653	blaOXA

blaOXA-37_1_AY007784	blaOXA
blaOXA-3_1_L07945	blaOXA
blaOXA-40_1_AF509241	blaOXA
blaOXA-42_1_AJ488302	blaOXA
blaOXA-43_1_AJ488303	blaOXA
blaOXA-45_1_AJ519683	blaOXA
blaOXA-46_1_AJ969237	blaOXA
blaOXA-47_1_AY237830	blaOXA
blaOXA-48_1_HM755942	blaOXA
blaOXA-48_2_AY236073	blaOXA
blaOXA-49_1_AY288523	blaOXA
blaOXA-4_1_AY162283	blaOXA
blaOXA-50_1_AY306135	blaOXA
blaOXA-50_2_AY306133	blaOXA
blaOXA-50_3_AY306130	blaOXA
blaOXA-50_3_AY306132	blaOXA
blaOXA-51_10_EU255289	blaOXA
blaOXA-51_11_EU255288	blaOXA
blaOXA-51_1_AJ309734	blaOXA
blaOXA-51_3_EU255296	blaOXA
blaOXA-51_4_EU255295	blaOXA
blaOXA-51_5_EU255294	blaOXA
blaOXA-51_6_EU255293	blaOXA
blaOXA-51_7_EU255292	blaOXA
blaOXA-51_8_EU255291	blaOXA
blaOXA-51_9_EU255290	blaOXA
blaOXA-53_1_AY289608	blaOXA
blaOXA-54_1_AY500137	blaOXA
blaOXA-55_1_AY343493	blaOXA
blaOXA-56_1_AY660529	blaOXA
blaOXA-56_1_EF437948	blaOXA
blaOXA-57_1_AJ631966	blaOXA
blaOXA-58_14_EU107364	blaOXA
blaOXA-58_1_AY665723	blaOXA
blaOXA-59_1_AJ632249	blaOXA
blaOXA-5_1_X58272	blaOXA
blaOXA-60_1_AY664506	blaOXA
blaOXA-60_2_AY664505	blaOXA
blaOXA-60_3_AY664504	blaOXA
blaOXA-60_4_AY662675	blaOXA
blaOXA-61_1_AY587956	blaOXA
blaOXA-62_1_AY423074	blaOXA
blaOXA-63_1_AY619003	blaOXA
blaOXA-64_1_AY750907	blaOXA

blaOXA-65_1_AY750908	blaOXA
blaOXA-66_4_FJ360530	blaOXA
blaOXA-67_1_DQ491200	blaOXA
blaOXA-68_1_AY750910	blaOXA
blaOXA-69_1_HM564339	blaOXA
blaOXA-70_1_AY750912	blaOXA
blaOXA-71_1_AY859528	blaOXA
blaOXA-72_1_GU199039	blaOXA
blaOXA-73_1_AY762325	blaOXA
blaOXA-74_1_EU161636	blaOXA
blaOXA-75_1_AY859529	blaOXA
blaOXA-76_1_AY949203	blaOXA
blaOXA-77_1_AY949202	blaOXA
blaOXA-78_1_AY862132	blaOXA
blaOXA-79_1_EU019534	blaOXA
blaOXA-7_1_X75562	blaOXA
blaOXA-80_1_EU019535	blaOXA
blaOXA-82_1_GQ352402	blaOXA
blaOXA-82_2_EU019536	blaOXA
blaOXA-83_1_DQ309277	blaOXA
blaOXA-84_1_DQ309276	blaOXA
blaOXA-85_1_AY227054	blaOXA
blaOXA-86_1_DQ149247	blaOXA
blaOXA-87_1_DQ348075	blaOXA
blaOXA-88_1_DQ392963	blaOXA
blaOXA-89_1_EU547444	blaOXA
blaOXA-89_2_DQ445683	blaOXA
blaOXA-90_1_EU547443	blaOXA
blaOXA-91_1_DQ519086	blaOXA
blaOXA-92_1_DQ335566	blaOXA
blaOXA-93_1_DQ519087	blaOXA
blaOXA-94_1_DQ519088	blaOXA
blaOXA-95_1_DQ519089	blaOXA
blaOXA-96_1_DQ519090	blaOXA
blaOXA-97_1_EF102240	blaOXA
blaOXA-98_1_AM279652	blaOXA
blaOXA-99_1_DQ888718	blaOXA
blaOXA-9_2_JF703130	blaOXA
blaOXA-SHE_1_AY066004	blaOXA-SHE
blaOXY-1-1_1_Z30177	blaOXY
blaOXY-1-2_2_AJ871864	blaOXY
blaOXY-1-3_3_AY077482	blaOXY
blaOXY-1-4_4_AY077483	blaOXY
blaOXY-1-5_5_AY077486	blaOXY

blaOXY-1-6_6_Y17715	blaOXY
blaOXY-1-7_7_M27459	blaOXY
blaOXY-2-10_10_FJ785626	blaOXY
blaOXY-2-1_1_AJ871866	blaOXY
blaOXY-2-2_2_AJ871867	blaOXY
blaOXY-2-3_3_AY077488	blaOXY
blaOXY-2-4_4_Y17714	blaOXY
blaOXY-2-5_5_AY077487	blaOXY
blaOXY-2-6_6_AY077485	blaOXY
blaOXY-2-7_7_Z49084	blaOXY
blaOXY-2-8_8_AY055205	blaOXY
blaOXY-2-9_9_FJ785625	blaOXY
blaOXY-3-1_1_AF491278	blaOXY
blaOXY-4-1_1_AY077481	blaOXY
blaOXY-5-1_1_AJ871868	blaOXY
blaOXY-5-2_2_AJ871871	blaOXY
blaOXY-6-1_1_AJ871873	blaOXY
blaOXY-6-2_2_AJ871875	blaOXY
blaOXY-6-3_3_AJ871876	blaOXY
blaOXY-6-4_4_AJ871877	blaOXY
blaPAO_1_AY083595	blaPAO
blaPAO_2_FJ666065	blaPAO
blaPAO_3_FJ666073	blaPAO
blaPAO_4_AY083592	blaPAO
blaPER-1_1_GU944725	blaPER
blaPER-2_1_X93314	blaPER
blaPER-3_1_AY740681	blaPER
blaPER-4_1_EU748544	blaPER
blaPER-5_1_FJ627180	blaPER
blaPER-6_1_GQ396303	blaPER
blaPER-7_1_HQ713678	blaPER
blaPME-1_1_HQ541434	blaPME
blaRAHN-1_1_GU645205	blaRAHN
blaRAHN-1_2_AF338038	blaRAHN
blaRAHN-2_2_HM114350	blaRAHN
blaROB-1_1_DQ840517	blaROB
blaROB-1_2_AF022114	blaROB
blaSED1_1_AF321608	blaSED
blaSFC-1_1_AY354402	blaSFC
blaSFO-1_1_AB003148	blaSFO
blaSHV-100_1_AM941846	blaSHV
blaSHV-101_1_EU155018	blaSHV
blaSHV-102_2_EU024485	blaSHV
blaSHV-103_1_EU032604	blaSHV

blaSHV-104_1_EU274581	blaSHV
blaSHV-105_1_FJ194944	blaSHV
blaSHV-106_1_AM922307	blaSHV
blaSHV-107_1_AM922308	blaSHV
blaSHV-108_1_AM922309	blaSHV
blaSHV-109_1_EU418913	blaSHV
blaSHV-110_1_HQ877615	blaSHV
blaSHV-11_10_EF035557	blaSHV
blaSHV-11_11_GU064392	blaSHV
blaSHV-11_12_GU211012	blaSHV
blaSHV-11_13_DQ219476	blaSHV
blaSHV-11_14_DQ219474	blaSHV
blaSHV-11_15_DQ219473	blaSHV
blaSHV-11_1_AY528717	blaSHV
blaSHV-11_2_HM751098	blaSHV
blaSHV-11_3_EF035563	blaSHV
blaSHV-11_4_EF035558	blaSHV
blaSHV-11_5_GQ407117	blaSHV
blaSHV-11_6_GQ407130	blaSHV
blaSHV-11_7_GQ407109	blaSHV
blaSHV-11_8_GQ387358	blaSHV
blaSHV-11_9_FJ483937	blaSHV
blaSHV-120_1_JF812965	blaSHV
blaSHV-121_1_GQ428198	blaSHV
blaSHV-122_1_HM751103	blaSHV
blaSHV-128_1_GU932590	blaSHV
blaSHV-129_1_GU827715	blaSHV
blaSHV-129_2_HM125687	blaSHV
blaSHV-12_1_AF462395	blaSHV
blaSHV-12_2_AY273807	blaSHV
blaSHV-12_3_FJ668805	blaSHV
blaSHV-132_1_GU981741	blaSHV
blaSHV-133_1_AB551737	blaSHV
blaSHV-134_1_HM559945	blaSHV
blaSHV-135_1_HQ637576	blaSHV
blaSHV-136_1_HQ661362	blaSHV
blaSHV-137_1_HQ661363	blaSHV
blaSHV-13_1_AF164577	blaSHV
blaSHV-140_1_JN051143	blaSHV
blaSHV-141_1_JQ388884	blaSHV
blaSHV-142_1_JQ029959	blaSHV
blaSHV-143_1_JQ341060	blaSHV
blaSHV-144_1_JQ926986	blaSHV
blaSHV-145_1_JX013655	blaSHV

blaSHV-147_1_JX121114	blaSHV
blaSHV-148_1_JX121115	blaSHV
blaSHV-149_1_JX121116	blaSHV
blaSHV-14_1_AF226622	blaSHV
blaSHV-150_1_JX121117	blaSHV
blaSHV-151_1_JX121118	blaSHV
blaSHV-152_1_JX121119	blaSHV
blaSHV-153_1_JX121120	blaSHV
blaSHV-154_1_JX121121	blaSHV
blaSHV-155_1_JX121122	blaSHV
blaSHV-156_1_JX121123	blaSHV
blaSHV-157_1_JX121124	blaSHV
blaSHV-158_1_JX121125	blaSHV
blaSHV-159_1_JX121126	blaSHV
blaSHV-15_1_AJ011428	blaSHV
blaSHV-160_1_JX121127	blaSHV
blaSHV-161_1_JX121128	blaSHV
blaSHV-162_1_JX121129	blaSHV
blaSHV-163_1_JX121130	blaSHV
blaSHV-164_1_HE981194	blaSHV
blaSHV-165_1_JX121131	blaSHV
blaSHV-168_1_JX870080	blaSHV
blaSHV-16_1_AF072684	blaSHV
blaSHV-172_1_KF513177	blaSHV
blaSHV-173_1_KF513178	blaSHV
blaSHV-178_1_KF705209	blaSHV
blaSHV-179_1_KF705208	blaSHV
blaSHV-18_1_AF132290	blaSHV
blaSHV-1_10_EF035567	blaSHV
blaSHV-1_11_EF035565	blaSHV
blaSHV-1_12_AY787643	blaSHV
blaSHV-1_13_AF462396	blaSHV
blaSHV-1_14_HM751099	blaSHV
blaSHV-1_15_FJ668804	blaSHV
blaSHV-1_16_FJ668800	blaSHV
blaSHV-1_17_FJ668799	blaSHV
blaSHV-1_18_FJ668818	blaSHV
blaSHV-1_1_DQ166783	blaSHV
blaSHV-1_2_X98100	blaSHV
blaSHV-1_3_X98098	blaSHV
blaSHV-1_4_GQ407127	blaSHV
blaSHV-1_5_GQ407119	blaSHV
blaSHV-1_6_GU083599	blaSHV
blaSHV-1_7_GU083598	blaSHV

blaSHV-1_8_GQ407137	blaSHV
blaSHV-1_9_HM751102	blaSHV
blaSHV-24_1_AB023477	blaSHV
blaSHV-25_1_GU064391	blaSHV
blaSHV-26_1_AF227204	blaSHV
blaSHV-27_1_AF293345	blaSHV
blaSHV-28_1_HM751101	blaSHV
blaSHV-28_2_EU441172	blaSHV
blaSHV-28_3_AF299299	blaSHV
blaSHV-29_1_AF301532	blaSHV
blaSHV-2_1_GQ407116	blaSHV
blaSHV-2_2_AF282921	blaSHV
blaSHV-2_4_GU064394	blaSHV
blaSHV-2a_13_X98102	blaSHV
blaSHV-30_1_AY661885	blaSHV
blaSHV-31_1_GU064396	blaSHV
blaSHV-31_2_AY277255	blaSHV
blaSHV-33_1_AY037779	blaSHV
blaSHV-34_1_AY036620	blaSHV
blaSHV-35_1_AY070258	blaSHV
blaSHV-36_1_AY677211	blaSHV
blaSHV-36_2_AF467947	blaSHV
blaSHV-37_1_AF467948	blaSHV
blaSHV-38_1_EU979559	blaSHV
blaSHV-40_1_AF535128	blaSHV
blaSHV-41_1_AF535129	blaSHV
blaSHV-42_1_EF035559	blaSHV
blaSHV-43_1_AY065991	blaSHV
blaSHV-44_1_AY259119	blaSHV
blaSHV-45_1_AF547625	blaSHV
blaSHV-46_1_AY210887	blaSHV
blaSHV-48_1_AY263404	blaSHV
blaSHV-49_1_AY528718	blaSHV
blaSHV-50_1_AY288915	blaSHV
blaSHV-51_1_AY289548	blaSHV
blaSHV-52_1_HQ845196	blaSHV
blaSHV-55_1_DQ054528	blaSHV
blaSHV-56_1_EU586041	blaSHV
blaSHV-56_2_AY352599	blaSHV
blaSHV-57_1_AY223863	blaSHV
blaSHV-59_1_AY790341	blaSHV
blaSHV-5_1_AF096930	blaSHV
blaSHV-5_2_AF462394	blaSHV
blaSHV-5_3_GQ407139	blaSHV

blaSHV-5_4_AY386369	blaSHV
blaSHV-5_5_S82452	blaSHV
blaSHV-5_6_EF653399	blaSHV
blaSHV-5_7_AY570962	blaSHV
blaSHV-5_8_AY502004	blaSHV
blaSHV-5_9_AY502000	blaSHV
blaSHV-60_1_AB302939	blaSHV
blaSHV-61_1_AJ866284	blaSHV
blaSHV-62_1_AJ866285	blaSHV
blaSHV-62_2_GU064395	blaSHV
blaSHV-63_1_EU342351	blaSHV
blaSHV-64_1_DQ174304	blaSHV
blaSHV-65_1_DQ174305	blaSHV
blaSHV-66_1_DQ174306	blaSHV
blaSHV-67_1_DQ174307	blaSHV
blaSHV-69_1_DQ174308	blaSHV
blaSHV-70_1_DQ013287	blaSHV
blaSHV-71_1_EF373974	blaSHV
blaSHV-71_2_DQ296194	blaSHV
blaSHV-72_1_AM176547	blaSHV
blaSHV-73_1_AM176548	blaSHV
blaSHV-74_1_AM176549	blaSHV
blaSHV-75_1_AM176550	blaSHV
blaSHV-76_1_AM176551	blaSHV
blaSHV-77_1_EF373975	blaSHV
blaSHV-78_1_AM176553	blaSHV
blaSHV-79_1_AM176554	blaSHV
blaSHV-7_1_U20270	blaSHV
blaSHV-80_1_AM176555	blaSHV
blaSHV-81_1_AM176556	blaSHV
blaSHV-82_1_AM176557	blaSHV
blaSHV-83_1_AM176558	blaSHV
blaSHV-85_1_DQ322460	blaSHV
blaSHV-86_1_DQ328802	blaSHV
blaSHV-89_1_DQ193536	blaSHV
blaSHV-8_1_ECU92041	blaSHV
blaSHV-92_1_DQ836922	blaSHV
blaSHV-93_1_EF373969	blaSHV
blaSHV-94_1_EF373970	blaSHV
blaSHV-95_1_EF373972	blaSHV
blaSHV-96_1_EF373971	blaSHV
blaSHV-97_1_EF373973	blaSHV
blaSHV-98_1_AM941844	blaSHV
blaSHV-99_1_AM941845	blaSHV

blaSHV-9_1_S82452	blaSHV
blaSIM-1_1_JF731030	blaSIM
blaSMB-1_1_AB636283	blaSMB
blaSME-1_1_Z28968	blaSME
blaSME-2_1_AF275256	blaSME
blaOXA-254_1_AB781687	blaOXA
blaOXA-255_1_KC479325	blaOXA
blaOXA-256_1_HE616889	blaOXA
blaTEM-102_1_AY040093	blaTEM
blaTEM-104_1_AF516719	blaTEM
blaTEM-105_1_AF516720	blaTEM
blaTEM-106_1_AY101578	blaTEM
blaTEM-107_1_AY101764	blaTEM
blaTEM-108_1_AF506748	blaTEM
blaTEM-109_1_AY628175	blaTEM
blaTEM-10_1_AF093512	blaTEM
blaTEM-110_1_AY072920	blaTEM
blaTEM-111_1_AF468003	blaTEM
blaTEM-112_1_AY589493	blaTEM
blaTEM-113_1_AY589494	blaTEM
blaTEM-114_1_AY589495	blaTEM
blaTEM-115_1_AF535127	blaTEM
blaTEM-116_4_GU188444	blaTEM
blaTEM-116_7_AY425988	blaTEM
blaTEM-11_1_AY874537	blaTEM
blaTEM-120_1_AY243512	blaTEM
blaTEM-85_1_AJ277414	blaTEM
blaTEM-86_1_AJ277415	blaTEM
blaTEM-87_1_AF250872	blaTEM
blaTEM-88_1_AY027590	blaTEM
blaTEM-8_1_X65252	blaTEM
blaTEM-90_1_AF351241	blaTEM
blaTEM-91_1_AB049569	blaTEM
blaTEM-92_1_AF143804	blaTEM
blaTEM-93_1_AJ318093	blaTEM
blaTEM-94_1_AJ318094	blaTEM
blaTEM-95_1_AJ308558	blaTEM
blaTEM-96_1_AY092401	blaTEM
blaTEM-97_1_AF397067	blaTEM
blaTEM-98_1_AF397068	blaTEM
blaTEM-99_1_AF397066	blaTEM
blaTLA-1_1_AF148067	blaTLA
blaTRU_1_EU046614	blaTRU
blaTUS-1_1_AF441287	blaTUS

blaVEB-1_1_HM370393	blaVEB
blaVEB-1_3_DQ393569	blaVEB
blaVEB-1_4_AF324834	blaVEB
blaTEM-142_1_DQ388882	blaTEM
blaTEM-143_1_DQ075245	blaTEM
blaTEM-144_1_DQ256080	blaTEM
blaTEM-145_1_DQ105528	blaTEM
blaTEM-146_1_DQ105529	blaTEM
blaTEM-147_1_DQ279850	blaTEM
blaTEM-148_1_AM087454	blaTEM
blaTEM-149_1_DQ369751	blaTEM
blaTEM-150_1_AM183304	blaTEM
blaTEM-151_1_DQ834729	blaTEM
blaTEM-152_1_DQ834728	blaTEM
blaTEM-153_1_KC149518	blaTEM
blaTEM-154_1_FJ807656	blaTEM
blaTEM-155_1_DQ679961	blaTEM
blaTEM-156_1_AM941159	blaTEM
blaTEM-157_1_DQ909059	blaTEM
blaTEM-158_1_EF534736	blaTEM
blaTEM-159_1_EF136376	blaTEM
blaTEM-15_1_AM849805	blaTEM
blaTEM-160_1_EF136377	blaTEM
blaTEM-162_1_EF468463	blaTEM
blaTEM-163_1_EU815939	blaTEM
blaTEM-164_1_EU274580	blaTEM
blaTEM-166_1_FJ197316	blaTEM
blaTEM-167_1_FJ360884	blaTEM
blaTEM-168_1_FJ919776	blaTEM
blaTEM-169_1_FJ873740	blaTEM
blaTEM-16_1_X65254	blaTEM
blaTEM-171_1_GQ149347	blaTEM
blaTEM-176_1_GU550123	blaTEM
blaTEM-177_1_FN652295	blaTEM
blaTEM-178_1_X97254	blaTEM
blaTEM-17_1_Y14574	blaTEM
blaTEM-182_1_HQ317449	blaTEM
blaTEM-183_1_HQ529916	blaTEM
blaTEM-184_1_FR848831	blaTEM
blaTEM-185_1_JF795538	blaTEM
blaTEM-186_1_JN227084	blaTEM
blaTEM-187_1_HM246246	blaTEM
blaTEM-188_1_JN211012	blaTEM
blaTEM-189_1_JN254627	blaTEM

blaTEM-190_1_JN416112	blaTEM
blaTEM-193_1_JN935135	blaTEM
blaTEM-194_1_JN935136	blaTEM
blaTEM-195_1_JN935137	blaTEM
blaTEM-197_1_HQ877606	blaTEM
blaTEM-198_1_AB700703	blaTEM
blaTEM-199_1_JX050178	blaTEM
blaTEM-1A_4_HM749966	blaTEM
blaTEM-1B_1_JF910132	blaTEM
blaTEM-1C_5_FJ560503	blaTEM
blaTEM-1D_83_AF188200	blaTEM
blaTEM-201_1_JX310327	blaTEM
blaTEM-205_1_KC900516	blaTEM
blaTEM-206_1_KC783461	blaTEM
blaTEM-207_1_KC818234	blaTEM
blaTEM-208_1_KC865667	blaTEM
blaTEM-209_1_KF240808	blaTEM
blaTEM-20_1_EU527189	blaTEM
blaTEM-211_1_KF513179	blaTEM
blaTEM-213_1_KF663615	blaTEM
blaTEM-21_1_Y17582	blaTEM
blaTEM-22_1_Y17583	blaTEM
blaTEM-24_1_GQ293500	blaTEM
blaTEM-28_1_ECU37195	blaTEM
blaTEM-29_1_DQ269440	blaTEM
blaTEM-2_1_X54606	blaTEM
blaTEM-2_3_AJ251946	blaTEM
blaTEM-30_1_AJ437107	blaTEM
blaTEM-33_1_GU371926	blaTEM
blaTEM-34_1_KC292503	blaTEM
blaTEM-3_1_X64523	blaTEM
blaTEM-43_1_KPU95363	blaTEM
blaTEM-45_1_X95401	blaTEM
blaTEM-47_1_Y10279	blaTEM
blaTEM-48_1_Y10280	blaTEM
blaTEM-49_1_Y10281	blaTEM
blaTEM-52B_1_AF027199	blaTEM
blaTEM-52C_2_EF141186	blaTEM
blaTEM-53_1_AF104441	blaTEM
blaTEM-54_1_AF104442	blaTEM
blaTEM-55_1_DQ286729	blaTEM
blaTEM-57_1_FJ405211	blaTEM
blaTEM-60_1_AF047171	blaTEM
blaTEM-63_1_AF332513	blaTEM

blaTEM-67_1_AF091113	blaTEM
blaTEM-68_1_AJ239002	blaTEM
blaTEM-6_1_X57972	blaTEM
blaTEM-70_1_AF188199	blaTEM
blaTEM-71_1_AF203816	blaTEM
blaTEM-72_1_AF157553	blaTEM
blaTEM-76_1_AF190694	blaTEM
blaTEM-77_1_AF190695	blaTEM
blaTEM-78_1_AF190693	blaTEM
blaTEM-79_1_AF190692	blaTEM
blaTEM-80_1_AF347054	blaTEM
blaTEM-81_1_AF427127	blaTEM
blaTEM-82_1_AF427128	blaTEM
blaTEM-83_1_AF427129	blaTEM
blaTEM-84_1_AF427130	blaTEM
blaVIM-23_1_GQ242167	blaVIM
blaVIM-24_1_HM855205	blaVIM
blaVIM-25_1_HM750249	blaVIM
blaVIM-26_1_FR748153	blaVIM
cphA1_1_AY261379	cphA
cphA1_2_AY261377	cphA
cphA1_3_AY261378	cphA
cphA1_4_AY261376	cphA
cphA1_7_X57102	cphA
cphA2_6_AHU60294	cphA
cphA4_1_AY227050	cphA
cphA5_1_AY227051	cphA
cphA6_1_AY227052	cphA
cphA7_1_AY227053	cphA
cphA8_1_AY261375	cphA
blaVIM-37_1_JX982636	blaVIM
blaVIM-38_1_KC469971	blaVIM
blaVIM-3_1_AF300454	blaVIM
blaVIM-4_1_EU581706	blaVIM
blaVIM-5_1_DQ023222	blaVIM
blaVIM-6_1_AY165025	blaVIM
blaVEB-2_1_AY027870	blaVEB
blaVEB-3_1_AY536519	blaVEB
blaVEB-4_1_EF136375	blaVEB
blaVEB-5_1_EF420108	blaVEB
blaVEB-6_1_EU259884	blaVEB
blaVEB-7_1_FJ825622	blaVEB
blaVEB-8_1_JX679208	blaVEB
blaVIM-10_1_AY524989	blaVIM

blaVIM-11_1_AY605049	blaVIM
blaVIM-12_1_DQ143913	blaVIM
blaVIM-13_1_DQ365886	blaVIM
blaVIM-14_1_FJ445404	blaVIM
blaVIM-15_1_EU419745	blaVIM
blaVIM-16_1_EU419746	blaVIM
blaVIM-17_1_EU118148	blaVIM
blaVIM-18_1_AM778091	blaVIM
blaVIM-19_1_FJ499397	blaVIM
blaVIM-1_1_Y18050	blaVIM
blaVIM-20_1_GQ414736	blaVIM
mecA_5_CP000046	mecA
mecA_6_FR821779	mecA
mecA_7_BA000018	mecA
cepA_1_L13472	cepA
blaVIM-27_1_HQ858608	blaVIM
blaVIM-28_1_JF900599	blaVIM
blaVIM-29_1_JX311308	blaVIM
blaVIM-2_1_AF302086	blaVIM
blaVIM-30_1_JN129451	blaVIM
blaVIM-31_1_JN982330	blaVIM
blaVIM-32_1_JN676230	blaVIM
blaVIM-33_1_JX258134	blaVIM
blaVIM-34_1_JX013656	blaVIM
blaVIM-35_1_JX982634	blaVIM
blaVIM-36_1_JX982635	blaVIM
cepA-49_1_U05886	cepA
blaZ_40_DQ269019	blaZ
cepA_1_U05887	cepA
cepA_6_FR688022	cepA
cfiA10_1_AB087227	cfiA
cfiA13_1_FM200787	cfiA
blaVIM-7_1_AJ536835	blaVIM
blaVIM-8_1_AY524987	blaVIM
blaVIM-9_1_AY524988	blaVIM
blaZEG-1_1_AY265891	blaZEG
blaZ_32_AP004832	blaZ
blaZ_34_AP003139	blaZ
blaZ_35_AJ302698	blaZ
blaZ_36_AJ400722	blaZ
cfiA8_1_AB087233	cfiA
cfiA9_1_AB087234	cfiA
mecA_10_AB512767	mecA
cfxA2_1_AF504914	cfxA

cfxA3_1_AF472622	cfxA
cfxA4_1_AY769933	cfxA
mecA_4_AB033763	mecA
mecA_14_AB505630	mecA
mecA_15_AB505628	mecA
mecA_2_FR823292	mecA
penA_1_AF515059	penA
cepA-29_1_U05884	cepA
cepA-44_1_U05885	cepA
hugA_1_AF324468	hugA
cfiA2_1_AB087226	cfiA
cfiA14_1_FM200789	cfiA
cfiA16_1_FM200792	cfiA
cfiA1_1_AB087225	cfiA
cfxA6_1_GQ342996	cfxA
cfiA3_1_AB087228	cfiA
cfiA4_1_AB087229	cfiA
cfiA6_1_AB087231	cfiA
imiS_1_Y10415	imiS
imiH_1_AJ548797	imiH
cfxA_2_AY769933	cfxA
cfxA5_1_AY769934	cfxA
cfxA_1_U38243	cfxA
blaSPM-1_1_AY341249	blaSPM
blaTEM-101_1_AF495873	blaTEM
blaSME-3_1_AY584237	blaSME
aac(6')Ib-cr_3_EF210035	aac(6')I
aac(6')Ib-cr_2_EF636461	aac(6')I
aac(_1_DQ303918	aac(6')I
norA_1_M97169	norA
oqxB_1_EU370913	oqxB
oqxA_1_EU370913	oqxA
fosA_13_EU487198	fosA
fosA_12_AY692231	fosA
fosB_1_X54227	fosB
fosB_2_X89875	fosB
fosA_15_CP002889	fosA
fosA_16_M85195	fosA
fosA_3_NZ_ACWO01000079	fosA
fosA_11_DQ396803	fosA
fosA_8_ACHE01000077	fosA
fosA_9_NZ_ACZD01000244	fosA
fosC_2_Z33413	fosC
fosA_14_AB522970	fosA

fosB_3_HQ219726	fosB
fosC_1_AB522969	fosC
fosA_6_NZ_DS999363	fosA
fosA_2_AGDM01000012	fosA
fosA_4_NZ_AFHW01000027	fosA
fosA_5_NZ_AEXB01000013	fosA
fosA_10_EU195449	fosA
fosA_7_NZ_AFBO01000747	fosA
fusB_3_JF777505	fusB
fusB_1_AM292600	fusB
far1_1_DQ269019	far1
fusB_2_JF808725	fusB
lnu(B)_1_AJ238249	lnu(B)
lnu(C)_1_AY928180	lnu(C)
lmr(B)_1_X62867	lmr(B)
lnu(A)_1_M14039	lnu(A)
lnu(F)_3_AJ561197	lnu(F)
lmr(A)_1_X59926	lmr(A)
lnu(F)_1_EU118119	lnu(F)
lnu(F)_2_DQ836009	lnu(F)
lnu(D)_1_EF452177	lnu(D)
erm(33)_2_AJ579365	erm(33)
erm(41)_1_EU177504	erm(41)
erm(34)_1_AY234334	erm(34)
erm(32)_1_AJ009971	erm(32)
erm(42)_1_FR734406	erm(42)
erm(35)_1_AF319779	erm(35)
erm(C)_2_M19652	erm(C)
erm(E)_2_M11200	erm(E)
erm(D)_2_L08389	erm(D)
erm(42)_2_AB601890	erm(42)
erm(C)_1_V01278	erm(C)
erm(40)_1_AY570506	erm(40)
erm(B)_1_JN899585	erm(B)
erm(D)_3_M77505	erm(D)
erm(E)_1_X51891	erm(E)
erm(B)_2_K00551	erm(B)
erm(D)_1_M29832	erm(D)
erm(B)_20_AF109075	erm(B)
erm(B)_21_U35228	erm(B)
erm(C)_10_Y09002	erm(C)
erm(C)_12_Y09003	erm(C)
erm(B)_7_AF368302	erm(B)
erm(B)_9_AF299292	erm(B)

erm(C)_16_AF019140	erm(C)
ere(A)_1_AY138453	ere(A)
ere(A)_2_AF099140	ere(A)
ere(A)_3_AF326209	ere(A)
ere(A)_4_AF512546	ere(A)
ere(B)_1_A15097	ere(B)
erm(B)_6_AF242872	erm(B)
erm(30)_1_AF079138	erm(30)
erm(31)_1_AF079138	erm(31)
mef(A)_10_AF376746	mef(A)
mef(A)_3_AF227521	mef(A)
erm(36)_1_AF462611	erm(36)
erm(38)_1_AY154657	erm(38)
erm(39)_1_AY487229	erm(39)
mph(B)_1_D85892	mph(B)
mph(C)_1_AB013298	mph(C)
mph(C)_2_AF167161	mph(C)
mph(D)_1_AB048591	mph(D)
mph(E)_3_EU294228	mph(E)
mph(E)_5_DQ839391	mph(E)
erm(A)_1_X03216	erm(A)
erm(A)_2_AF002716	erm(A)
erm(A)_3_EU348758	erm(A)
erm(B)_10_U86375	erm(B)
mef(B)_1_FJ196385	mef(B)
mph(A)_1_D16251	mph(A)
mph(A)_2_U36578	mph(A)
erm(B)_16_X82819	erm(B)
erm(B)_17_X64695	erm(B)
erm(B)_18_X66468	erm(B)
erm(W)_1_D14532	erm(W)
erm(X)_1_M36726	erm(X)
erm(X)_2_X51472	erm(X)
erm(X)_3_U21300	erm(X)
erm(X)_4_NC_005206	erm(X)
erm(Y)_1_AB014481	erm(Y)
lsa(A)_1_AY225127	lsa(A)
lsa(A)_2_AY58982	lsa(A)
erm(C)_13_M13761	erm(C)
erm(C)_14_M12730	erm(C)
erm(C)_15_U82607	erm(C)
erm(F)_3_M17808	erm(F)
erm(F)_4_M62487	erm(F)
erm(G)_1_M15332	erm(G)

erm(G)_2_L42817	erm(G)
erm(C)_3_M17990	erm(C)
erm(C)_6_X82668	erm(C)
erm(C)_9_Y09001	erm(C)
ere(B)_2_X03988	ere(B)
erm(Q)_1_L22689	erm(Q)
erm(R)_2_AY623658	erm(R)
erm(B)_11_M19270	erm(B)
erm(B)_12_U18931	erm(B)
erm(F)_2_M17124	erm(F)
erm(T)_3_AF310974	erm(T)
erm(T)_4_AJ488494	erm(T)
erm(V)_1_U59450	erm(V)
erm(O)_2_AJ223970	erm(O)
erm(H)_1_M16503	erm(H)
erm(N)_1_X97721	erm(N)
erm(O)_1_M74717	erm(O)
lsa(A)_3_AY737526	lsa(A)
erm(B)_15_U48430	erm(B)
erm(Z)_1_AM709783	erm(Z)
erm(T)_2_AY894138	erm(T)
erm(S)_1_M19269	erm(S)
lsa(C)_1_HM990671	lsa(C)
erm(T)_1_M64090	erm(T)
lsa(B)_1_AJ579365	lsa(B)
car(A)_1_M80346	car(A)
msr(A)_1_X52085	msr(A)
ole(B)_1_L36601	ole(B)
msr(C)_1_AY004350	msr(C)
tlr(C)_1_M57437	tlr(C)
msr(E)_5_DQ839391	msr(E)
srm(B)_1_X63451	srm(B)
msr(A)_2_AB013298	msr(A)
msr(A)_3_M81802	msr(A)
msr(D)_3_AF227520	msr(D)
msr(E)_7_AF550415	msr(E)
msr(D)_2_AF274302	msr(D)
msr(E)_4_EU294228	msr(E)
msr(C)_2_AF313494	msr(C)
ole(C)_1_L06249	ole(C)
catB9_1_AF462019	catB
cat(pC194)_1_NC_002013	cat
catS_1_X74948	catS
cmrA_1_Z12001	cmrA

catB10_1_AJ878850	catB
catP_1_U15027	catP
catB8_1_AF227506	catB
cat(pC233)_1_AY355285	cat
cmx_1_U85507	cmx
cat_2_M35190	cat
catQ_1_M55620	catQ
catA2_1_X53796	catA
catA3_1_X07848	catA
cat_5_U35036	cat
catB1_1_M58472	catB
cml_1_M22614	cml
catB3_1_AJ009818	catB
catB7_1_AF036933	catB
catB2_1_AF047479	catB
cat(pC221)_1_X02529	cat
cat_3_S48276	cat
cat86_1_K00544	cat
floR_3_AJ251806	floR
catA1_1_V00622	catA
cmlA1_1_M64556	cmlA
cat_4_AY238971	cat
cmlV_1_U09991	cmlV
cat_1_M11587	cat
pexA_1_HM537013	pexA
cmlA1_2_AB212941	cmlA
fexA_1_AJ549214	fexA
floR_1_AF071555	floR
floR_2_AF118107	floR
cfr_1_AM408573	cfr
cfr_1_AJ579365	cfr
QnrB58_1_JX259319	QnrB
QnrB57_1_JX259318	QnrB
QnrB6_3_EF523819	QnrB
QnrA4_1_DQ058662	QnrA
QnrA3_1_DQ058661	QnrA
QnrB6_2_EF517946	QnrB
QnrB16_1_EU136183	QnrB
QnrB17_1_JN173051	QnrB
QnrB70_1_KC580659	QnrB
QnrB6_1_EF520349	QnrB
QnrB72_1_KC741443	QnrB
QnrA1_1_AY070235	QnrA
QnrA2_1_HQ449669	QnrA

QnrB14_1_EU273757	QnrB
QnrB15_1_EU302865	QnrB
QnrB23_1_FJ981622	QnrB
QnrB24_1_HM192542	QnrB
QnrB59_1_JX259320	QnrB
QnrB5_1_DQ303919	QnrB
QnrB60_1_AB734055	QnrB
QnrB2_1_HM125698	QnrB
QnrB2_2_DQ351242	QnrB
QnrB30_1_HM439650	QnrB
QnrB31_1_HQ418999	QnrB
QnrB32_1_HQ704413	QnrB
QnrB32_2_JN173054	QnrB
QnrA5_1_DQ058663	QnrA
QnrA6_1_DQ151889	QnrA
QnrB71_1_KC580660	QnrB
QnrB7_1_EU043311	QnrB
QnrB8_1_EU043312	QnrB
QnrB9_1_EF653270	QnrB
QnrC_1_EU917444	QnrC
QnrB12_2_AM774474	QnrB
QnrB13_1_EU273755	QnrB
QnrB13_1_JN173050	QnrB
QnrB13_2_EU273756	QnrB
QnrB44_1_JQ349153	QnrB
QnrB45_1_JQ349152	QnrB
QnrB46_1_JQ349154	QnrB
QnrB17_2_AM919398	QnrB
QnrB18_1_AM919399	QnrB
QnrAS_1_FM178379	QnrA
QnrB10_1_DQ631414	QnrB
QnrB10_8_HM439644	QnrB
QnrB11_1_EU136183	QnrB
QnrB1_1_EF682133	QnrB
QnrB1_2_DQ351241	QnrB
QnrB20_1_AB379831	QnrB
QnrB21_1_FJ611948	QnrB
QnrB22_1_FJ981621	QnrB
QnrB56_1_JX259317	QnrB
QnrB69_1_KC580658	QnrB
QnrB25_1_HQ172108	QnrB
QnrB26_1_HM439644	QnrB
QnrB27_1_HM439641	QnrB
QnrB27_2_HM439642	QnrB

QnrB28_1_HM439643	QnrB
QnrB29_1_HM439649	QnrB
QnrB29_2_JN173053	QnrB
QnrB64_1_KC580653	QnrB
QnrB65_1_KC580654	QnrB
QnrB66_1_KC580655	QnrB
QnrB67_1_KC580656	QnrB
QnrB68_1_KC580657	QnrB
QnrS1_1_GQ336885	QnrS
QnrB33_1_JN173055	QnrB
QnrB34_1_JN173056	QnrB
QnrB35_1_JN173057	QnrB
QnrB36_1_JN173058	QnrB
QnrB37_1_JN173059	QnrB
QnrB38_1_JN173060	QnrB
QnrB3_1_DQ303920	QnrB
QnrB40_1_JN166689	QnrB
QnrB41_1_JN166690	QnrB
QnrB42_1_JN680743	QnrB
QnrB43_1_AFA52643	QnrB
QnrS1_1_AB187515	QnrS
QnrS4_1_FJ418153	QnrS
QnrS2_1_JF261185	QnrS
QnrS2_2_DQ485530	QnrS
QnrS3_1_EU077611	QnrS
QnrB50_1_JX440357	QnrB
QnrB4_1_DQ303921	QnrB
QnrB19_1_EU432277	QnrB
QnrB19_1_HM146784	QnrB
QnrB54_1_HE820727	QnrB
QnrB51_1_JX440358	QnrB
QnrB52_1_EF488762	QnrB
QnrB53_1_HQ704413	QnrB
QnrB61_1_AB734053	QnrB
QnrB62_1_JX987101	QnrB
QnrA7_1_GQ463707	QnrA
QnrB48_1_JQ762640	QnrB
QnrD_1_FJ228229	QnrD
QnrD_2_HM056768	QnrD
QnrVC3_1_HM015626	QnrV
QnrVC4_1_GQ891757	QnrV
QnrVC1_1_EU436855.2	QnrV
qepA2_1_EU847537	qepA
qepA_1_AB263754	qepA

QnrB47_1_JQ349155	QnrB
QnrS5_1_HQ631377	QnrS
QnrVC5_1_JN408080	QnrV
QnrB4_2_HM125703	QnrB
QnrS6_1_HQ631376	QnrS
QnrB49_1_JQ582718	QnrB
QnrB4_3_EF543140	QnrB
ARR-2_1_HQ141279	ARR
ARR-2_2_AF078527	ARR
ARR-3_1_JF806499	ARR
ARR-3_2_FJ459817	ARR
ARR-3_3_AJ277027	ARR
ARR-3_4_FM207631	ARR
ARR-4_1_EF660562	ARR
ARR-5_1_EF660563	ARR
ARR-6_1_FM955585	ARR
ARR-6_2_FM897214	ARR
ARR-6_3_JF922883	ARR
ARR-7_1_FN397623	ARR
vat(A)_1_L07778	vat(A)
vat(B)_1_U19459	vat(B)
vat(C)_1_AF015628	vat(C)
vat(D)_1_L12033	vat(D)
vat(D)_2_AF368302	vat(D)
vat(E)_10_AY043212	vat(E)
vat(E)_11_AY043213	vat(E)
vat(E)_3_AF153312	vat(E)
vat(E)_5_AJ488494	vat(E)
vat(E)_6_AF242872	vat(E)
vat(E)_7_AY043211	vat(E)
vat(E)_8_AY043209	vat(E)
vat(E)_9_AY043210	vat(E)
vat(F)_1_AF170730	vat(F)
vga(A)LC_1_GQ891882	vga(A)
vga(A)_1_M90056	vga(A)
vga(A)_2_FN546261	vga(A)
vga(B)_1_U82085	vga(B)
vga(C)_1_GQ205627	vga(C)
vga(C)_2_NC_013034	vga(C)
vga(E)_1_FR772051	vga(E)
vgb(A)_1_M20129	vgb(A)
vgb(B)_1_AF015628	vgb(B)
sul2_8_AJ877041	sul2
sul2_9_FJ197818	sul2

sul3_1_EU834941	sul3
sul3_2_AJ459418	sul3
sul3_3_AY047357	sul3
sul3_5_AB281182	sul3
sul3_6_AB281183	sul3
sul1_10_DQ143913	sul1
sul1_11_DQ914960	sul1
sul1_14_AJ517791	sul1
sul1_15_EF667294	sul1
sul1_16_EF667294	sul1
sul1_17_AM746675	sul1
sul1_18_AY260546	sul1
sul1_19_DQ125241	sul1
sul1_20_JF262165	sul1
sul1_21_AJ971344	sul1
sul1_22_AY115475	sul1
sul1_23_AJ621187	sul1
sul1_24_EU117158	sul1
sul1_25_AY878717	sul1
sul1_26_AY524415	sul1
sul1_27_GU562437	sul1
sul1_28_JN790946	sul1
sul1_29_AJ746361	sul1
sul1_2_CP002151	sul1
sul1_30_JF262178	sul1
sul1_31_AM040449	sul1
sul1_32_EF592571	sul1
sul1_33_AJ564903	sul1
sul1_3_EU855787	sul1
sul1_4_HQ713678	sul1
sul1_5_EU780013	sul1
sul1_6_EU855787	sul1
sul1_7_FJ715937	sul1
sul1_8_JN581942	sul1
sul1_9_AY963803	sul1
sul2_10_AM183225	sul2
sul2_11_AY232670	sul2
sul2_12_AF497970	sul2
sul2_13_AJ289135	sul2
sul2_14_AJ514834	sul2
sul2_15_FJ968160	sul2
sul2_16_FM179941	sul2
sul2_17_U57647	sul2
sul2_18_AJ830714	sul2

sul2_19_AJ319822	sul2
sul2_1_AF542061	sul2
sul2_20_AJ830710	sul2
sul2_2_GQ421466	sul2
sul2_3_HQ840942	sul2
sul2_4_AY333434	sul2
sul2_5_AY524415	sul2
sul2_6_FN995456	sul2
sul2_7_HM486907	sul2
otr(A)_1_X53401	otr(A)
otr(C)_1_AY509111	otr(C)
tet(L)_7_D12567	tet(L)
tet(L)_8_X60828	tet(L)
tet(L)_9_AY081910	tet(L)
ort(B)_2_AF061335	otr(B)
tet(M)_11_JN846696	tet(M)
tet(M)_12_FR671418	tet(M)
tet(M)_13_AM990992	tet(M)
tet(M)_10_EU182585	tet(M)
tet(M)_2_X90939	tet(M)
tet(M)_3_U08812	tet(M)
tet(M)_4_X75073	tet(M)
tet(M)_5_U58985	tet(M)
tet(M)_6_M21136	tet(M)
tet(M)_8_X04388	tet(M)
tet(M)_9_X56353	tet(M)
tet(O)_1_M18896	tet(O)
tet(O)_2_M20925	tet(O)
tet(O)_3_Y07780	tet(O)
tet(Q)_1_L33696	tet(Q)
tet(Q)_2_X58717	tet(Q)
tet(Q)_3_U73497	tet(Q)
tet(Q)_4_Z21523	tet(Q)
tet(S)_1_DQ377340	tet(S)
tet(S)_2_L09756	tet(S)
tet(S)_3_X92946	tet(S)
tet(T)_1_L42544	tet(T)
tet(T)_2_AY660530	tet(T)
tet(U)_1_U01917	tet(U)
tet(V)_1_AF030344	tet(V)
tet(W)_1_DQ060146	tet(W)
tet(W)_2_AY049983	tet(W)
tet(W)_4_AJ427422	tet(W)
tet(W)_5_AJ427421	tet(W)

tet(W)_6_FN396364	tet(W)
tet(X)_1_GU014535	tet(X)
tet(X)_2_M37699	tet(X)
tet(X)_3_AB097942	tet(X)
tet(Y)_1_EF495198	tet(Y)
tet(Y)_5_AB089606	tet(Y)
tet(Z)_2_AF121000	tet(Z)
tet(Z)_3_AY222818	tet(Z)
tetA(P)_1_AB054980	tetA(P)
tetA(P)_2_HQ399624	tetA(P)
tetA(P)_3_L20800	tetA(P)
tetA(P)_4_AB001076	tetA(P)
tetB(P)_1_DQ366035	tetB(P)
tetB(P)_3_NC_010937	tetB(P)
tet(M)_1_X92947	tet(M)
tet(B)_4_AF326777	tet(B)
tet(B)_5_AJ277653	tet(B)
tet(C)_10_AY043299	tet(C)
tet(C)_1_NC_002109	tet(C)
tet(C)_2_NC_003123	tet(C)
tet(C)_5_NC_003213	tet(C)
tet(C)_6_Y19114	tet(C)
tcr_1_D38215	tcr
tet(30)_1_AF090987	tet(30)
tet(31)_1_GQ283908	tet(31)
tet(31)_2_AJ250203	tet(31)
tet(32)_1_EU722333	tet(32)
tet(32)_2_EF626943	tet(32)
tet(33)_1_DQ077487	tet(33)
tet(33)_2_AY255627	tet(33)
tet(33)_3_DQ390458	tet(33)
tet(34)_1_AB061440	tet(34)
tet(35)_1_AF353562	tet(35)
tet(36)_1_AJ514254	tet(36)
tet(37)_1_AF540889	tet(37)
tet(38)_1_AY825285	tet(38)
tet(38)_2_FN433596	tet(38)
tet(38)_3_FR821779	tet(38)
tet(39)_2_EU495989	tet(39)
tet(39)_2_EU495991	tet(39)
tet(39)_3_DQ195075	tet(39)
tet(40)_1_FJ158002	tet(40)
tet(40)_2_FJ158002	tet(40)
tet(40)_3_AM419751	tet(40)

tet(41)_1_AY264780	tet(41)
tet(42)_1_EU523697	tet(42)
tet(43)_1_GQ244501	tet(43)
tet(44)_1_NZ_ABDU01000081	tet(44)
tet(44)_2_FN594949	tet(44)
tet(A)_2_X00006	tet(A)
tet(A)_3_AY196695	tet(A)
tet(A)_4_AJ517790	tet(A)
tet(A)_5_AJ419171	tet(A)
tet(A)_6_AJ313332	tet(A)
tet(B)_3_AP000342	tet(B)
tet(E)_3_L06940	tet(E)
tet(E)_5_CP000645	tet(E)
tet(G)_1_AJ276217	tet(G)
tet(G)_2_AF133139	tet(G)
tet(G)_3_S52437	tet(G)
tet(G)_4_AF133140	tet(G)
tet(G)_5_AF071555	tet(G)
tet(C)_9_AY046276	tet(C)
tet(D)_1_AF467077	tet(D)
tet(D)_3_D16172	tet(D)
tet(D)_5_X65876	tet(D)
tet(E)_1_Y19116	tet(E)
tet(E)_2_EF471995	tet(E)
tet(K)_4_U38428	tet(K)
tet(K)_5_J01764	tet(K)
tet(L)_1_HM235948	tet(L)
tet(L)_2_M29725	tet(L)
tet(L)_4_M11036	tet(L)
tet(L)_5_D00006	tet(L)
tet(L)_6_X08034	tet(L)
tet(H)_1_Y16103	tet(H)
tet(H)_2_AJ245947	tet(H)
tet(H)_3_Y15510	tet(H)
tet(H)_4_U00792	tet(H)
tet(J)_1_ACLE01000065	tet(J)
tet(J)_2_AF038993	tet(J)
dfrB5_1_AY943084	dfrB
dfrA30_1_AM997279	dfrA
dfrG_1_AB205645	dfrG
dfrA21_1_AY552589	dfrA
dfrB6_1_DQ274503	dfrB
dfrA29_1_AM237806	dfrA
dfrA21_1_AM932669	dfrA

dfrB8_1_GU295656	dfrB
dfrA22_1_HM173356	dfrA
dfrA23_1_AJ746361	dfrA
dfrB7_1_DQ993182	dfrB
dfrA8_1_U10186	dfrA
dfrA31_1_AB200915	dfrA
dfrA28_2_FM877476	dfrA
dfrD_1_Z50141	dfrD
dfrA33_1_FM957884	dfrA
dfrA3_1_J03306	dfrA
dfrA3b_1_AY878717	dfrAb
dfrA5_1_X12868	dfrA
dfrA6_1_Z86002	dfrA
dfrA7_1_AJ419170	dfrA
dfrC_1_GU565967	dfrC
dfrC_1_Z48233	dfrC
dfrA18_1_AJ310778	dfrA
dfrA1_1_X00926	dfrA
dfrA1_25_AJ844287	dfrA
dfrK_1_FN377602	dfrK
dfrA32_1_GU067642	dfrA
dfrB3_1_FM877478	dfrB
dfrB3_1_X72585	dfrB
dfrB4_1_FM877484	dfrB
dfrA15b_1_AJ867237	dfrAb
dfrA16_1_AF077008	dfrA
dfrA16_1_DQ316603	dfrA
dfrA16_1_FN561628	dfrA
dfrA27_1_FJ459817	dfrA
dfrA7_2_X58425	dfrA
dfrA7_3_HM999792	dfrA
dfrA10_1_L06418	dfrA
dfrA12_1_AB571791	dfrA
dfrK_4_FN677369	dfrK
dfrK_8_FN812951	dfrK
dfrA20_1_AJ605332	dfrA
dfrA15_1_HM449019	dfrA
dfrA15_1_Z83311	dfrA
dfrA26_1_AM403715	dfrA
dfrA24_1_AJ972619	dfrA
dfrA25_1_DQ267940	dfrA
dfrA17_1_FJ460238	dfrA
dfrA13_1_Z50802	dfrA
dfrA14_1_DQ388123	dfrA

dfrA14_1_Z50805	dfrA
dfrA1_5_AJ238350	dfrA
dfrA7_1_JF806498	dfrA
dfrA1_2_AJ419168	dfrA
dfrA1_30_JQ690541	dfrA
dfrB2_1_DQ839391	dfrB
dfrA9_1_X57730	dfrA
dfrB1_2_U36276	dfrB
VanX-A_1_FJ866609	VanA
VanR-A_2_M97297	VanA
VanA-A_2_M97297	VanA
VanZ-A_1_FJ866609	VanA
VanS-A_2_M97297	VanA
VanY-A_2_M97297	VanA
VanZ-A_2_M97297	VanA
VanH-A_1_FJ866609	VanA
VanA-A_1_FJ866609	VanA
VanX-A_2_M97297	VanA
VanH-A_2_M97297	VanA
VanY-A_1_FJ866609	VanA
VanH-Ac1_6_DQ246438	VanAc1
VanX-Ac1_6_DQ246438	VanAc1
VanX-Ac2_7_AY618461	VanAc2
VanH-Ac2_7_AY618461	VanAc2
VanH-Ao1_8_AF060799	VanAo1
VanX-Ao1_8_AF060799	VanAo1
VanH-A(Bc)_7_Y15704	VanAo2
VanA-Ao2_6_HQ679900	VanAo2
VanH-Ao2_9_HQ679900	VanAo2
VanX-Ao2_9_HQ679900	VanAo2
VanR-B_1_AF192329	VanB
VanH-B_1_AF192329	VanB
VanA-B_1_AF192329	VanB
VanY-B_1_AF192329	VanB
VanW-B_1_AF192329	VanB
VanX-B_1_AF192329	VanB
VanS-B_1_AF192329	VanB
VanXY-C_5_EU151754	VanC
VanA-C_2_DQ022190	VanC
VanA-C_1_AF162694	VanC
VanR-C_2_DQ022190	VanC
VanA-C_3_EU151752	VanC
VanR-C_1_AF162694	VanC
VanR-C_3_EU151752	VanC

VanS-C_4_EU151753	VanC
VanA-C_5_EU151754	VanC
VanXY-C_4_EU151753	VanC
VanT-C_3_EU151753	VanC
VanA-C_4_EU151753	VanC
VanXY-C_1_AF162694	VanC
VanS-C_3_EU151752	VanC
VanXY-C_2_DQ022190	VanC
VanT-C_2_EU151752	VanC
VanR-C_4_EU151753	VanC
VanS-C_2_DQ022190	VanC
VanXY-C_3_EU151752	VanC
VanS-C_1_AF162694	VanC
VanT-C_1_DQ022190	VanC
VanT-C_1_AF162694	VanC
VanH-D_3_AY082011	VanD
VanX-D_2_AF175293	VanD
VanS-D_3_AY489045	VanD
VanH-D_2_AF175293	VanD
VanX-D_3_AY082011	VanD
VanH-D_4_AY489045	VanD
VanX-D_1_AB242319	VanD
VanY-D_4_AY489045	VanD
VanA-D_4_AY489045	VanD
VanX-D_4_AY489045	VanD
VanR-D_1_AB242319	VanD
VanS-D_2_AF175293	VanD
VanY-D_2_AF175293	VanD
VanA-D_2_AF175293	VanD
VanH-D_1_AB242319	VanD
VanR-D_4_AY489045	VanD
VanR-D_2_AF175293	VanD
VanY-D_3_AY082011	VanD
VanA-D_3_AY082011	VanD
VanS-D_1_AB242319	VanD
VanA-D_1_AB242319	VanD
VanR-D_3_AY082011	VanD
VanY-D_1_AB242319	VanD
VanH-E_1_AF155139	VanE
VanA-E_1_AF155139	VanE
VanX-E_1_AF155139	VanE
VanY-F_1_AF155139	VanF
VanS-F_1_AF155139	VanF
VanZ-F_1_AF155139	VanF

VanR-F_1_AF155139	VanF
VanU-G_1_AY271782	VanG
VanW-G_1_AY271782	VanG
VanY-G_1_DQ212986	VanG
VanT-G_1_AY271782	VanG
VanXY-G_1_AY271782	VanG
VanA-G_1_AY271782	VanG
VanR-G_1_AY271782	VanG
VanS-G_1_AY271782	VanG
VanXY-L_1_EU250284	VanL
VanS-L_1_EU250284	VanL
VanA-L_1_EU250284	VanL
VanTr-L_1_EU250284	VanL
VanTm-L_1_EU250284	VanL
VanR-L_1_EU250284	VanL
VanA-M_1_FJ349556	VanM
VanY-M_1_FJ349556	VanM
VanH-M_1_FJ349556	VanM
VanR-M_1_FJ349556	VanM
VanS-M_1_FJ349556	VanM
VanX-M_1_FJ349556	VanM
VanYX-N_1_JF802084	VanN
VanS-N_1_JF802084	VanN
VanA-N_1_JF802084	VanN
VanR-N_1_JF802084	VanN
VanT-N_1_JF802084	VanN
VanXE-Pp_2_AF155139	VanPp
VanHE-Pp_2_AF155139	VanPp
VanE-Pp_2_AF155139	VanPp
VanYF-Pp_2_AF155139	VanPp
VanZF-Pp_2_AF155139	VanPp
VanX-Pt_5_DQ018711	VanPt
VanA-Pt_3_DQ018710	VanPt
VanW-Pt_2_DQ018711	VanPt
VanY-Pt_3_DQ018710	VanPt
VanX-Pt_3_DQ018710	VanPt
VanZ-Pt_3_DQ018710	VanPt
VanH-Pt_5_DQ018711	VanPt
VanZ-Pt_4_DQ018711	VanPt
VanS-Pt_3_DQ018710	VanPt
VanS-Pt_5_DQ018711	VanPt
VanR-Pt_5_DQ018711	VanPt
VanR-Pt_3_DQ018710	VanPt
VanY-Pt_5_DQ018711	VanPt

VanA-Pt_5_DQ018711	VanPt
VanH-Pt_3_DQ018710	VanPt
VanS-Pt2_4_AY926880	VanPt2
VanX-Pt2_4_AY926880	VanPt2
VanA-Pt2_4_AY926880	VanPt2
VanY-Pt2_4_AY926880	VanPt2
VanH-Pt2_4_AY926880	VanPt2
VanR-Pt2_4_AY926880	VanPt2
VanX-Sc_1_AL939117	VanSc
VanH-Sc_1_AL939117	VanSc
ddlA2-Sc_2_AL939117	VanSc
nimA_1_X71444	nimA
nimF_1_AJ515145	nimF
nimH_1_FJ969397	nimH
nimI_1_FJ940883	nimI
nimE_1_AM042593	nimE
nimI_1_FJ940887	nimI
nimI_1_FJ940889	nimI
nimJ_1_NZ_JH815495	nimJ
nimE_1_AJ244018	nimE
nimB_1_X71443	nimB
nimC_1_X76948	nimC
nimD_1_X76949	nimD
nimI_1_FJ940884	nimI
nimI_1_FJ940886	nimI

:ntifiers to gene name and merged resistance classl

Resistance type	Merged resistance class
Aminoglycoside resistance	Aminoglycoside
Aminoglycoside resistance	Aminoglycoside
Aminoglycoside resistance	Aminoglycoside
Aminoglycoside resistance	Aminoglycoside
Aminoglycoside resistance	Aminoglycoside
Aminoglycoside resistance	Aminoglycoside
Aminoglycoside resistance	Aminoglycoside
Aminoglycoside resistance	Aminoglycoside
Aminoglycoside resistance	Aminoglycoside
Aminoglycoside resistance	Aminoglycoside
Aminoglycoside resistance	Aminoglycoside
Aminoglycoside resistance	Aminoglycoside
Aminoglycoside resistance	Aminoglycoside
Aminoglycoside resistance	Aminoglycoside
Aminoglycoside resistance	Aminoglycoside
Aminoglycoside resistance	Aminoglycoside
Aminoglycoside resistance	Aminoglycoside
Aminoglycoside resistance	Aminoglycoside
Aminoglycoside resistance	Aminoglycoside
Aminoglycoside resistance	Aminoglycoside
Aminoglycoside resistance	Aminoglycoside
Aminoglycoside resistance	Aminoglycoside
Aminoglycoside resistance	Aminoglycoside
Aminoglycoside resistance	Aminoglycoside
Aminoglycoside resistance	Aminoglycoside
Aminoglycoside resistance	Aminoglycoside
Aminoglycoside resistance	Aminoglycoside
Aminoglycoside resistance	Aminoglycoside
Aminoglycoside resistance	Aminoglycoside
Aminoglycoside resistance	Aminoglycoside
Aminoglycoside resistance	Aminoglycoside
Aminoglycoside resistance	Aminoglycoside
Aminoglycoside resistance	Aminoglycoside
Aminoglycoside resistance	Aminoglycoside
Aminoglycoside resistance	Aminoglycoside
Aminoglycoside resistance	Aminoglycoside
Aminoglycoside resistance	Aminoglycoside
Aminoglycoside resistance	Aminoglycoside
Aminoglycoside resistance	Aminoglycoside
Aminoglycoside resistance	Aminoglycoside
Aminoglycoside resistance	Aminoglycoside
Aminoglycoside resistance	Aminoglycoside

Aminoglycoside resistance	Aminoglycoside
Aminoglycoside resistance	Aminoglycoside
Aminoglycoside resistance	Aminoglycoside
Aminoglycoside resistance	Aminoglycoside
Aminoglycoside resistance	Aminoglycoside
Aminoglycoside resistance	Aminoglycoside
Aminoglycoside resistance	Aminoglycoside
Aminoglycoside resistance	Aminoglycoside
Aminoglycoside resistance	Aminoglycoside
Aminoglycoside resistance	Aminoglycoside
Aminoglycoside resistance	Aminoglycoside
Aminoglycoside resistance	Aminoglycoside
Aminoglycoside resistance	Aminoglycoside
Aminoglycoside resistance	Aminoglycoside
Aminoglycoside resistance	Aminoglycoside
Aminoglycoside resistance	Aminoglycoside
Aminoglycoside resistance	Aminoglycoside
Aminoglycoside resistance	Aminoglycoside
Aminoglycoside resistance	Aminoglycoside
Aminoglycoside resistance	Aminoglycoside
Aminoglycoside resistance	Aminoglycoside
Aminoglycoside resistance	Aminoglycoside
Aminoglycoside resistance	Aminoglycoside
Aminoglycoside resistance	Aminoglycoside
Aminoglycoside resistance	Aminoglycoside
Aminoglycoside resistance	Aminoglycoside
Aminoglycoside resistance	Aminoglycoside
Aminoglycoside resistance	Aminoglycoside
Aminoglycoside resistance	Aminoglycoside
Aminoglycoside resistance	Aminoglycoside
Aminoglycoside resistance	Aminoglycoside
Aminoglycoside resistance	Aminoglycoside
Aminoglycoside resistance	Aminoglycoside
Aminoglycoside resistance	Aminoglycoside
Aminoglycoside resistance	Aminoglycoside
Aminoglycoside resistance	Aminoglycoside
Aminoglycoside resistance	Aminoglycoside
Aminoglycoside resistance	Aminoglycoside
Aminoglycoside resistance	Aminoglycoside
Aminoglycoside resistance	Aminoglycoside
Aminoglycoside resistance	Aminoglycoside
Aminoglycoside resistance	Aminoglycoside
Aminoglycoside resistance	Aminoglycoside
Aminoglycoside resistance	Aminoglycoside

Aminoglycoside resistance	Aminoglycoside
Aminoglycoside resistance	Aminoglycoside
Aminoglycoside resistance	Aminoglycoside
Aminoglycoside resistance	Aminoglycoside
Aminoglycoside resistance	Aminoglycoside
Aminoglycoside resistance	Aminoglycoside
Aminoglycoside resistance	Aminoglycoside
Aminoglycoside resistance	Aminoglycoside
Aminoglycoside resistance	Aminoglycoside
Aminoglycoside resistance	Aminoglycoside
Aminoglycoside resistance	Aminoglycoside
Aminoglycoside resistance	Aminoglycoside
Aminoglycoside resistance	Aminoglycoside
Aminoglycoside resistance	Aminoglycoside
Aminoglycoside resistance	Aminoglycoside
Aminoglycoside resistance	Aminoglycoside
Aminoglycoside resistance	Aminoglycoside
Aminoglycoside resistance	Aminoglycoside
Aminoglycoside resistance	Aminoglycoside
Aminoglycoside resistance	Aminoglycoside
Aminoglycoside resistance	Aminoglycoside
Aminoglycoside resistance	Aminoglycoside
Aminoglycoside resistance	Aminoglycoside
Aminoglycoside resistance	Aminoglycoside
Aminoglycoside resistance	Aminoglycoside
Aminoglycoside resistance	Aminoglycoside
Aminoglycoside resistance	Aminoglycoside
Aminoglycoside resistance	Aminoglycoside
Aminoglycoside resistance	Aminoglycoside
Aminoglycoside resistance	Aminoglycoside
Aminoglycoside resistance	Aminoglycoside
Aminoglycoside resistance	Aminoglycoside
Aminoglycoside resistance	Aminoglycoside
Aminoglycoside resistance	Aminoglycoside
Aminoglycoside resistance	Aminoglycoside
Aminoglycoside resistance	Aminoglycoside
Aminoglycoside resistance	Aminoglycoside
Aminoglycoside resistance	Aminoglycoside
Aminoglycoside resistance	Aminoglycoside
Aminoglycoside resistance	Aminoglycoside
Aminoglycoside resistance	Aminoglycoside
Aminoglycoside resistance	Aminoglycoside
Aminoglycoside resistance	Aminoglycoside
Aminoglycoside resistance	Aminoglycoside

Aminoglycoside resistance	Aminoglycoside
Aminoglycoside resistance	Aminoglycoside
Aminoglycoside resistance	Aminoglycoside
Aminoglycoside resistance	Aminoglycoside
Aminoglycoside resistance	Aminoglycoside
Aminoglycoside resistance	Aminoglycoside
Aminoglycoside resistance	Aminoglycoside
Aminoglycoside resistance	Aminoglycoside
Aminoglycoside resistance	Aminoglycoside
Aminoglycoside resistance	Aminoglycoside
Aminoglycoside resistance	Aminoglycoside
Aminoglycoside resistance	Aminoglycoside
Aminoglycoside resistance	Aminoglycoside
Aminoglycoside resistance	Aminoglycoside
Aminoglycoside resistance	Aminoglycoside
Aminoglycoside resistance	Aminoglycoside
Aminoglycoside resistance	Aminoglycoside
Aminoglycoside resistance	Aminoglycoside
Aminoglycoside resistance	Aminoglycoside
Aminoglycoside resistance	Aminoglycoside
Aminoglycoside resistance	Aminoglycoside
Aminoglycoside resistance	Aminoglycoside
Aminoglycoside resistance	Aminoglycoside
Aminoglycoside resistance	Aminoglycoside
Aminoglycoside resistance	Aminoglycoside
Aminoglycoside resistance	Aminoglycoside
Aminoglycoside resistance	Aminoglycoside
Aminoglycoside resistance	Aminoglycoside
Aminoglycoside resistance	Aminoglycoside
Aminoglycoside resistance	Aminoglycoside
Aminoglycoside resistance	Aminoglycoside
Aminoglycoside resistance	Aminoglycoside
Aminoglycoside resistance	Aminoglycoside
Beta-lactam resistance	Beta-lactam
Beta-lactam resistance	Beta-lactam
Beta-lactam resistance	Beta-lactam
Beta-lactam resistance	Beta-lactam
Beta-lactam resistance	Beta-lactam
Beta-lactam resistance	Beta-lactam
Beta-lactam resistance	Beta-lactam
Beta-lactam resistance	Beta-lactam
Beta-lactam resistance	Beta-lactam
Beta-lactam resistance	Beta-lactam
Beta-lactam resistance	Beta-lactam

Beta-lactam resistance	Beta-lactam
Beta-lactam resistance	Beta-lactam
Beta-lactam resistance	Beta-lactam
Beta-lactam resistance	Beta-lactam
Beta-lactam resistance	Beta-lactam
Beta-lactam resistance	Beta-lactam
Beta-lactam resistance	Beta-lactam
Beta-lactam resistance	Beta-lactam
Beta-lactam resistance	Beta-lactam
Beta-lactam resistance	Beta-lactam
Beta-lactam resistance	Beta-lactam
Beta-lactam resistance	Beta-lactam
Beta-lactam resistance	Beta-lactam
Beta-lactam resistance	Beta-lactam
Beta-lactam resistance	Beta-lactam
Beta-lactam resistance	Beta-lactam
Beta-lactam resistance	Beta-lactam
Beta-lactam resistance	Beta-lactam
Beta-lactam resistance	Beta-lactam
Beta-lactam resistance	Beta-lactam
Beta-lactam resistance	Beta-lactam
Beta-lactam resistance	Beta-lactam
Beta-lactam resistance	Beta-lactam
Beta-lactam resistance	Beta-lactam
Beta-lactam resistance	Beta-lactam
Beta-lactam resistance	Beta-lactam
Beta-lactam resistance	Beta-lactam
Beta-lactam resistance	Beta-lactam
Beta-lactam resistance	Beta-lactam
Beta-lactam resistance	Beta-lactam
Beta-lactam resistance	Beta-lactam
Beta-lactam resistance	Beta-lactam
Beta-lactam resistance	Beta-lactam
Beta-lactam resistance	Beta-lactam
Beta-lactam resistance	Beta-lactam
Beta-lactam resistance	Beta-lactam
Beta-lactam resistance	Beta-lactam
Beta-lactam resistance	Beta-lactam
Beta-lactam resistance	Beta-lactam
Beta-lactam resistance	Beta-lactam
Beta-lactam resistance	Beta-lactam
Beta-lactam resistance	Beta-lactam
Beta-lactam resistance	Beta-lactam
Beta-lactam resistance	Beta-lactam

Beta-lactam resistance	Beta-lactam
Beta-lactam resistance	Beta-lactam
Beta-lactam resistance	Beta-lactam
Beta-lactam resistance	Beta-lactam
Beta-lactam resistance	Beta-lactam
Beta-lactam resistance	Beta-lactam
Beta-lactam resistance	Beta-lactam
Beta-lactam resistance	Beta-lactam
Beta-lactam resistance	Beta-lactam
Beta-lactam resistance	Beta-lactam
Beta-lactam resistance	Beta-lactam
Beta-lactam resistance	Beta-lactam
Beta-lactam resistance	Beta-lactam
Beta-lactam resistance	Beta-lactam
Beta-lactam resistance	Beta-lactam
Beta-lactam resistance	Beta-lactam
Beta-lactam resistance	Beta-lactam
Beta-lactam resistance	Beta-lactam
Beta-lactam resistance	Beta-lactam
Beta-lactam resistance	Beta-lactam
Beta-lactam resistance	Beta-lactam
Beta-lactam resistance	Beta-lactam
Beta-lactam resistance	Beta-lactam
Beta-lactam resistance	Beta-lactam
Beta-lactam resistance	Beta-lactam
Beta-lactam resistance	Beta-lactam
Beta-lactam resistance	Beta-lactam
Beta-lactam resistance	Beta-lactam
Beta-lactam resistance	Beta-lactam
Beta-lactam resistance	Beta-lactam
Beta-lactam resistance	Beta-lactam
Beta-lactam resistance	Beta-lactam
Beta-lactam resistance	Beta-lactam
Beta-lactam resistance	Beta-lactam
Beta-lactam resistance	Beta-lactam
Beta-lactam resistance	Beta-lactam
Beta-lactam resistance	Beta-lactam
Beta-lactam resistance	Beta-lactam
Beta-lactam resistance	Beta-lactam
Beta-lactam resistance	Beta-lactam
Beta-lactam resistance	Beta-lactam
Beta-lactam resistance	Beta-lactam
Beta-lactam resistance	Beta-lactam
Beta-lactam resistance	Beta-lactam

Beta-lactam resistance	Beta-lactam
Beta-lactam resistance	Beta-lactam
Beta-lactam resistance	Beta-lactam
Beta-lactam resistance	Beta-lactam
Beta-lactam resistance	Beta-lactam
Beta-lactam resistance	Beta-lactam
Beta-lactam resistance	Beta-lactam
Beta-lactam resistance	Beta-lactam
Beta-lactam resistance	Beta-lactam
Beta-lactam resistance	Beta-lactam
Beta-lactam resistance	Beta-lactam
Beta-lactam resistance	Beta-lactam
Beta-lactam resistance	Beta-lactam
Beta-lactam resistance	Beta-lactam
Beta-lactam resistance	Beta-lactam
Beta-lactam resistance	Beta-lactam
Beta-lactam resistance	Beta-lactam
Beta-lactam resistance	Beta-lactam
Beta-lactam resistance	Beta-lactam
Beta-lactam resistance	Beta-lactam
Beta-lactam resistance	Beta-lactam
Beta-lactam resistance	Beta-lactam
Beta-lactam resistance	Beta-lactam
Beta-lactam resistance	Beta-lactam
Beta-lactam resistance	Beta-lactam
Beta-lactam resistance	Beta-lactam
Beta-lactam resistance	Beta-lactam
Beta-lactam resistance	Beta-lactam
Beta-lactam resistance	Beta-lactam
Beta-lactam resistance	Beta-lactam
Beta-lactam resistance	Beta-lactam
Beta-lactam resistance	Beta-lactam
Beta-lactam resistance	Beta-lactam
Beta-lactam resistance	Beta-lactam
Beta-lactam resistance	Beta-lactam
Beta-lactam resistance	Beta-lactam
Beta-lactam resistance	Beta-lactam
Beta-lactam resistance	Beta-lactam
Beta-lactam resistance	Beta-lactam
Beta-lactam resistance	Beta-lactam
Beta-lactam resistance	Beta-lactam
Beta-lactam resistance	Beta-lactam
Beta-lactam resistance	Beta-lactam
Beta-lactam resistance	Beta-lactam

Beta-lactam resistance	Beta-lactam
Beta-lactam resistance	Beta-lactam
Beta-lactam resistance	Beta-lactam
Beta-lactam resistance	Beta-lactam
Beta-lactam resistance	Beta-lactam
Beta-lactam resistance	Beta-lactam
Beta-lactam resistance	Beta-lactam
Beta-lactam resistance	Beta-lactam
Beta-lactam resistance	Beta-lactam
Beta-lactam resistance	Beta-lactam
Beta-lactam resistance	Beta-lactam
Beta-lactam resistance	Beta-lactam
Beta-lactam resistance	Beta-lactam
Beta-lactam resistance	Beta-lactam
Beta-lactam resistance	Beta-lactam
Beta-lactam resistance	Beta-lactam
Beta-lactam resistance	Beta-lactam
Beta-lactam resistance	Beta-lactam
Beta-lactam resistance	Beta-lactam
Beta-lactam resistance	Beta-lactam
Beta-lactam resistance	Beta-lactam
Beta-lactam resistance	Beta-lactam
Beta-lactam resistance	Beta-lactam
Beta-lactam resistance	Beta-lactam
Beta-lactam resistance	Beta-lactam
Beta-lactam resistance	Beta-lactam
Beta-lactam resistance	Beta-lactam
Beta-lactam resistance	Beta-lactam
Beta-lactam resistance	Beta-lactam
Beta-lactam resistance	Beta-lactam
Beta-lactam resistance	Beta-lactam
Beta-lactam resistance	Beta-lactam
Beta-lactam resistance	Beta-lactam
Beta-lactam resistance	Beta-lactam
Beta-lactam resistance	Beta-lactam
Beta-lactam resistance	Beta-lactam
Beta-lactam resistance	Beta-lactam
Beta-lactam resistance	Beta-lactam
Beta-lactam resistance	Beta-lactam
Beta-lactam resistance	Beta-lactam
Beta-lactam resistance	Beta-lactam
Beta-lactam resistance	Beta-lactam
Beta-lactam resistance	Beta-lactam
Beta-lactam resistance	Beta-lactam

Beta-lactam resistance	Beta-lactam
Beta-lactam resistance	Beta-lactam
Beta-lactam resistance	Beta-lactam
Beta-lactam resistance	Beta-lactam
Beta-lactam resistance	Beta-lactam
Beta-lactam resistance	Beta-lactam
Beta-lactam resistance	Beta-lactam
Beta-lactam resistance	Beta-lactam
Beta-lactam resistance	Beta-lactam
Beta-lactam resistance	Beta-lactam
Beta-lactam resistance	Beta-lactam
Beta-lactam resistance	Beta-lactam
Beta-lactam resistance	Beta-lactam
Beta-lactam resistance	Beta-lactam
Beta-lactam resistance	Beta-lactam
Beta-lactam resistance	Beta-lactam
Beta-lactam resistance	Beta-lactam
Beta-lactam resistance	Beta-lactam
Beta-lactam resistance	Beta-lactam
Beta-lactam resistance	Beta-lactam
Beta-lactam resistance	Beta-lactam
Beta-lactam resistance	Beta-lactam
Beta-lactam resistance	Beta-lactam
Beta-lactam resistance	Beta-lactam
Beta-lactam resistance	Beta-lactam
Beta-lactam resistance	Beta-lactam
Beta-lactam resistance	Beta-lactam
Beta-lactam resistance	Beta-lactam
Beta-lactam resistance	Beta-lactam
Beta-lactam resistance	Beta-lactam
Beta-lactam resistance	Beta-lactam
Beta-lactam resistance	Beta-lactam
Beta-lactam resistance	Beta-lactam
Beta-lactam resistance	Beta-lactam
Beta-lactam resistance	Beta-lactam
Beta-lactam resistance	Beta-lactam
Beta-lactam resistance	Beta-lactam
Beta-lactam resistance	Beta-lactam
Beta-lactam resistance	Beta-lactam
Beta-lactam resistance	Beta-lactam
Beta-lactam resistance	Beta-lactam
Beta-lactam resistance	Beta-lactam
Beta-lactam resistance	Beta-lactam
Beta-lactam resistance	Beta-lactam

Beta-lactam resistance	Beta-lactam
Beta-lactam resistance	Beta-lactam
Beta-lactam resistance	Beta-lactam
Beta-lactam resistance	Beta-lactam
Beta-lactam resistance	Beta-lactam
Beta-lactam resistance	Beta-lactam
Beta-lactam resistance	Beta-lactam
Beta-lactam resistance	Beta-lactam
Beta-lactam resistance	Beta-lactam
Beta-lactam resistance	Beta-lactam
Beta-lactam resistance	Beta-lactam
Beta-lactam resistance	Beta-lactam
Beta-lactam resistance	Beta-lactam
Beta-lactam resistance	Beta-lactam
Beta-lactam resistance	Beta-lactam
Beta-lactam resistance	Beta-lactam
Beta-lactam resistance	Beta-lactam
Beta-lactam resistance	Beta-lactam
Beta-lactam resistance	Beta-lactam
Beta-lactam resistance	Beta-lactam
Beta-lactam resistance	Beta-lactam
Beta-lactam resistance	Beta-lactam
Beta-lactam resistance	Beta-lactam
Beta-lactam resistance	Beta-lactam
Beta-lactam resistance	Beta-lactam
Beta-lactam resistance	Beta-lactam
Beta-lactam resistance	Beta-lactam
Beta-lactam resistance	Beta-lactam
Beta-lactam resistance	Beta-lactam
Beta-lactam resistance	Beta-lactam
Beta-lactam resistance	Beta-lactam
Beta-lactam resistance	Beta-lactam
Beta-lactam resistance	Beta-lactam
Beta-lactam resistance	Beta-lactam
Beta-lactam resistance	Beta-lactam
Beta-lactam resistance	Beta-lactam
Beta-lactam resistance	Beta-lactam
Beta-lactam resistance	Beta-lactam
Beta-lactam resistance	Beta-lactam
Beta-lactam resistance	Beta-lactam
Beta-lactam resistance	Beta-lactam
Beta-lactam resistance	Beta-lactam
Beta-lactam resistance	Beta-lactam
Beta-lactam resistance	Beta-lactam

Beta-lactam resistance	Beta-lactam
Beta-lactam resistance	Beta-lactam
Beta-lactam resistance	Beta-lactam
Beta-lactam resistance	Beta-lactam
Beta-lactam resistance	Beta-lactam
Beta-lactam resistance	Beta-lactam
Beta-lactam resistance	Beta-lactam
Beta-lactam resistance	Beta-lactam
Beta-lactam resistance	Beta-lactam
Beta-lactam resistance	Beta-lactam
Beta-lactam resistance	Beta-lactam
Beta-lactam resistance	Beta-lactam
Beta-lactam resistance	Beta-lactam
Beta-lactam resistance	Beta-lactam
Beta-lactam resistance	Beta-lactam
Beta-lactam resistance	Beta-lactam
Beta-lactam resistance	Beta-lactam
Beta-lactam resistance	Beta-lactam
Beta-lactam resistance	Beta-lactam
Beta-lactam resistance	Beta-lactam
Beta-lactam resistance	Beta-lactam
Beta-lactam resistance	Beta-lactam
Beta-lactam resistance	Beta-lactam
Beta-lactam resistance	Beta-lactam
Beta-lactam resistance	Beta-lactam
Beta-lactam resistance	Beta-lactam
Beta-lactam resistance	Beta-lactam
Beta-lactam resistance	Beta-lactam
Beta-lactam resistance	Beta-lactam
Beta-lactam resistance	Beta-lactam
Beta-lactam resistance	Beta-lactam
Beta-lactam resistance	Beta-lactam
Beta-lactam resistance	Beta-lactam
Beta-lactam resistance	Beta-lactam
Beta-lactam resistance	Beta-lactam
Beta-lactam resistance	Beta-lactam
Beta-lactam resistance	Beta-lactam
Beta-lactam resistance	Beta-lactam
Beta-lactam resistance	Beta-lactam
Beta-lactam resistance	Beta-lactam
Beta-lactam resistance	Beta-lactam
Beta-lactam resistance	Beta-lactam
Beta-lactam resistance	Beta-lactam
Beta-lactam resistance	Beta-lactam

Beta-lactam resistance	Beta-lactam
Beta-lactam resistance	Beta-lactam
Beta-lactam resistance	Beta-lactam
Beta-lactam resistance	Beta-lactam
Beta-lactam resistance	Beta-lactam
Beta-lactam resistance	Beta-lactam
Beta-lactam resistance	Beta-lactam
Beta-lactam resistance	Beta-lactam
Beta-lactam resistance	Beta-lactam
Beta-lactam resistance	Beta-lactam
Beta-lactam resistance	Beta-lactam
Beta-lactam resistance	Beta-lactam
Beta-lactam resistance	Beta-lactam
Beta-lactam resistance	Beta-lactam
Beta-lactam resistance	Beta-lactam
Beta-lactam resistance	Beta-lactam
Beta-lactam resistance	Beta-lactam
Beta-lactam resistance	Beta-lactam
Beta-lactam resistance	Beta-lactam
Beta-lactam resistance	Beta-lactam
Beta-lactam resistance	Beta-lactam
Beta-lactam resistance	Beta-lactam
Beta-lactam resistance	Beta-lactam
Beta-lactam resistance	Beta-lactam
Beta-lactam resistance	Beta-lactam
Beta-lactam resistance	Beta-lactam
Beta-lactam resistance	Beta-lactam
Beta-lactam resistance	Beta-lactam
Beta-lactam resistance	Beta-lactam
Beta-lactam resistance	Beta-lactam
Beta-lactam resistance	Beta-lactam
Beta-lactam resistance	Beta-lactam
Beta-lactam resistance	Beta-lactam
Beta-lactam resistance	Beta-lactam
Beta-lactam resistance	Beta-lactam
Beta-lactam resistance	Beta-lactam
Beta-lactam resistance	Beta-lactam
Beta-lactam resistance	Beta-lactam
Beta-lactam resistance	Beta-lactam
Beta-lactam resistance	Beta-lactam
Beta-lactam resistance	Beta-lactam
Beta-lactam resistance	Beta-lactam
Beta-lactam resistance	Beta-lactam
Beta-lactam resistance	Beta-lactam

Beta-lactam resistance	Beta-lactam
Beta-lactam resistance	Beta-lactam
Beta-lactam resistance	Beta-lactam
Beta-lactam resistance	Beta-lactam
Beta-lactam resistance	Beta-lactam
Beta-lactam resistance	Beta-lactam
Beta-lactam resistance	Beta-lactam
Beta-lactam resistance	Beta-lactam
Beta-lactam resistance	Beta-lactam
Beta-lactam resistance	Beta-lactam
Beta-lactam resistance	Beta-lactam
Beta-lactam resistance	Beta-lactam
Beta-lactam resistance	Beta-lactam
Beta-lactam resistance	Beta-lactam
Beta-lactam resistance	Beta-lactam
Beta-lactam resistance	Beta-lactam
Beta-lactam resistance	Beta-lactam
Beta-lactam resistance	Beta-lactam
Beta-lactam resistance	Beta-lactam
Beta-lactam resistance	Beta-lactam
Beta-lactam resistance	Beta-lactam
Beta-lactam resistance	Beta-lactam
Beta-lactam resistance	Beta-lactam
Beta-lactam resistance	Beta-lactam
Beta-lactam resistance	Beta-lactam
Beta-lactam resistance	Beta-lactam
Beta-lactam resistance	Beta-lactam
Beta-lactam resistance	Beta-lactam
Beta-lactam resistance	Beta-lactam
Beta-lactam resistance	Beta-lactam
Beta-lactam resistance	Beta-lactam
Beta-lactam resistance	Beta-lactam
Beta-lactam resistance	Beta-lactam
Beta-lactam resistance	Beta-lactam
Beta-lactam resistance	Beta-lactam
Beta-lactam resistance	Beta-lactam
Beta-lactam resistance	Beta-lactam
Beta-lactam resistance	Beta-lactam
Beta-lactam resistance	Beta-lactam
Beta-lactam resistance	Beta-lactam
Beta-lactam resistance	Beta-lactam
Beta-lactam resistance	Beta-lactam
Beta-lactam resistance	Beta-lactam
Beta-lactam resistance	Beta-lactam

Beta-lactam resistance	Beta-lactam
Beta-lactam resistance	Beta-lactam
Beta-lactam resistance	Beta-lactam
Beta-lactam resistance	Beta-lactam
Beta-lactam resistance	Beta-lactam
Beta-lactam resistance	Beta-lactam
Beta-lactam resistance	Beta-lactam
Beta-lactam resistance	Beta-lactam
Beta-lactam resistance	Beta-lactam
Beta-lactam resistance	Beta-lactam
Beta-lactam resistance	Beta-lactam
Beta-lactam resistance	Beta-lactam
Beta-lactam resistance	Beta-lactam
Beta-lactam resistance	Beta-lactam
Beta-lactam resistance	Beta-lactam
Beta-lactam resistance	Beta-lactam
Beta-lactam resistance	Beta-lactam
Beta-lactam resistance	Beta-lactam
Beta-lactam resistance	Beta-lactam
Beta-lactam resistance	Beta-lactam
Beta-lactam resistance	Beta-lactam
Beta-lactam resistance	Beta-lactam
Beta-lactam resistance	Beta-lactam
Beta-lactam resistance	Beta-lactam
Beta-lactam resistance	Beta-lactam
Beta-lactam resistance	Beta-lactam
Beta-lactam resistance	Beta-lactam
Beta-lactam resistance	Beta-lactam
Beta-lactam resistance	Beta-lactam
Beta-lactam resistance	Beta-lactam
Beta-lactam resistance	Beta-lactam
Beta-lactam resistance	Beta-lactam
Beta-lactam resistance	Beta-lactam
Beta-lactam resistance	Beta-lactam
Beta-lactam resistance	Beta-lactam
Beta-lactam resistance	Beta-lactam
Beta-lactam resistance	Beta-lactam
Beta-lactam resistance	Beta-lactam
Beta-lactam resistance	Beta-lactam
Beta-lactam resistance	Beta-lactam
Beta-lactam resistance	Beta-lactam
Beta-lactam resistance	Beta-lactam
Beta-lactam resistance	Beta-lactam
Beta-lactam resistance	Beta-lactam

Beta-lactam resistance	Beta-lactam
Beta-lactam resistance	Beta-lactam
Beta-lactam resistance	Beta-lactam
Beta-lactam resistance	Beta-lactam
Beta-lactam resistance	Beta-lactam
Beta-lactam resistance	Beta-lactam
Beta-lactam resistance	Beta-lactam
Beta-lactam resistance	Beta-lactam
Beta-lactam resistance	Beta-lactam
Beta-lactam resistance	Beta-lactam
Beta-lactam resistance	Beta-lactam
Beta-lactam resistance	Beta-lactam
Beta-lactam resistance	Beta-lactam
Beta-lactam resistance	Beta-lactam
Beta-lactam resistance	Beta-lactam
Beta-lactam resistance	Beta-lactam
Beta-lactam resistance	Beta-lactam
Beta-lactam resistance	Beta-lactam
Beta-lactam resistance	Beta-lactam
Beta-lactam resistance	Beta-lactam
Beta-lactam resistance	Beta-lactam
Beta-lactam resistance	Beta-lactam
Beta-lactam resistance	Beta-lactam
Beta-lactam resistance	Beta-lactam
Beta-lactam resistance	Beta-lactam
Beta-lactam resistance	Beta-lactam
Beta-lactam resistance	Beta-lactam
Beta-lactam resistance	Beta-lactam
Beta-lactam resistance	Beta-lactam
Beta-lactam resistance	Beta-lactam
Beta-lactam resistance	Beta-lactam
Beta-lactam resistance	Beta-lactam
Beta-lactam resistance	Beta-lactam
Beta-lactam resistance	Beta-lactam
Beta-lactam resistance	Beta-lactam
Beta-lactam resistance	Beta-lactam
Beta-lactam resistance	Beta-lactam
Beta-lactam resistance	Beta-lactam
Beta-lactam resistance	Beta-lactam
Beta-lactam resistance	Beta-lactam
Beta-lactam resistance	Beta-lactam
Beta-lactam resistance	Beta-lactam
Beta-lactam resistance	Beta-lactam
Beta-lactam resistance	Beta-lactam

Beta-lactam resistance	Beta-lactam
Beta-lactam resistance	Beta-lactam
Beta-lactam resistance	Beta-lactam
Beta-lactam resistance	Beta-lactam
Beta-lactam resistance	Beta-lactam
Beta-lactam resistance	Beta-lactam
Beta-lactam resistance	Beta-lactam
Beta-lactam resistance	Beta-lactam
Beta-lactam resistance	Beta-lactam
Beta-lactam resistance	Beta-lactam
Beta-lactam resistance	Beta-lactam
Beta-lactam resistance	Beta-lactam
Beta-lactam resistance	Beta-lactam
Beta-lactam resistance	Beta-lactam
Beta-lactam resistance	Beta-lactam
Beta-lactam resistance	Beta-lactam
Beta-lactam resistance	Beta-lactam
Beta-lactam resistance	Beta-lactam
Beta-lactam resistance	Beta-lactam
Beta-lactam resistance	Beta-lactam
Beta-lactam resistance	Beta-lactam
Beta-lactam resistance	Beta-lactam
Beta-lactam resistance	Beta-lactam
Beta-lactam resistance	Beta-lactam
Beta-lactam resistance	Beta-lactam
Beta-lactam resistance	Beta-lactam
Beta-lactam resistance	Beta-lactam
Beta-lactam resistance	Beta-lactam
Beta-lactam resistance	Beta-lactam
Beta-lactam resistance	Beta-lactam
Beta-lactam resistance	Beta-lactam
Beta-lactam resistance	Beta-lactam
Beta-lactam resistance	Beta-lactam
Beta-lactam resistance	Beta-lactam
Beta-lactam resistance	Beta-lactam
Beta-lactam resistance	Beta-lactam
Beta-lactam resistance	Beta-lactam
Beta-lactam resistance	Beta-lactam
Beta-lactam resistance	Beta-lactam
Beta-lactam resistance	Beta-lactam
Beta-lactam resistance	Beta-lactam
Beta-lactam resistance	Beta-lactam
Beta-lactam resistance	Beta-lactam
Beta-lactam resistance	Beta-lactam

Beta-lactam resistance	Beta-lactam
Beta-lactam resistance	Beta-lactam
Beta-lactam resistance	Beta-lactam
Beta-lactam resistance	Beta-lactam
Beta-lactam resistance	Beta-lactam
Beta-lactam resistance	Beta-lactam
Beta-lactam resistance	Beta-lactam
Beta-lactam resistance	Beta-lactam
Beta-lactam resistance	Beta-lactam
Beta-lactam resistance	Beta-lactam
Beta-lactam resistance	Beta-lactam
Beta-lactam resistance	Beta-lactam
Beta-lactam resistance	Beta-lactam
Beta-lactam resistance	Beta-lactam
Beta-lactam resistance	Beta-lactam
Beta-lactam resistance	Beta-lactam
Beta-lactam resistance	Beta-lactam
Beta-lactam resistance	Beta-lactam
Beta-lactam resistance	Beta-lactam
Beta-lactam resistance	Beta-lactam
Beta-lactam resistance	Beta-lactam
Beta-lactam resistance	Beta-lactam
Beta-lactam resistance	Beta-lactam
Beta-lactam resistance	Beta-lactam
Beta-lactam resistance	Beta-lactam
Beta-lactam resistance	Beta-lactam
Beta-lactam resistance	Beta-lactam
Beta-lactam resistance	Beta-lactam
Beta-lactam resistance	Beta-lactam
Beta-lactam resistance	Beta-lactam
Beta-lactam resistance	Beta-lactam
Beta-lactam resistance	Beta-lactam
Beta-lactam resistance	Beta-lactam
Beta-lactam resistance	Beta-lactam
Beta-lactam resistance	Beta-lactam
Beta-lactam resistance	Beta-lactam
Beta-lactam resistance	Beta-lactam
Beta-lactam resistance	Beta-lactam
Beta-lactam resistance	Beta-lactam
Beta-lactam resistance	Beta-lactam
Beta-lactam resistance	Beta-lactam
Beta-lactam resistance	Beta-lactam
Beta-lactam resistance	Beta-lactam
Beta-lactam resistance	Beta-lactam

Beta-lactam resistance	Beta-lactam
Beta-lactam resistance	Beta-lactam
Beta-lactam resistance	Beta-lactam
Beta-lactam resistance	Beta-lactam
Beta-lactam resistance	Beta-lactam
Beta-lactam resistance	Beta-lactam
Beta-lactam resistance	Beta-lactam
Beta-lactam resistance	Beta-lactam
Beta-lactam resistance	Beta-lactam
Beta-lactam resistance	Beta-lactam
Beta-lactam resistance	Beta-lactam
Beta-lactam resistance	Beta-lactam
Beta-lactam resistance	Beta-lactam
Beta-lactam resistance	Beta-lactam
Beta-lactam resistance	Beta-lactam
Beta-lactam resistance	Beta-lactam
Beta-lactam resistance	Beta-lactam
Beta-lactam resistance	Beta-lactam
Beta-lactam resistance	Beta-lactam
Beta-lactam resistance	Beta-lactam
Beta-lactam resistance	Beta-lactam
Beta-lactam resistance	Beta-lactam
Beta-lactam resistance	Beta-lactam
Beta-lactam resistance	Beta-lactam
Beta-lactam resistance	Beta-lactam
Beta-lactam resistance	Beta-lactam
Beta-lactam resistance	Beta-lactam
Beta-lactam resistance	Beta-lactam
Beta-lactam resistance	Beta-lactam
Beta-lactam resistance	Beta-lactam
Beta-lactam resistance	Beta-lactam
Beta-lactam resistance	Beta-lactam
Beta-lactam resistance	Beta-lactam
Beta-lactam resistance	Beta-lactam
Beta-lactam resistance	Beta-lactam
Beta-lactam resistance	Beta-lactam
Beta-lactam resistance	Beta-lactam
Beta-lactam resistance	Beta-lactam
Beta-lactam resistance	Beta-lactam
Beta-lactam resistance	Beta-lactam
Beta-lactam resistance	Beta-lactam
Beta-lactam resistance	Beta-lactam
Beta-lactam resistance	Beta-lactam
Beta-lactam resistance	Beta-lactam

Beta-lactam resistance	Beta-lactam
Beta-lactam resistance	Beta-lactam
Beta-lactam resistance	Beta-lactam
Beta-lactam resistance	Beta-lactam
Beta-lactam resistance	Beta-lactam
Beta-lactam resistance	Beta-lactam
Beta-lactam resistance	Beta-lactam
Beta-lactam resistance	Beta-lactam
Beta-lactam resistance	Beta-lactam
Beta-lactam resistance	Beta-lactam
Beta-lactam resistance	Beta-lactam
Beta-lactam resistance	Beta-lactam
Beta-lactam resistance	Beta-lactam
Beta-lactam resistance	Beta-lactam
Beta-lactam resistance	Beta-lactam
Beta-lactam resistance	Beta-lactam
Beta-lactam resistance	Beta-lactam
Beta-lactam resistance	Beta-lactam
Beta-lactam resistance	Beta-lactam
Beta-lactam resistance	Beta-lactam
Beta-lactam resistance	Beta-lactam
Beta-lactam resistance	Beta-lactam
Beta-lactam resistance	Beta-lactam
Beta-lactam resistance	Beta-lactam
Beta-lactam resistance	Beta-lactam
Beta-lactam resistance	Beta-lactam
Beta-lactam resistance	Beta-lactam
Beta-lactam resistance	Beta-lactam
Beta-lactam resistance	Beta-lactam
Beta-lactam resistance	Beta-lactam
Beta-lactam resistance	Beta-lactam
Beta-lactam resistance	Beta-lactam
Beta-lactam resistance	Beta-lactam
Beta-lactam resistance	Beta-lactam
Beta-lactam resistance	Beta-lactam
Beta-lactam resistance	Beta-lactam
Beta-lactam resistance	Beta-lactam
Beta-lactam resistance	Beta-lactam
Beta-lactam resistance	Beta-lactam
Beta-lactam resistance	Beta-lactam
Beta-lactam resistance	Beta-lactam
Beta-lactam resistance	Beta-lactam
Beta-lactam resistance	Beta-lactam
Beta-lactam resistance	Beta-lactam

Beta-lactam resistance	Beta-lactam
Beta-lactam resistance	Beta-lactam
Beta-lactam resistance	Beta-lactam
Beta-lactam resistance	Beta-lactam
Beta-lactam resistance	Beta-lactam
Beta-lactam resistance	Beta-lactam
Beta-lactam resistance	Beta-lactam
Beta-lactam resistance	Beta-lactam
Beta-lactam resistance	Beta-lactam
Beta-lactam resistance	Beta-lactam
Beta-lactam resistance	Beta-lactam
Beta-lactam resistance	Beta-lactam
Beta-lactam resistance	Beta-lactam
Beta-lactam resistance	Beta-lactam
Beta-lactam resistance	Beta-lactam
Beta-lactam resistance	Beta-lactam
Beta-lactam resistance	Beta-lactam
Beta-lactam resistance	Beta-lactam
Beta-lactam resistance	Beta-lactam
Beta-lactam resistance	Beta-lactam
Beta-lactam resistance	Beta-lactam
Beta-lactam resistance	Beta-lactam
Beta-lactam resistance	Beta-lactam
Beta-lactam resistance	Beta-lactam
Beta-lactam resistance	Beta-lactam
Beta-lactam resistance	Beta-lactam
Beta-lactam resistance	Beta-lactam
Beta-lactam resistance	Beta-lactam
Beta-lactam resistance	Beta-lactam
Beta-lactam resistance	Beta-lactam
Beta-lactam resistance	Beta-lactam
Beta-lactam resistance	Beta-lactam
Beta-lactam resistance	Beta-lactam
Beta-lactam resistance	Beta-lactam
Beta-lactam resistance	Beta-lactam
Beta-lactam resistance	Beta-lactam
Beta-lactam resistance	Beta-lactam
Beta-lactam resistance	Beta-lactam
Beta-lactam resistance	Beta-lactam
Beta-lactam resistance	Beta-lactam
Beta-lactam resistance	Beta-lactam
Beta-lactam resistance	Beta-lactam
Beta-lactam resistance	Beta-lactam
Beta-lactam resistance	Beta-lactam

Beta-lactam resistance	Beta-lactam
Beta-lactam resistance	Beta-lactam
Beta-lactam resistance	Beta-lactam
Beta-lactam resistance	Beta-lactam
Beta-lactam resistance	Beta-lactam
Beta-lactam resistance	Beta-lactam
Beta-lactam resistance	Beta-lactam
Beta-lactam resistance	Beta-lactam
Beta-lactam resistance	Beta-lactam
Beta-lactam resistance	Beta-lactam
Beta-lactam resistance	Beta-lactam
Beta-lactam resistance	Beta-lactam
Beta-lactam resistance	Beta-lactam
Beta-lactam resistance	Beta-lactam
Beta-lactam resistance	Beta-lactam
Beta-lactam resistance	Beta-lactam
Beta-lactam resistance	Beta-lactam
Beta-lactam resistance	Beta-lactam
Beta-lactam resistance	Beta-lactam
Beta-lactam resistance	Beta-lactam
Beta-lactam resistance	Beta-lactam
Beta-lactam resistance	Beta-lactam
Beta-lactam resistance	Beta-lactam
Beta-lactam resistance	Beta-lactam
Beta-lactam resistance	Beta-lactam
Beta-lactam resistance	Beta-lactam
Beta-lactam resistance	Beta-lactam
Beta-lactam resistance	Beta-lactam
Beta-lactam resistance	Beta-lactam
Beta-lactam resistance	Beta-lactam
Beta-lactam resistance	Beta-lactam
Beta-lactam resistance	Beta-lactam
Beta-lactam resistance	Beta-lactam
Beta-lactam resistance	Beta-lactam
Beta-lactam resistance	Beta-lactam
Beta-lactam resistance	Beta-lactam
Beta-lactam resistance	Beta-lactam
Beta-lactam resistance	Beta-lactam
Beta-lactam resistance	Beta-lactam
Beta-lactam resistance	Beta-lactam
Beta-lactam resistance	Beta-lactam
Beta-lactam resistance	Beta-lactam
Beta-lactam resistance	Beta-lactam
Beta-lactam resistance	Beta-lactam

Beta-lactam resistance	Beta-lactam
Beta-lactam resistance	Beta-lactam
Beta-lactam resistance	Beta-lactam
Beta-lactam resistance	Beta-lactam
Beta-lactam resistance	Beta-lactam
Beta-lactam resistance	Beta-lactam
Beta-lactam resistance	Beta-lactam
Beta-lactam resistance	Beta-lactam
Beta-lactam resistance	Beta-lactam
Beta-lactam resistance	Beta-lactam
Beta-lactam resistance	Beta-lactam
Beta-lactam resistance	Beta-lactam
Beta-lactam resistance	Beta-lactam
Beta-lactam resistance	Beta-lactam
Beta-lactam resistance	Beta-lactam
Beta-lactam resistance	Beta-lactam
Beta-lactam resistance	Beta-lactam
Beta-lactam resistance	Beta-lactam
Beta-lactam resistance	Beta-lactam
Beta-lactam resistance	Beta-lactam
Beta-lactam resistance	Beta-lactam
Beta-lactam resistance	Beta-lactam
Beta-lactam resistance	Beta-lactam
Beta-lactam resistance	Beta-lactam
Beta-lactam resistance	Beta-lactam
Beta-lactam resistance	Beta-lactam
Beta-lactam resistance	Beta-lactam
Beta-lactam resistance	Beta-lactam
Beta-lactam resistance	Beta-lactam
Beta-lactam resistance	Beta-lactam
Beta-lactam resistance	Beta-lactam
Beta-lactam resistance	Beta-lactam
Beta-lactam resistance	Beta-lactam
Beta-lactam resistance	Beta-lactam
Beta-lactam resistance	Beta-lactam
Beta-lactam resistance	Beta-lactam
Beta-lactam resistance	Beta-lactam
Beta-lactam resistance	Beta-lactam
Beta-lactam resistance	Beta-lactam
Beta-lactam resistance	Beta-lactam
Beta-lactam resistance	Beta-lactam
Beta-lactam resistance	Beta-lactam
Beta-lactam resistance	Beta-lactam
Beta-lactam resistance	Beta-lactam

Beta-lactam resistance	Beta-lactam
Beta-lactam resistance	Beta-lactam
Beta-lactam resistance	Beta-lactam
Beta-lactam resistance	Beta-lactam
Beta-lactam resistance	Beta-lactam
Beta-lactam resistance	Beta-lactam
Beta-lactam resistance	Beta-lactam
Beta-lactam resistance	Beta-lactam
Beta-lactam resistance	Beta-lactam
Beta-lactam resistance	Beta-lactam
Beta-lactam resistance	Beta-lactam
Beta-lactam resistance	Beta-lactam
Beta-lactam resistance	Beta-lactam
Beta-lactam resistance	Beta-lactam
Beta-lactam resistance	Beta-lactam
Beta-lactam resistance	Beta-lactam
Beta-lactam resistance	Beta-lactam
Beta-lactam resistance	Beta-lactam
Beta-lactam resistance	Beta-lactam
Beta-lactam resistance	Beta-lactam
Beta-lactam resistance	Beta-lactam
Beta-lactam resistance	Beta-lactam
Beta-lactam resistance	Beta-lactam
Beta-lactam resistance	Beta-lactam
Beta-lactam resistance	Beta-lactam
Beta-lactam resistance	Beta-lactam
Beta-lactam resistance	Beta-lactam
Beta-lactam resistance	Beta-lactam
Beta-lactam resistance	Beta-lactam
Beta-lactam resistance	Beta-lactam
Beta-lactam resistance	Beta-lactam
Beta-lactam resistance	Beta-lactam
Beta-lactam resistance	Beta-lactam
Beta-lactam resistance	Beta-lactam
Beta-lactam resistance	Beta-lactam
Beta-lactam resistance	Beta-lactam
Beta-lactam resistance	Beta-lactam
Beta-lactam resistance	Beta-lactam
Beta-lactam resistance	Beta-lactam
Beta-lactam resistance	Beta-lactam
Beta-lactam resistance	Beta-lactam
Beta-lactam resistance	Beta-lactam
Beta-lactam resistance	Beta-lactam
Beta-lactam resistance	Beta-lactam

Beta-lactam resistance	Beta-lactam
Beta-lactam resistance	Beta-lactam
Beta-lactam resistance	Beta-lactam
Beta-lactam resistance	Beta-lactam
Beta-lactam resistance	Beta-lactam
Beta-lactam resistance	Beta-lactam
Beta-lactam resistance	Beta-lactam
Beta-lactam resistance	Beta-lactam
Beta-lactam resistance	Beta-lactam
Beta-lactam resistance	Beta-lactam
Beta-lactam resistance	Beta-lactam
Beta-lactam resistance	Beta-lactam
Beta-lactam resistance	Beta-lactam
Beta-lactam resistance	Beta-lactam
Beta-lactam resistance	Beta-lactam
Beta-lactam resistance	Beta-lactam
Beta-lactam resistance	Beta-lactam
Beta-lactam resistance	Beta-lactam
Beta-lactam resistance	Beta-lactam
Beta-lactam resistance	Beta-lactam
Beta-lactam resistance	Beta-lactam
Beta-lactam resistance	Beta-lactam
Beta-lactam resistance	Beta-lactam
Beta-lactam resistance	Beta-lactam
Beta-lactam resistance	Beta-lactam
Beta-lactam resistance	Beta-lactam
Beta-lactam resistance	Beta-lactam
Beta-lactam resistance	Beta-lactam
Beta-lactam resistance	Beta-lactam
Beta-lactam resistance	Beta-lactam
Beta-lactam resistance	Beta-lactam
Beta-lactam resistance	Beta-lactam
Beta-lactam resistance	Beta-lactam
Beta-lactam resistance	Beta-lactam
Beta-lactam resistance	Beta-lactam
Beta-lactam resistance	Beta-lactam
Beta-lactam resistance	Beta-lactam
Beta-lactam resistance	Beta-lactam
Beta-lactam resistance	Beta-lactam
Beta-lactam resistance	Beta-lactam
Beta-lactam resistance	Beta-lactam
Beta-lactam resistance	Beta-lactam
Beta-lactam resistance	Beta-lactam
Beta-lactam resistance	Beta-lactam

Beta-lactam resistance	Beta-lactam
Beta-lactam resistance	Beta-lactam
Beta-lactam resistance	Beta-lactam
Beta-lactam resistance	Beta-lactam
Beta-lactam resistance	Beta-lactam
Beta-lactam resistance	Beta-lactam
Beta-lactam resistance	Beta-lactam
Beta-lactam resistance	Beta-lactam
Beta-lactam resistance	Beta-lactam
Beta-lactam resistance	Beta-lactam
Beta-lactam resistance	Beta-lactam
Beta-lactam resistance	Beta-lactam
Beta-lactam resistance	Beta-lactam
Beta-lactam resistance	Beta-lactam
Beta-lactam resistance	Beta-lactam
Beta-lactam resistance	Beta-lactam
Beta-lactam resistance	Beta-lactam
Beta-lactam resistance	Beta-lactam
Beta-lactam resistance	Beta-lactam
Beta-lactam resistance	Beta-lactam
Beta-lactam resistance	Beta-lactam
Beta-lactam resistance	Beta-lactam
Beta-lactam resistance	Beta-lactam
Beta-lactam resistance	Beta-lactam
Beta-lactam resistance	Beta-lactam
Beta-lactam resistance	Beta-lactam
Beta-lactam resistance	Beta-lactam
Beta-lactam resistance	Beta-lactam
Beta-lactam resistance	Beta-lactam
Beta-lactam resistance	Beta-lactam
Beta-lactam resistance	Beta-lactam
Beta-lactam resistance	Beta-lactam
Beta-lactam resistance	Beta-lactam
Beta-lactam resistance	Beta-lactam
Beta-lactam resistance	Beta-lactam
Beta-lactam resistance	Beta-lactam
Beta-lactam resistance	Beta-lactam
Beta-lactam resistance	Beta-lactam
Beta-lactam resistance	Beta-lactam
Beta-lactam resistance	Beta-lactam
Beta-lactam resistance	Beta-lactam
Beta-lactam resistance	Beta-lactam
Beta-lactam resistance	Beta-lactam
Beta-lactam resistance	Beta-lactam

Beta-lactam resistance	Beta-lactam
Beta-lactam resistance	Beta-lactam
Beta-lactam resistance	Beta-lactam
Beta-lactam resistance	Beta-lactam
Beta-lactam resistance	Beta-lactam
Beta-lactam resistance	Beta-lactam
Beta-lactam resistance	Beta-lactam
Beta-lactam resistance	Beta-lactam
Beta-lactam resistance	Beta-lactam
Beta-lactam resistance	Beta-lactam
Beta-lactam resistance	Beta-lactam
Beta-lactam resistance	Beta-lactam
Beta-lactam resistance	Beta-lactam
Beta-lactam resistance	Beta-lactam
Beta-lactam resistance	Beta-lactam
Beta-lactam resistance	Beta-lactam
Beta-lactam resistance	Beta-lactam
Beta-lactam resistance	Beta-lactam
Beta-lactam resistance	Beta-lactam
Beta-lactam resistance	Beta-lactam
Beta-lactam resistance	Beta-lactam
Beta-lactam resistance	Beta-lactam
Beta-lactam resistance	Beta-lactam
Beta-lactam resistance	Beta-lactam
Beta-lactam resistance	Beta-lactam
Beta-lactam resistance	Beta-lactam
Beta-lactam resistance	Beta-lactam
Beta-lactam resistance	Beta-lactam
Beta-lactam resistance	Beta-lactam
Beta-lactam resistance	Beta-lactam
Beta-lactam resistance	Beta-lactam
Beta-lactam resistance	Beta-lactam
Beta-lactam resistance	Beta-lactam
Beta-lactam resistance	Beta-lactam
Beta-lactam resistance	Beta-lactam
Beta-lactam resistance	Beta-lactam
Beta-lactam resistance	Beta-lactam
Beta-lactam resistance	Beta-lactam
Beta-lactam resistance	Beta-lactam
Beta-lactam resistance	Beta-lactam
Beta-lactam resistance	Beta-lactam
Beta-lactam resistance	Beta-lactam
Beta-lactam resistance	Beta-lactam
Beta-lactam resistance	Beta-lactam

Beta-lactam resistance	Beta-lactam
Beta-lactam resistance	Beta-lactam
Beta-lactam resistance	Beta-lactam
Beta-lactam resistance	Beta-lactam
Beta-lactam resistance	Beta-lactam
Beta-lactam resistance	Beta-lactam
Beta-lactam resistance	Beta-lactam
Beta-lactam resistance	Beta-lactam
Beta-lactam resistance	Beta-lactam
Beta-lactam resistance	Beta-lactam
Beta-lactam resistance	Beta-lactam
Beta-lactam resistance	Beta-lactam
Beta-lactam resistance	Beta-lactam
Beta-lactam resistance	Beta-lactam
Beta-lactam resistance	Beta-lactam
Beta-lactam resistance	Beta-lactam
Beta-lactam resistance	Beta-lactam
Beta-lactam resistance	Beta-lactam
Beta-lactam resistance	Beta-lactam
Beta-lactam resistance	Beta-lactam
Beta-lactam resistance	Beta-lactam
Beta-lactam resistance	Beta-lactam
Beta-lactam resistance	Beta-lactam
Beta-lactam resistance	Beta-lactam
Beta-lactam resistance	Beta-lactam
Beta-lactam resistance	Beta-lactam
Beta-lactam resistance	Beta-lactam
Beta-lactam resistance	Beta-lactam
Beta-lactam resistance	Beta-lactam
Beta-lactam resistance	Beta-lactam
Beta-lactam resistance	Beta-lactam
Beta-lactam resistance	Beta-lactam
Beta-lactam resistance	Beta-lactam
Beta-lactam resistance	Beta-lactam
Beta-lactam resistance	Beta-lactam
Beta-lactam resistance	Beta-lactam
Beta-lactam resistance	Beta-lactam
Beta-lactam resistance	Beta-lactam
Beta-lactam resistance	Beta-lactam
Beta-lactam resistance	Beta-lactam
Beta-lactam resistance	Beta-lactam
Beta-lactam resistance	Beta-lactam
Beta-lactam resistance	Beta-lactam
Beta-lactam resistance	Beta-lactam

Beta-lactam resistance	Beta-lactam
Beta-lactam resistance	Beta-lactam
Beta-lactam resistance	Beta-lactam
Beta-lactam resistance	Beta-lactam
Beta-lactam resistance	Beta-lactam
Beta-lactam resistance	Beta-lactam
Beta-lactam resistance	Beta-lactam
Beta-lactam resistance	Beta-lactam
Beta-lactam resistance	Beta-lactam
Beta-lactam resistance	Beta-lactam
Beta-lactam resistance	Beta-lactam
Beta-lactam resistance	Beta-lactam
Beta-lactam resistance	Beta-lactam
Beta-lactam resistance	Beta-lactam
Beta-lactam resistance	Beta-lactam
Beta-lactam resistance	Beta-lactam
Beta-lactam resistance	Beta-lactam
Beta-lactam resistance	Beta-lactam
Beta-lactam resistance	Beta-lactam
Beta-lactam resistance	Beta-lactam
Beta-lactam resistance	Beta-lactam
Beta-lactam resistance	Beta-lactam
Beta-lactam resistance	Beta-lactam
Beta-lactam resistance	Beta-lactam
Beta-lactam resistance	Beta-lactam
Beta-lactam resistance	Beta-lactam
Beta-lactam resistance	Beta-lactam
Beta-lactam resistance	Beta-lactam
Beta-lactam resistance	Beta-lactam
Beta-lactam resistance	Beta-lactam
Beta-lactam resistance	Beta-lactam
Beta-lactam resistance	Beta-lactam
Beta-lactam resistance	Beta-lactam
Beta-lactam resistance	Beta-lactam
Beta-lactam resistance	Beta-lactam
Beta-lactam resistance	Beta-lactam
Beta-lactam resistance	Beta-lactam
Beta-lactam resistance	Beta-lactam
Beta-lactam resistance	Beta-lactam
Beta-lactam resistance	Beta-lactam
Beta-lactam resistance	Beta-lactam
Beta-lactam resistance	Beta-lactam
Beta-lactam resistance	Beta-lactam
Beta-lactam resistance	Beta-lactam

Beta-lactam resistance	Beta-lactam
Beta-lactam resistance	Beta-lactam
Beta-lactam resistance	Beta-lactam
Beta-lactam resistance	Beta-lactam
Beta-lactam resistance	Beta-lactam
Beta-lactam resistance	Beta-lactam
Beta-lactam resistance	Beta-lactam
Beta-lactam resistance	Beta-lactam
Beta-lactam resistance	Beta-lactam
Beta-lactam resistance	Beta-lactam
Beta-lactam resistance	Beta-lactam
Beta-lactam resistance	Beta-lactam
Beta-lactam resistance	Beta-lactam
Beta-lactam resistance	Beta-lactam
Beta-lactam resistance	Beta-lactam
Beta-lactam resistance	Beta-lactam
Beta-lactam resistance	Beta-lactam
Beta-lactam resistance	Beta-lactam
Beta-lactam resistance	Beta-lactam
Beta-lactam resistance	Beta-lactam
Beta-lactam resistance	Beta-lactam
Beta-lactam resistance	Beta-lactam
Beta-lactam resistance	Beta-lactam
Beta-lactam resistance	Beta-lactam
Beta-lactam resistance	Beta-lactam
Beta-lactam resistance	Beta-lactam
Beta-lactam resistance	Beta-lactam
Beta-lactam resistance	Beta-lactam
Beta-lactam resistance	Beta-lactam
Beta-lactam resistance	Beta-lactam
Beta-lactam resistance	Beta-lactam
Beta-lactam resistance	Beta-lactam
Beta-lactam resistance	Beta-lactam
Beta-lactam resistance	Beta-lactam
Beta-lactam resistance	Beta-lactam
Beta-lactam resistance	Beta-lactam
Beta-lactam resistance	Beta-lactam
Beta-lactam resistance	Beta-lactam
Beta-lactam resistance	Beta-lactam
Beta-lactam resistance	Beta-lactam
Beta-lactam resistance	Beta-lactam
Beta-lactam resistance	Beta-lactam
Beta-lactam resistance	Beta-lactam
Beta-lactam resistance	Beta-lactam

Beta-lactam resistance	Beta-lactam
Beta-lactam resistance	Beta-lactam
Beta-lactam resistance	Beta-lactam
Beta-lactam resistance	Beta-lactam
Beta-lactam resistance	Beta-lactam
Beta-lactam resistance	Beta-lactam
Beta-lactam resistance	Beta-lactam
Beta-lactam resistance	Beta-lactam
Beta-lactam resistance	Beta-lactam
Beta-lactam resistance	Beta-lactam
Beta-lactam resistance	Beta-lactam
Beta-lactam resistance	Beta-lactam
Beta-lactam resistance	Beta-lactam
Beta-lactam resistance	Beta-lactam
Beta-lactam resistance	Beta-lactam
Beta-lactam resistance	Beta-lactam
Beta-lactam resistance	Beta-lactam
Beta-lactam resistance	Beta-lactam
Beta-lactam resistance	Beta-lactam
Beta-lactam resistance	Beta-lactam
Beta-lactam resistance	Beta-lactam
Beta-lactam resistance	Beta-lactam
Beta-lactam resistance	Beta-lactam
Beta-lactam resistance	Beta-lactam
Beta-lactam resistance	Beta-lactam
Beta-lactam resistance	Beta-lactam
Beta-lactam resistance	Beta-lactam
Beta-lactam resistance	Beta-lactam
Beta-lactam resistance	Beta-lactam
Beta-lactam resistance	Beta-lactam
Beta-lactam resistance	Beta-lactam
Beta-lactam resistance	Beta-lactam
Beta-lactam resistance	Beta-lactam
Beta-lactam resistance	Beta-lactam
Beta-lactam resistance	Beta-lactam
Beta-lactam resistance	Beta-lactam
Beta-lactam resistance	Beta-lactam
Beta-lactam resistance	Beta-lactam
Beta-lactam resistance	Beta-lactam
Beta-lactam resistance	Beta-lactam
Beta-lactam resistance	Beta-lactam
Beta-lactam resistance	Beta-lactam
Beta-lactam resistance	Beta-lactam
Beta-lactam resistance	Beta-lactam

Beta-lactam resistance	Beta-lactam
Beta-lactam resistance	Beta-lactam
Beta-lactam resistance	Beta-lactam
Beta-lactam resistance	Beta-lactam
Beta-lactam resistance	Beta-lactam
Beta-lactam resistance	Beta-lactam
Beta-lactam resistance	Beta-lactam
Beta-lactam resistance	Beta-lactam
Beta-lactam resistance	Beta-lactam
Beta-lactam resistance	Beta-lactam
Beta-lactam resistance	Beta-lactam
Beta-lactam resistance	Beta-lactam
Beta-lactam resistance	Beta-lactam
Beta-lactam resistance	Beta-lactam
Beta-lactam resistance	Beta-lactam
Beta-lactam resistance	Beta-lactam
Beta-lactam resistance	Beta-lactam
Beta-lactam resistance	Beta-lactam
Beta-lactam resistance	Beta-lactam
Beta-lactam resistance	Beta-lactam
Beta-lactam resistance	Beta-lactam
Beta-lactam resistance	Beta-lactam
Beta-lactam resistance	Beta-lactam
Beta-lactam resistance	Beta-lactam
Beta-lactam resistance	Beta-lactam
Beta-lactam resistance	Beta-lactam
Beta-lactam resistance	Beta-lactam
Beta-lactam resistance	Beta-lactam
Beta-lactam resistance	Beta-lactam
Beta-lactam resistance	Beta-lactam
Beta-lactam resistance	Beta-lactam
Beta-lactam resistance	Beta-lactam
Beta-lactam resistance	Beta-lactam
Beta-lactam resistance	Beta-lactam
Beta-lactam resistance	Beta-lactam
Beta-lactam resistance	Beta-lactam
Beta-lactam resistance	Beta-lactam
Beta-lactam resistance	Beta-lactam
Beta-lactam resistance	Beta-lactam
Beta-lactam resistance	Beta-lactam
Beta-lactam resistance	Beta-lactam
Beta-lactam resistance	Beta-lactam
Beta-lactam resistance	Beta-lactam
Beta-lactam resistance	Beta-lactam

Beta-lactam resistance	Beta-lactam
Beta-lactam resistance	Beta-lactam
Beta-lactam resistance	Beta-lactam
Beta-lactam resistance	Beta-lactam
Beta-lactam resistance	Beta-lactam
Beta-lactam resistance	Beta-lactam
Beta-lactam resistance	Beta-lactam
Beta-lactam resistance	Beta-lactam
Beta-lactam resistance	Beta-lactam
Beta-lactam resistance	Beta-lactam
Beta-lactam resistance	Beta-lactam
Beta-lactam resistance	Beta-lactam
Beta-lactam resistance	Beta-lactam
Beta-lactam resistance	Beta-lactam
Beta-lactam resistance	Beta-lactam
Beta-lactam resistance	Beta-lactam
Beta-lactam resistance	Beta-lactam
Beta-lactam resistance	Beta-lactam
Beta-lactam resistance	Beta-lactam
Beta-lactam resistance	Beta-lactam
Beta-lactam resistance	Beta-lactam
Beta-lactam resistance	Beta-lactam
Beta-lactam resistance	Beta-lactam
Beta-lactam resistance	Beta-lactam
Beta-lactam resistance	Beta-lactam
Beta-lactam resistance	Beta-lactam
Beta-lactam resistance	Beta-lactam
Beta-lactam resistance	Beta-lactam
Beta-lactam resistance	Beta-lactam
Beta-lactam resistance	Beta-lactam
Beta-lactam resistance	Beta-lactam
Beta-lactam resistance	Beta-lactam
Beta-lactam resistance	Beta-lactam
Beta-lactam resistance	Beta-lactam
Beta-lactam resistance	Beta-lactam
Beta-lactam resistance	Beta-lactam
Beta-lactam resistance	Beta-lactam
Beta-lactam resistance	Beta-lactam
Beta-lactam resistance	Beta-lactam
Beta-lactam resistance	Beta-lactam
Beta-lactam resistance	Beta-lactam
Beta-lactam resistance	Beta-lactam
Beta-lactam resistance	Beta-lactam
Beta-lactam resistance	Beta-lactam

Beta-lactam resistance	Beta-lactam
Beta-lactam resistance	Beta-lactam
Beta-lactam resistance	Beta-lactam
Beta-lactam resistance	Beta-lactam
Beta-lactam resistance	Beta-lactam
Beta-lactam resistance	Beta-lactam
Beta-lactam resistance	Beta-lactam
Beta-lactam resistance	Beta-lactam
Beta-lactam resistance	Beta-lactam
Beta-lactam resistance	Beta-lactam
Beta-lactam resistance	Beta-lactam
Beta-lactam resistance	Beta-lactam
Beta-lactam resistance	Beta-lactam
Beta-lactam resistance	Beta-lactam
Beta-lactam resistance	Beta-lactam
Beta-lactam resistance	Beta-lactam
Beta-lactam resistance	Beta-lactam
Beta-lactam resistance	Beta-lactam
Beta-lactam resistance	Beta-lactam
Beta-lactam resistance	Beta-lactam
Beta-lactam resistance	Beta-lactam
Beta-lactam resistance	Beta-lactam
Beta-lactam resistance	Beta-lactam
Beta-lactam resistance	Beta-lactam
Beta-lactam resistance	Beta-lactam
Beta-lactam resistance	Beta-lactam
Beta-lactam resistance	Beta-lactam
Beta-lactam resistance	Beta-lactam
Beta-lactam resistance	Beta-lactam
Beta-lactam resistance	Beta-lactam
Beta-lactam resistance	Beta-lactam
Beta-lactam resistance	Beta-lactam
Beta-lactam resistance	Beta-lactam
Beta-lactam resistance	Beta-lactam
Beta-lactam resistance	Beta-lactam
Beta-lactam resistance	Beta-lactam
Beta-lactam resistance	Beta-lactam
Beta-lactam resistance	Beta-lactam
Beta-lactam resistance	Beta-lactam
Beta-lactam resistance	Beta-lactam
Beta-lactam resistance	Beta-lactam
Beta-lactam resistance	Beta-lactam
Beta-lactam resistance	Beta-lactam
Beta-lactam resistance	Beta-lactam

Beta-lactam resistance	Beta-lactam
Beta-lactam resistance	Beta-lactam
Beta-lactam resistance	Beta-lactam
Beta-lactam resistance	Beta-lactam
Beta-lactam resistance	Beta-lactam
Beta-lactam resistance	Beta-lactam
Beta-lactam resistance	Beta-lactam
Beta-lactam resistance	Beta-lactam
Beta-lactam resistance	Beta-lactam
Beta-lactam resistance	Beta-lactam
Beta-lactam resistance	Beta-lactam
Beta-lactam resistance	Beta-lactam
Beta-lactam resistance	Beta-lactam
Beta-lactam resistance	Beta-lactam
Beta-lactam resistance	Beta-lactam
Beta-lactam resistance	Beta-lactam
Beta-lactam resistance	Beta-lactam
Beta-lactam resistance	Beta-lactam
Beta-lactam resistance	Beta-lactam
Beta-lactam resistance	Beta-lactam
Beta-lactam resistance	Beta-lactam
Beta-lactam resistance	Beta-lactam
Beta-lactam resistance	Beta-lactam
Beta-lactam resistance	Beta-lactam
Beta-lactam resistance	Beta-lactam
Beta-lactam resistance	Beta-lactam
Fluoroquinolone and aminoglycoside resistance	Aminoglycoside
Fluoroquinolone and aminoglycoside resistance	Aminoglycoside
Fluoroquinolone and aminoglycoside resistance	Aminoglycoside
Fluoroquinolone resistance	Quinolone
Fluoroquinolone resistance	Quinolone
Fluoroquinolone resistance	Quinolone
Fosfomycin resistance	Fosfomycin
Fosfomycin resistance	Fosfomycin
Fosfomycin resistance	Fosfomycin
Fosfomycin resistance	Fosfomycin
Fosfomycin resistance	Fosfomycin
Fosfomycin resistance	Fosfomycin
Fosfomycin resistance	Fosfomycin
Fosfomycin resistance	Fosfomycin
Fosfomycin resistance	Fosfomycin
Fosfomycin resistance	Fosfomycin
Fosfomycin resistance	Fosfomycin
Fosfomycin resistance	Fosfomycin

Fosfomycin resistance	Fosfomycin
Fosfomycin resistance	Fosfomycin
Fosfomycin resistance	Fosfomycin
Fosfomycin resistance	Fosfomycin
Fosfomycin resistance	Fosfomycin
Fosfomycin resistance	Fosfomycin
Fosfomycin resistance	Fosfomycin
Fosfomycin resistance	Fosfomycin
Fusidic acid resistance	Fusidic
Fusidic acid resistance	Fusidic
Fusidic acid resistance	Fusidic
Fusidic acid resistance	Fusidic
Lincosamide resistance	MLS
Lincosamide resistance	MLS
Lincosamide resistance	MLS
Lincosamide resistance	MLS
Lincosamide resistance	MLS
Lincosamide resistance	MLS
Lincosamide resistance	MLS
Lincosamide resistance	MLS
Lincosamide resistance	MLS
Macrolide resistance	MLS
Macrolide resistance	MLS
Macrolide resistance	MLS
Macrolide resistance	MLS
Macrolide resistance	MLS
Macrolide resistance	MLS
Macrolide resistance	MLS
Macrolide resistance	MLS
Macrolide resistance	MLS
Macrolide resistance	MLS
Macrolide resistance	MLS
Macrolide resistance	MLS
Macrolide resistance	MLS
Macrolide resistance	MLS
Macrolide resistance	MLS
Macrolide resistance	MLS
Macrolide resistance	MLS
Macrolide resistance	MLS
Macrolide resistance	MLS
Macrolide resistance	MLS
Macrolide resistance	MLS
Macrolide resistance	MLS
Macrolide resistance	MLS

Macrolide resistance	MLS
Macrolide resistance	MLS
Macrolide resistance	MLS
Macrolide resistance	MLS
Macrolide resistance	MLS
Macrolide resistance	MLS
Macrolide resistance	MLS
Macrolide resistance	MLS
Macrolide resistance	MLS
Macrolide resistance	MLS
Macrolide resistance	MLS
Macrolide resistance	MLS
Macrolide resistance	MLS
Macrolide resistance	MLS
Macrolide resistance	MLS
Macrolide resistance	MLS
Macrolide resistance	MLS
Macrolide resistance	MLS
Macrolide resistance	MLS
Macrolide resistance	MLS
Macrolide resistance	MLS
Macrolide resistance	MLS
Macrolide resistance	MLS
Macrolide resistance	MLS
Macrolide resistance	MLS
Macrolide resistance	MLS
Macrolide resistance	MLS
Macrolide resistance	MLS
Macrolide resistance	MLS
Macrolide resistance	MLS
Macrolide resistance	MLS
Macrolide resistance	MLS
Macrolide resistance	MLS
Macrolide resistance	MLS
Macrolide resistance	MLS
Macrolide resistance	MLS
Macrolide resistance	MLS
Macrolide resistance	MLS
Macrolide resistance	MLS
Macrolide resistance	MLS
Macrolide resistance	MLS
Macrolide resistance	MLS
Macrolide resistance	MLS
Macrolide resistance	MLS

Macrolide resistance	MLS
Macrolide resistance	MLS
Macrolide resistance	MLS
Macrolide resistance	MLS
Macrolide resistance	MLS
Macrolide resistance	MLS
Macrolide resistance	MLS
Macrolide resistance	MLS
Macrolide resistance	MLS
Macrolide resistance	MLS
Macrolide resistance	MLS
Macrolide resistance	MLS
Macrolide resistance	MLS
Macrolide resistance	MLS
Macrolide resistance	MLS
Macrolide resistance	MLS
Macrolide resistance	MLS
Macrolide resistance	MLS
Macrolide resistance	MLS
Macrolide resistance	MLS
Macrolide resistance	MLS
Macrolide resistance	MLS
Macrolide resistance	MLS
Macrolide resistance	MLS
Macrolide resistance	MLS
Macrolide, Lincosamide and Streptogramin B resistance	MLS
Macrolide, Lincosamide and Streptogramin B resistance	MLS
Macrolide, Lincosamide and Streptogramin B resistance	MLS
Macrolide, Lincosamide and Streptogramin B resistance	MLS
Macrolide, Lincosamide and Streptogramin B resistance	MLS
Macrolide, Lincosamide and Streptogramin B resistance	MLS
Macrolide, Lincosamide and Streptogramin B resistance	MLS
Macrolide, Lincosamide and Streptogramin B resistance	MLS
Macrolide, Lincosamide and Streptogramin B resistance	MLS
Macrolide, Lincosamide and Streptogramin B resistance	MLS
Macrolide, Lincosamide and Streptogramin B resistance	MLS
Macrolide, Lincosamide and Streptogramin B resistance	MLS
Macrolide, Lincosamide and Streptogramin B resistance	MLS
Macrolide, Lincosamide and Streptogramin B resistance	MLS
Macrolide, Lincosamide and Streptogramin B resistance	MLS
Phenicol resistance	Phenicol
Phenicol resistance	Phenicol
Phenicol resistance	Phenicol
Phenicol resistance	Phenicol

Phenicol resistance	Phenicol
Phenicol resistance	Phenicol
Phenicol resistance	Phenicol
Phenicol resistance	Phenicol
Phenicol resistance	Phenicol
Phenicol resistance	Phenicol
Phenicol resistance	Phenicol
Phenicol resistance	Phenicol
Phenicol resistance	Phenicol
Phenicol resistance	Phenicol
Phenicol resistance	Phenicol
Phenicol resistance	Phenicol
Phenicol resistance	Phenicol
Phenicol resistance	Phenicol
Phenicol resistance	Phenicol
Phenicol resistance	Phenicol
Phenicol resistance	Phenicol
Phenicol resistance	Phenicol
Phenicol resistance	Phenicol
Phenicol resistance	Phenicol
Phenicol resistance	Phenicol
Phenicol resistance	Phenicol
Phenicol resistance	Phenicol
Phenicol resistance	Phenicol
Phenicol resistance	Phenicol
Phenicol resistance	Phenicol
Phenicol resistance	Phenicol
Phenicol resistance	Phenicol
Phenicol resistance	Phenicol
Phenicols, Lincosamides, Oxazolidinones, Pleuromutilins, an	Phenicol
Phenicols, Lincosamides, Oxazolidinones, Pleuromutilins, an	Phenicol
Quinolone resistance	Quinolone
Quinolone resistance	Quinolone
Quinolone resistance	Quinolone
Quinolone resistance	Quinolone
Quinolone resistance	Quinolone
Quinolone resistance	Quinolone
Quinolone resistance	Quinolone
Quinolone resistance	Quinolone
Quinolone resistance	Quinolone
Quinolone resistance	Quinolone
Quinolone resistance	Quinolone
Quinolone resistance	Quinolone
Quinolone resistance	Quinolone

Quinolone resistance	Quinolone
Quinolone resistance	Quinolone
Quinolone resistance	Quinolone
Quinolone resistance	Quinolone
Quinolone resistance	Quinolone
Quinolone resistance	Quinolone
Quinolone resistance	Quinolone
Quinolone resistance	Quinolone
Quinolone resistance	Quinolone
Quinolone resistance	Quinolone
Quinolone resistance	Quinolone
Quinolone resistance	Quinolone
Quinolone resistance	Quinolone
Quinolone resistance	Quinolone
Quinolone resistance	Quinolone
Quinolone resistance	Quinolone
Quinolone resistance	Quinolone
Quinolone resistance	Quinolone
Quinolone resistance	Quinolone
Quinolone resistance	Quinolone
Quinolone resistance	Quinolone
Quinolone resistance	Quinolone
Quinolone resistance	Quinolone
Quinolone resistance	Quinolone
Quinolone resistance	Quinolone
Quinolone resistance	Quinolone
Quinolone resistance	Quinolone
Quinolone resistance	Quinolone
Quinolone resistance	Quinolone
Quinolone resistance	Quinolone
Quinolone resistance	Quinolone
Quinolone resistance	Quinolone
Quinolone resistance	Quinolone
Quinolone resistance	Quinolone
Quinolone resistance	Quinolone
Quinolone resistance	Quinolone
Quinolone resistance	Quinolone
Quinolone resistance	Quinolone
Quinolone resistance	Quinolone
Quinolone resistance	Quinolone
Quinolone resistance	Quinolone
Quinolone resistance	Quinolone
Quinolone resistance	Quinolone
Quinolone resistance	Quinolone

Quinolone resistance	Quinolone
Quinolone resistance	Quinolone
Quinolone resistance	Quinolone
Quinolone resistance	Quinolone
Quinolone resistance	Quinolone
Quinolone resistance	Quinolone
Quinolone resistance	Quinolone
Quinolone resistance	Quinolone
Quinolone resistance	Quinolone
Quinolone resistance	Quinolone
Quinolone resistance	Quinolone
Quinolone resistance	Quinolone
Quinolone resistance	Quinolone
Quinolone resistance	Quinolone
Quinolone resistance	Quinolone
Quinolone resistance	Quinolone
Quinolone resistance	Quinolone
Quinolone resistance	Quinolone
Quinolone resistance	Quinolone
Quinolone resistance	Quinolone
Quinolone resistance	Quinolone
Quinolone resistance	Quinolone
Quinolone resistance	Quinolone
Quinolone resistance	Quinolone
Quinolone resistance	Quinolone
Quinolone resistance	Quinolone
Quinolone resistance	Quinolone
Quinolone resistance	Quinolone
Quinolone resistance	Quinolone
Quinolone resistance	Quinolone
Quinolone resistance	Quinolone
Quinolone resistance	Quinolone
Quinolone resistance	Quinolone
Quinolone resistance	Quinolone
Quinolone resistance	Quinolone
Quinolone resistance	Quinolone
Quinolone resistance	Quinolone
Quinolone resistance	Quinolone
Quinolone resistance	Quinolone
Quinolone resistance	Quinolone
Quinolone resistance	Quinolone
Quinolone resistance	Quinolone
Quinolone resistance	Quinolone
Quinolone resistance	Quinolone

Quinolone resistance	Quinolone
Quinolone resistance	Quinolone
Quinolone resistance	Quinolone
Quinolone resistance	Quinolone
Quinolone resistance	Quinolone
Quinolone resistance	Quinolone
Quinolone resistance	Quinolone
Rifampicin resistance	Rifampicin
Rifampicin resistance	Rifampicin
Rifampicin resistance	Rifampicin
Rifampicin resistance	Rifampicin
Rifampicin resistance	Rifampicin
Rifampicin resistance	Rifampicin
Rifampicin resistance	Rifampicin
Rifampicin resistance	Rifampicin
Rifampicin resistance	Rifampicin
Rifampicin resistance	Rifampicin
Rifampicin resistance	Rifampicin
Rifampicin resistance	Rifampicin
Streptogramin B resistance	MLS
Streptogramin B resistance	MLS
Streptogramin B resistance	MLS
Streptogramin B resistance	MLS
Streptogramin B resistance	MLS
Streptogramin B resistance	MLS
Streptogramin B resistance	MLS
Streptogramin B resistance	MLS
Streptogramin B resistance	MLS
Streptogramin B resistance	MLS
Streptogramin B resistance	MLS
Streptogramin B resistance	MLS
Streptogramin B resistance	MLS
Streptogramin B resistance	MLS
Streptogramin B resistance	MLS
Streptogramin B resistance	MLS
Streptogramin B resistance	MLS
Streptogramin B resistance	MLS
Streptogramin B resistance	MLS
Streptogramin B resistance	MLS
Streptogramin B resistance	MLS
Streptogramin B resistance	MLS
Streptogramin B resistance	MLS
Sulphonamide resistance	Sulphonamide
Sulphonamide resistance	Sulphonamide

Sulphonamide resistance	Sulphonamide
Sulphonamide resistance	Sulphonamide
Sulphonamide resistance	Sulphonamide
Sulphonamide resistance	Sulphonamide
Sulphonamide resistance	Sulphonamide
Sulphonamide resistance	Sulphonamide
Sulphonamide resistance	Sulphonamide
Sulphonamide resistance	Sulphonamide
Sulphonamide resistance	Sulphonamide
Sulphonamide resistance	Sulphonamide
Sulphonamide resistance	Sulphonamide
Sulphonamide resistance	Sulphonamide
Sulphonamide resistance	Sulphonamide
Sulphonamide resistance	Sulphonamide
Sulphonamide resistance	Sulphonamide
Sulphonamide resistance	Sulphonamide
Sulphonamide resistance	Sulphonamide
Sulphonamide resistance	Sulphonamide
Sulphonamide resistance	Sulphonamide
Sulphonamide resistance	Sulphonamide
Sulphonamide resistance	Sulphonamide
Sulphonamide resistance	Sulphonamide
Sulphonamide resistance	Sulphonamide
Sulphonamide resistance	Sulphonamide
Sulphonamide resistance	Sulphonamide
Sulphonamide resistance	Sulphonamide
Sulphonamide resistance	Sulphonamide
Sulphonamide resistance	Sulphonamide
Sulphonamide resistance	Sulphonamide
Sulphonamide resistance	Sulphonamide
Sulphonamide resistance	Sulphonamide
Sulphonamide resistance	Sulphonamide
Sulphonamide resistance	Sulphonamide
Sulphonamide resistance	Sulphonamide
Sulphonamide resistance	Sulphonamide
Sulphonamide resistance	Sulphonamide
Sulphonamide resistance	Sulphonamide
Sulphonamide resistance	Sulphonamide
Sulphonamide resistance	Sulphonamide
Sulphonamide resistance	Sulphonamide
Sulphonamide resistance	Sulphonamide
Sulphonamide resistance	Sulphonamide
Sulphonamide resistance	Sulphonamide
Sulphonamide resistance	Sulphonamide

Sulphonamide resistance	Sulphonamide
Sulphonamide resistance	Sulphonamide
Sulphonamide resistance	Sulphonamide
Sulphonamide resistance	Sulphonamide
Sulphonamide resistance	Sulphonamide
Sulphonamide resistance	Sulphonamide
Sulphonamide resistance	Sulphonamide
Sulphonamide resistance	Sulphonamide
Sulphonamide resistance	Sulphonamide
Tetracycline resistance	Tetracycline
Tetracycline resistance	Tetracycline
Tetracycline resistance	Tetracycline
Tetracycline resistance	Tetracycline
Tetracycline resistance	Tetracycline
Tetracycline resistance	Tetracycline
Tetracycline resistance	Tetracycline
Tetracycline resistance	Tetracycline
Tetracycline resistance	Tetracycline
Tetracycline resistance	Tetracycline
Tetracycline resistance	Tetracycline
Tetracycline resistance	Tetracycline
Tetracycline resistance	Tetracycline
Tetracycline resistance	Tetracycline
Tetracycline resistance	Tetracycline
Tetracycline resistance	Tetracycline
Tetracycline resistance	Tetracycline
Tetracycline resistance	Tetracycline
Tetracycline resistance	Tetracycline
Tetracycline resistance	Tetracycline
Tetracycline resistance	Tetracycline
Tetracycline resistance	Tetracycline
Tetracycline resistance	Tetracycline
Tetracycline resistance	Tetracycline
Tetracycline resistance	Tetracycline
Tetracycline resistance	Tetracycline
Tetracycline resistance	Tetracycline
Tetracycline resistance	Tetracycline
Tetracycline resistance	Tetracycline
Tetracycline resistance	Tetracycline
Tetracycline resistance	Tetracycline
Tetracycline resistance	Tetracycline
Tetracycline resistance	Tetracycline
Tetracycline resistance	Tetracycline
Tetracycline resistance	Tetracycline

Tetracycline resistance	Tetracycline
Tetracycline resistance	Tetracycline
Tetracycline resistance	Tetracycline
Tetracycline resistance	Tetracycline
Tetracycline resistance	Tetracycline
Tetracycline resistance	Tetracycline
Tetracycline resistance	Tetracycline
Tetracycline resistance	Tetracycline
Tetracycline resistance	Tetracycline
Tetracycline resistance	Tetracycline
Tetracycline resistance	Tetracycline
Tetracycline resistance	Tetracycline
Tetracycline resistance	Tetracycline
Tetracycline resistance	Tetracycline
Tetracycline resistance	Tetracycline
Tetracycline resistance	Tetracycline
Tetracycline resistance	Tetracycline
Tetracycline resistance	Tetracycline
Tetracycline resistance	Tetracycline
Tetracycline resistance	Tetracycline
Tetracycline resistance	Tetracycline
Tetracycline resistance	Tetracycline
Tetracycline resistance	Tetracycline
Tetracycline resistance	Tetracycline
Tetracycline resistance	Tetracycline
Tetracycline resistance	Tetracycline
Tetracycline resistance	Tetracycline
Tetracycline resistance	Tetracycline
Tetracycline resistance	Tetracycline
Tetracycline resistance	Tetracycline
Tetracycline resistance	Tetracycline
Tetracycline resistance	Tetracycline
Tetracycline resistance	Tetracycline
Tetracycline resistance	Tetracycline
Tetracycline resistance	Tetracycline
Tetracycline resistance	Tetracycline
Tetracycline resistance	Tetracycline
Tetracycline resistance	Tetracycline
Tetracycline resistance	Tetracycline
Tetracycline resistance	Tetracycline
Tetracycline resistance	Tetracycline
Tetracycline resistance	Tetracycline
Tetracycline resistance	Tetracycline
Tetracycline resistance	Tetracycline

Tetracycline resistance	Tetracycline
Tetracycline resistance	Tetracycline
Tetracycline resistance	Tetracycline
Tetracycline resistance	Tetracycline
Tetracycline resistance	Tetracycline
Tetracycline resistance	Tetracycline
Tetracycline resistance	Tetracycline
Tetracycline resistance	Tetracycline
Tetracycline resistance	Tetracycline
Tetracycline resistance	Tetracycline
Tetracycline resistance	Tetracycline
Tetracycline resistance	Tetracycline
Tetracycline resistance	Tetracycline
Tetracycline resistance	Tetracycline
Tetracycline resistance	Tetracycline
Tetracycline resistance	Tetracycline
Tetracycline resistance	Tetracycline
Tetracycline resistance	Tetracycline
Tetracycline resistance	Tetracycline
Tetracycline resistance	Tetracycline
Tetracycline resistance	Tetracycline
Tetracycline resistance	Tetracycline
Tetracycline resistance	Tetracycline
Tetracycline resistance	Tetracycline
Tetracycline resistance	Tetracycline
Tetracycline resistance	Tetracycline
Tetracycline resistance	Tetracycline
Tetracycline resistance	Tetracycline
Tetracycline resistance	Tetracycline
Tetracycline resistance	Tetracycline
Tetracycline resistance	Tetracycline
Tetracycline resistance	Tetracycline
Tetracycline resistance	Tetracycline
Tetracycline resistance	Tetracycline
Tetracycline resistance	Tetracycline
Tetracycline resistance	Tetracycline
Tetracycline resistance	Tetracycline
Trimethoprim resistance	Trimethoprim
Trimethoprim resistance	Trimethoprim
Trimethoprim resistance	Trimethoprim
Trimethoprim resistance	Trimethoprim
Trimethoprim resistance	Trimethoprim
Trimethoprim resistance	Trimethoprim
Trimethoprim resistance	Trimethoprim

Trimethoprim resistance	Trimethoprim
Trimethoprim resistance	Trimethoprim
Trimethoprim resistance	Trimethoprim
Trimethoprim resistance	Trimethoprim
Trimethoprim resistance	Trimethoprim
Trimethoprim resistance	Trimethoprim
Trimethoprim resistance	Trimethoprim
Trimethoprim resistance	Trimethoprim
Trimethoprim resistance	Trimethoprim
Trimethoprim resistance	Trimethoprim
Trimethoprim resistance	Trimethoprim
Trimethoprim resistance	Trimethoprim
Trimethoprim resistance	Trimethoprim
Trimethoprim resistance	Trimethoprim
Trimethoprim resistance	Trimethoprim
Trimethoprim resistance	Trimethoprim
Trimethoprim resistance	Trimethoprim
Trimethoprim resistance	Trimethoprim
Trimethoprim resistance	Trimethoprim
Trimethoprim resistance	Trimethoprim
Trimethoprim resistance	Trimethoprim
Trimethoprim resistance	Trimethoprim
Trimethoprim resistance	Trimethoprim
Trimethoprim resistance	Trimethoprim
Trimethoprim resistance	Trimethoprim
Trimethoprim resistance	Trimethoprim
Trimethoprim resistance	Trimethoprim
Trimethoprim resistance	Trimethoprim
Trimethoprim resistance	Trimethoprim
Trimethoprim resistance	Trimethoprim
Trimethoprim resistance	Trimethoprim
Trimethoprim resistance	Trimethoprim
Trimethoprim resistance	Trimethoprim
Trimethoprim resistance	Trimethoprim
Trimethoprim resistance	Trimethoprim
Trimethoprim resistance	Trimethoprim
Trimethoprim resistance	Trimethoprim
Trimethoprim resistance	Trimethoprim
Trimethoprim resistance	Trimethoprim
Trimethoprim resistance	Trimethoprim
Trimethoprim resistance	Trimethoprim
Trimethoprim resistance	Trimethoprim
Trimethoprim resistance	Trimethoprim
Trimethoprim resistance	Trimethoprim

Trimethoprim resistance	Trimethoprim
Trimethoprim resistance	Trimethoprim
Trimethoprim resistance	Trimethoprim
Trimethoprim resistance	Trimethoprim
Trimethoprim resistance	Trimethoprim
Trimethoprim resistance	Trimethoprim
Trimethoprim resistance	Trimethoprim
Trimethoprim resistance	Trimethoprim
VanA vancomycin resistance operon, (VanA-A, VanH-A, VanI	Glycopeptide
VanA vancomycin resistance operon, (VanA-A, VanH-A, VanI	Glycopeptide
VanA vancomycin resistance operon, (VanA-A, VanH-A, VanI	Glycopeptide
VanA vancomycin resistance operon, (VanA-A, VanH-A, VanI	Glycopeptide
VanA vancomycin resistance operon, (VanA-A, VanH-A, VanI	Glycopeptide
VanA vancomycin resistance operon, (VanA-A, VanH-A, VanI	Glycopeptide
VanA vancomycin resistance operon, (VanA-A, VanH-A, VanI	Glycopeptide
VanA vancomycin resistance operon, (VanA-A, VanH-A, VanI	Glycopeptide
VanA vancomycin resistance operon, (VanA-A, VanH-A, VanI	Glycopeptide
VanA vancomycin resistance operon, (VanA-A, VanH-A, VanI	Glycopeptide
VanA vancomycin resistance operon, (VanA-A, VanH-A, VanI	Glycopeptide
VanA vancomycin resistance operon, (VanA-A, VanH-A, VanI	Glycopeptide
VanAc1 vancomycin resistance operon, (VanH-Ac1 and VanX	Glycopeptide
VanAc1 vancomycin resistance operon, (VanH-Ac1 and VanX	Glycopeptide
VanAc2 vancomycin resistance operon, (VanH-Ac2 and VanX	Glycopeptide
VanAc2 vancomycin resistance operon, (VanH-Ac2 and VanX	Glycopeptide
VanAo1 vancomycin resistance operon, (VanH-Ao1 and Van)	Glycopeptide
VanAo1 vancomycin resistance operon, (VanH-Ao1 and Van)	Glycopeptide
VanAo2 vancomycin resistance operon, (VanA-Ao2, VanH-A(	Glycopeptide
VanAo2 vancomycin resistance operon, (VanA-Ao2, VanH-A(	Glycopeptide
VanAo2 vancomycin resistance operon, (VanA-Ao2, VanH-A(	Glycopeptide
VanAo2 vancomycin resistance operon, (VanA-Ao2, VanH-A(	Glycopeptide
VanB vancomycin resistance operon, (VanA-B, VanH-B, VanF	Glycopeptide
VanB vancomycin resistance operon, (VanA-B, VanH-B, VanF	Glycopeptide
VanB vancomycin resistance operon, (VanA-B, VanH-B, VanF	Glycopeptide
VanB vancomycin resistance operon, (VanA-B, VanH-B, VanF	Glycopeptide
VanB vancomycin resistance operon, (VanA-B, VanH-B, VanF	Glycopeptide
VanB vancomycin resistance operon, (VanA-B, VanH-B, VanF	Glycopeptide
VanB vancomycin resistance operon, (VanA-B, VanH-B, VanF	Glycopeptide
VanC vancomycin resistance operon, (VanA-C, VanR-C, VanS	Glycopeptide
VanC vancomycin resistance operon, (VanA-C, VanR-C, VanS	Glycopeptide
VanC vancomycin resistance operon, (VanA-C, VanR-C, VanS	Glycopeptide
VanC vancomycin resistance operon, (VanA-C, VanR-C, VanS	Glycopeptide
VanC vancomycin resistance operon, (VanA-C, VanR-C, VanS	Glycopeptide
VanC vancomycin resistance operon, (VanA-C, VanR-C, VanS	Glycopeptide
VanC vancomycin resistance operon, (VanA-C, VanR-C, VanS	Glycopeptide

VanC vancomycin resistance operon, (VanA-C, VanR-C, VanS	Glycopeptide
VanC vancomycin resistance operon, (VanA-C, VanR-C, VanS	Glycopeptide
VanC vancomycin resistance operon, (VanA-C, VanR-C, VanS	Glycopeptide
VanC vancomycin resistance operon, (VanA-C, VanR-C, VanS	Glycopeptide
VanC vancomycin resistance operon, (VanA-C, VanR-C, VanS	Glycopeptide
VanC vancomycin resistance operon, (VanA-C, VanR-C, VanS	Glycopeptide
VanC vancomycin resistance operon, (VanA-C, VanR-C, VanS	Glycopeptide
VanC vancomycin resistance operon, (VanA-C, VanR-C, VanS	Glycopeptide
VanC vancomycin resistance operon, (VanA-C, VanR-C, VanS	Glycopeptide
VanC vancomycin resistance operon, (VanA-C, VanR-C, VanS	Glycopeptide
VanC vancomycin resistance operon, (VanA-C, VanR-C, VanS	Glycopeptide
VanC vancomycin resistance operon, (VanA-C, VanR-C, VanS	Glycopeptide
VanC vancomycin resistance operon, (VanA-C, VanR-C, VanS	Glycopeptide
VanC vancomycin resistance operon, (VanA-C, VanR-C, VanS	Glycopeptide
VanC vancomycin resistance operon, (VanA-C, VanR-C, VanS	Glycopeptide
VanD vancomycin resistance operon, (VanA-D, VanH-D, VanI	Glycopeptide
VanD vancomycin resistance operon, (VanA-D, VanH-D, VanI	Glycopeptide
VanD vancomycin resistance operon, (VanA-D, VanH-D, VanI	Glycopeptide
VanD vancomycin resistance operon, (VanA-D, VanH-D, VanI	Glycopeptide
VanD vancomycin resistance operon, (VanA-D, VanH-D, VanI	Glycopeptide
VanD vancomycin resistance operon, (VanA-D, VanH-D, VanI	Glycopeptide
VanD vancomycin resistance operon, (VanA-D, VanH-D, VanI	Glycopeptide
VanD vancomycin resistance operon, (VanA-D, VanH-D, VanI	Glycopeptide
VanD vancomycin resistance operon, (VanA-D, VanH-D, VanI	Glycopeptide
VanD vancomycin resistance operon, (VanA-D, VanH-D, VanI	Glycopeptide
VanD vancomycin resistance operon, (VanA-D, VanH-D, VanI	Glycopeptide
VanD vancomycin resistance operon, (VanA-D, VanH-D, VanI	Glycopeptide
VanD vancomycin resistance operon, (VanA-D, VanH-D, VanI	Glycopeptide
VanD vancomycin resistance operon, (VanA-D, VanH-D, VanI	Glycopeptide
VanD vancomycin resistance operon, (VanA-D, VanH-D, VanI	Glycopeptide
VanD vancomycin resistance operon, (VanA-D, VanH-D, VanI	Glycopeptide
VanD vancomycin resistance operon, (VanA-D, VanH-D, VanI	Glycopeptide
VanD vancomycin resistance operon, (VanA-D, VanH-D, VanI	Glycopeptide
VanD vancomycin resistance operon, (VanA-D, VanH-D, VanI	Glycopeptide
VanD vancomycin resistance operon, (VanA-D, VanH-D, VanI	Glycopeptide
VanD vancomycin resistance operon, (VanA-D, VanH-D, VanI	Glycopeptide
VanD vancomycin resistance operon, (VanA-D, VanH-D, VanI	Glycopeptide
VanD vancomycin resistance operon, (VanA-D, VanH-D, VanI	Glycopeptide
VanE vancomycin resistance operon, (VanA-E, VanH-E and V	Glycopeptide
VanE vancomycin resistance operon, (VanA-E, VanH-E and V	Glycopeptide
VanE vancomycin resistance operon, (VanA-E, VanH-E and V	Glycopeptide
VanF vancomycin resistance operon, (VanR-F, VanS-F, VanY-	Glycopeptide
VanF vancomycin resistance operon, (VanR-F, VanS-F, VanY-	Glycopeptide
VanF vancomycin resistance operon, (VanR-F, VanS-F, VanY-	Glycopeptide

VanF vancomycin resistance operon, (VanR-F, VanS-F, VanY-	Glycopeptide
VanG vancomycin resistance operon, (VanA-G, VanR-G, VanS	Glycopeptide
VanG vancomycin resistance operon, (VanA-G, VanR-G, VanS	Glycopeptide
VanG vancomycin resistance operon, (VanA-G, VanR-G, VanS	Glycopeptide
VanG vancomycin resistance operon, (VanA-G, VanR-G, VanS	Glycopeptide
VanG vancomycin resistance operon, (VanA-G, VanR-G, VanS	Glycopeptide
VanG vancomycin resistance operon, (VanA-G, VanR-G, VanS	Glycopeptide
VanG vancomycin resistance operon, (VanA-G, VanR-G, VanS	Glycopeptide
VanG vancomycin resistance operon, (VanA-G, VanR-G, VanS	Glycopeptide
VanL vancomycin resistance operon, (VanA-L, VanR-L, VanS-	Glycopeptide
VanL vancomycin resistance operon, (VanA-L, VanR-L, VanS-	Glycopeptide
VanL vancomycin resistance operon, (VanA-L, VanR-L, VanS-	Glycopeptide
VanL vancomycin resistance operon, (VanA-L, VanR-L, VanS-	Glycopeptide
VanL vancomycin resistance operon, (VanA-L, VanR-L, VanS-	Glycopeptide
VanL vancomycin resistance operon, (VanA-L, VanR-L, VanS-	Glycopeptide
VanM vancomycin resistance operon, (VanA-M, VanH-M, Va	Glycopeptide
VanM vancomycin resistance operon, (VanA-M, VanH-M, Va	Glycopeptide
VanM vancomycin resistance operon, (VanA-M, VanH-M, Va	Glycopeptide
VanM vancomycin resistance operon, (VanA-M, VanH-M, Va	Glycopeptide
VanM vancomycin resistance operon, (VanA-M, VanH-M, Va	Glycopeptide
VanM vancomycin resistance operon, (VanA-M, VanH-M, Va	Glycopeptide
VanN vancomycin resistance operon, (VanA-N, VanR-N, VanS	Glycopeptide
VanN vancomycin resistance operon, (VanA-N, VanR-N, VanS	Glycopeptide
VanN vancomycin resistance operon, (VanA-N, VanR-N, VanS	Glycopeptide
VanN vancomycin resistance operon, (VanA-N, VanR-N, VanS	Glycopeptide
VanN vancomycin resistance operon, (VanA-N, VanR-N, VanS	Glycopeptide
VanPp vancomycin resistance operon, (VanAE-Pp, VanHE-Pp	Glycopeptide
VanPp vancomycin resistance operon, (VanAE-Pp, VanHE-Pp	Glycopeptide
VanPp vancomycin resistance operon, (VanAE-Pp, VanHE-Pp	Glycopeptide
VanPp vancomycin resistance operon, (VanAE-Pp, VanHE-Pp	Glycopeptide
VanPp vancomycin resistance operon, (VanAE-Pp, VanHE-Pp	Glycopeptide
VanPt vancomycin resistance operon, (VanA-Pt, VanH-Pt, Va	Glycopeptide
VanPt vancomycin resistance operon, (VanA-Pt, VanH-Pt, Va	Glycopeptide
VanPt vancomycin resistance operon, (VanA-Pt, VanH-Pt, Va	Glycopeptide
VanPt vancomycin resistance operon, (VanA-Pt, VanH-Pt, Va	Glycopeptide
VanPt vancomycin resistance operon, (VanA-Pt, VanH-Pt, Va	Glycopeptide
VanPt vancomycin resistance operon, (VanA-Pt, VanH-Pt, Va	Glycopeptide
VanPt vancomycin resistance operon, (VanA-Pt, VanH-Pt, Va	Glycopeptide
VanPt vancomycin resistance operon, (VanA-Pt, VanH-Pt, Va	Glycopeptide
VanPt vancomycin resistance operon, (VanA-Pt, VanH-Pt, Va	Glycopeptide
VanPt vancomycin resistance operon, (VanA-Pt, VanH-Pt, Va	Glycopeptide
VanPt vancomycin resistance operon, (VanA-Pt, VanH-Pt, Va	Glycopeptide
VanPt vancomycin resistance operon, (VanA-Pt, VanH-Pt, Va	Glycopeptide
VanPt vancomycin resistance operon, (VanA-Pt, VanH-Pt, Va	Glycopeptide

VanPt vancomycin resistance operon, (VanA-Pt, VanH-Pt, Va	Glycopeptide
VanPt vancomycin resistance operon, (VanA-Pt, VanH-Pt, Va	Glycopeptide
VanPt2 vancomycin resistance operon, (VanA-Pt2, VanH-Pt2	Glycopeptide
VanPt2 vancomycin resistance operon, (VanA-Pt2, VanH-Pt2	Glycopeptide
VanPt2 vancomycin resistance operon, (VanA-Pt2, VanH-Pt2	Glycopeptide
VanPt2 vancomycin resistance operon, (VanA-Pt2, VanH-Pt2	Glycopeptide
VanPt2 vancomycin resistance operon, (VanA-Pt2, VanH-Pt2	Glycopeptide
VanPt2 vancomycin resistance operon, (VanA-Pt2, VanH-Pt2	Glycopeptide
VanSc vancomycin resistance operon, (VanA-Sc, VanH-Sc an	Glycopeptide
VanSc vancomycin resistance operon, (VanA-Sc, VanH-Sc an	Glycopeptide
VanSc vancomycin resistance operon, (VanA-Sc, VanH-Sc an	Glycopeptide
metronidazole (5-nitroimidazole)	Metronidazole
metronidazole (5-nitroimidazole)	Metronidazole
metronidazole (5-nitroimidazole)	Metronidazole
metronidazole (5-nitroimidazole)	Metronidazole
metronidazole (5-nitroimidazole)	Metronidazole
metronidazole (5-nitroimidazole)	Metronidazole
metronidazole (5-nitroimidazole)	Metronidazole
metronidazole (5-nitroimidazole)	Metronidazole
metronidazole (5-nitroimidazole)	Metronidazole
metronidazole (5-nitroimidazole)	Metronidazole
metronidazole (5-nitroimidazole)	Metronidazole
metronidazole (5-nitroimidazole)	Metronidazole
metronidazole (5-nitroimidazole)	Metronidazole
metronidazole (5-nitroimidazole)	Metronidazole

Supplementary Figures 1-4

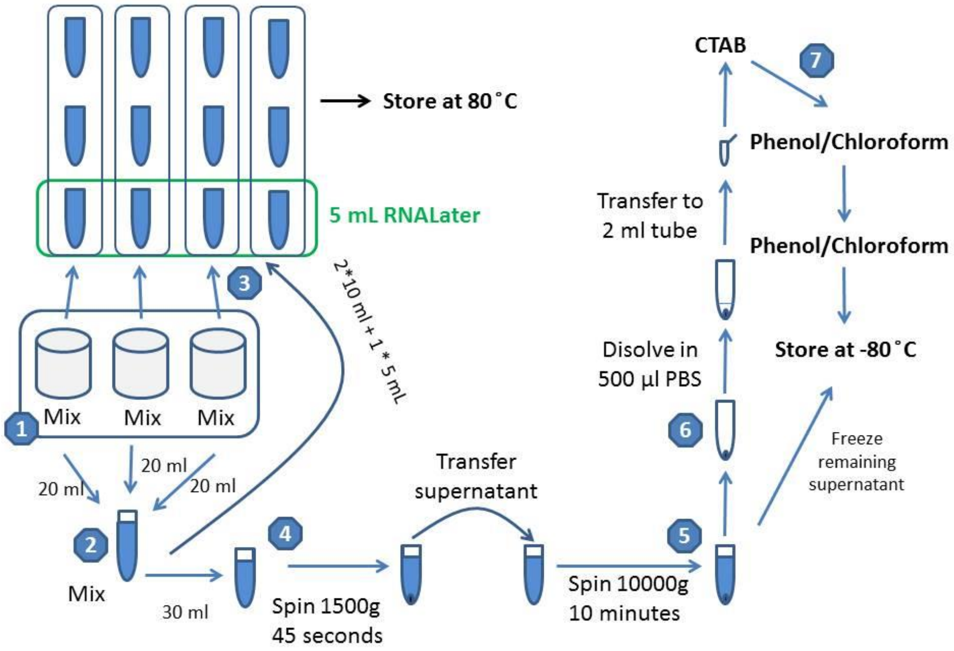

Supplementary Figure 1 Workflow for handling the air toilet samples.

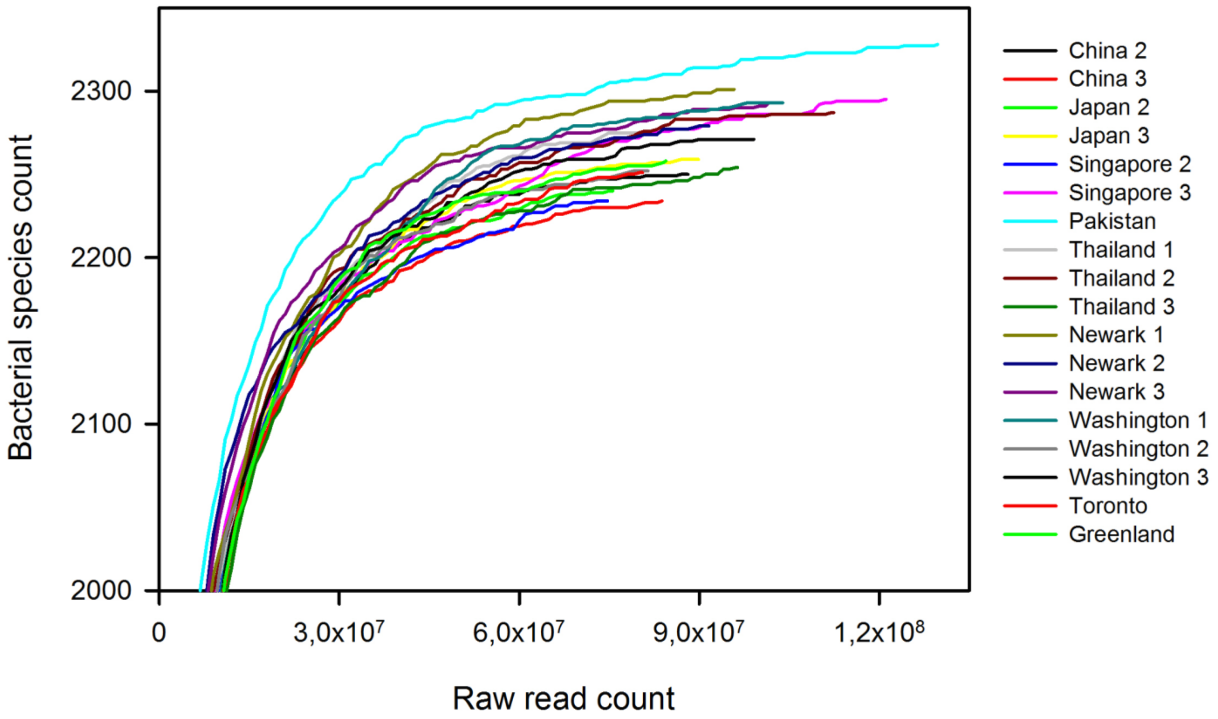

Supplementary Figure 2. Rarefaction curves of bacterial species found in the metagenomics samples. The rarefaction was done as number of new bacteria found as a result of the chain mapping procedure per raw reads mapped. Sampling was done for every 1 million reads.

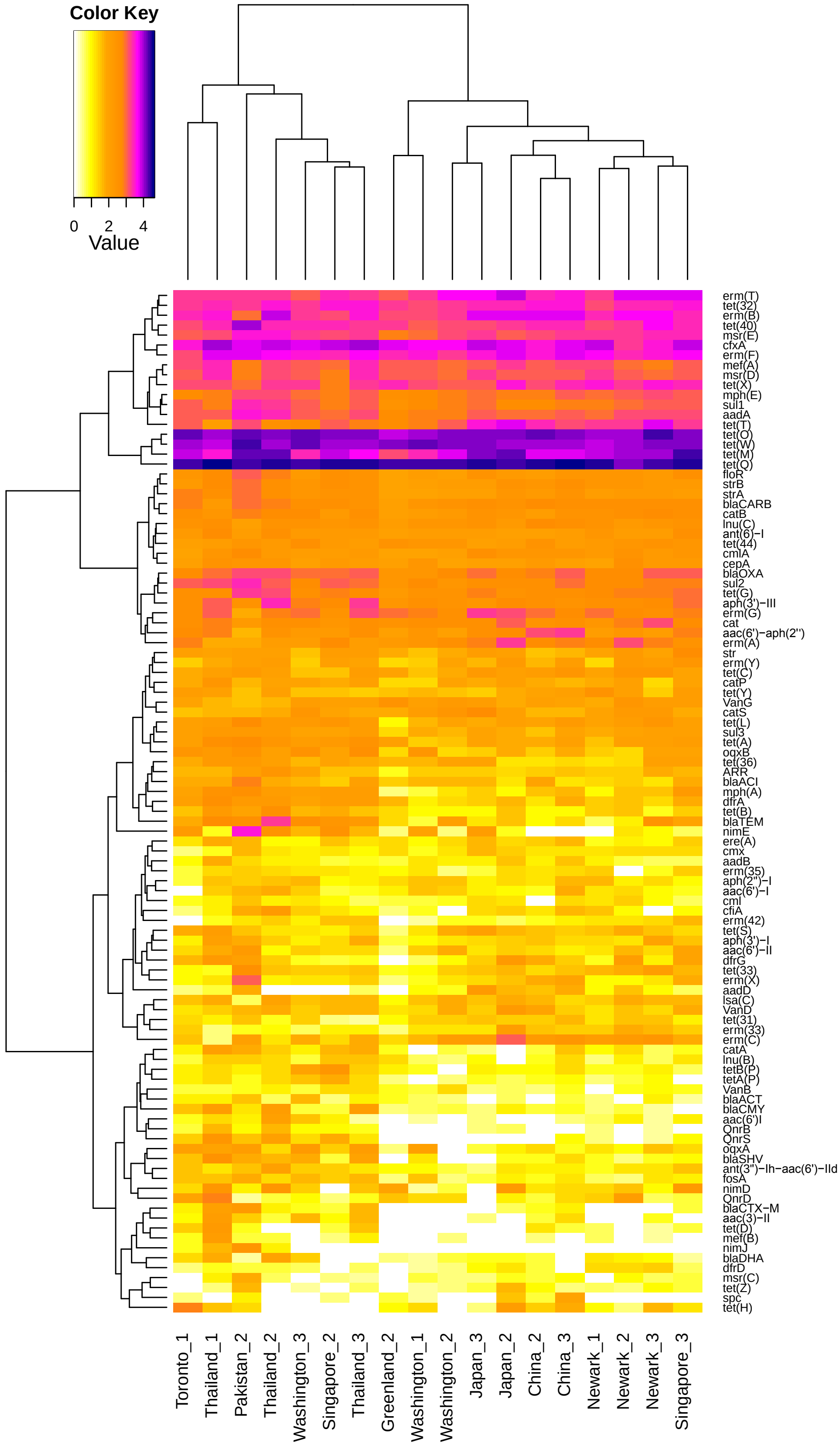

Supplemental Figure 3. Clustering of the 50% most abundant resistance genes and sample resistance gene profiles. The abundance is in log10 scale from white (low), yellow, orange (intermediate), magenta, blue (high).

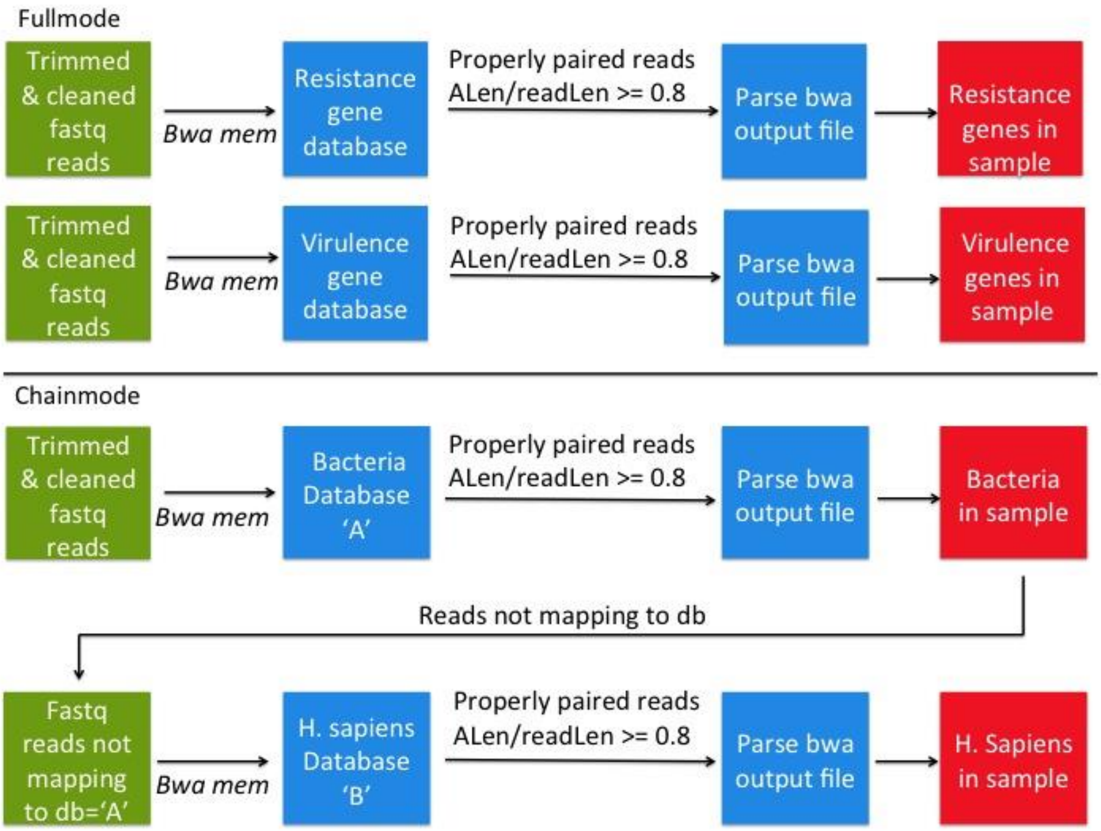

Supplementary Figure 4. The Fullmode and Chainmode methods are shown in top and bottom part of the figure, respectively. Trimmed and cleaned fastq reads is referred to as reads processed with cutadapt and subsequently only reads that can still be paired up are selected as a start set of fastq reads. In Fullmode that set is always the starting point for mapping against one or more reference sequence databases. In Chainmode, an ordered chain of databases is specified and reads not mapping to the first database, will get another chance to map to a database further down the chain. 'Alen', the alignment length is sum of matches 'M' which is extracted from the output of bwa in the so-called cigar strings where a match 'M' can be either an alignment match or a mis-match. If a read or its mate align to a database with an Alen/ReadLen below 80%, then both reads are discarded and enter the pool of unmapped reads that in Chainmode can be mapped against the next database in the chain.
